# Mobile Health in Home-Based Palliative Care: Realist Review of Contextual Factors and Mechanisms Influencing Outcomes

**DOI:** 10.2196/80321

**Published:** 2026-08-14

**Authors:** Nuhamin Tekle Gebre, Oladayo Afolabi, Nahla Gafer, Kennedy Bashan Nkhoma, Nicola Ayers, Charlotte Hanlon, Richard Harding

**Affiliations:** 1 Cicely Saunders Institute, Florence Nightingale Faculty of Nursing, Midwifery and Palliative Care, King's College London, Bessemer Road, London, SE5 9PJ, United Kingdom, 251 911977550; 2 Global and Planetary Health Working Group, Institute of Medical Epidemiology, Biometrics, and Informatics, Center of Health Sciences, Medical Faculty, Martin Luther University Halle-Wittenberg, Halle (Saale), Germany; 3 Division of Psychiatry, Institute for Neuroscience and Cardiovascular Research, University of Edinburgh, Edinburgh, United Kingdom; 4 Department of Psychiatry, School of Medicine, College of Health Science, Addis Ababa University, Addis Ababa, Ethiopia

**Keywords:** community palliative care, home-based palliative care, digital health intervention, mobile health, mHealth intervention, telehealth

## Abstract

**Background:**

The increasing prevalence of chronic illness presents a significant global health challenge due to growing end-of-life suffering. Palliative care is now an essential health service under Universal Health Coverage. Its integration into primary health care and use of mobile health (mHealth) have been recommended to improve access.

**Objective:**

This study aimed to synthesize evidence on how mHealth interventions in home-based palliative care work, for whom, and in what context.

**Methods:**

This realist review identified global evidence relevant to mHealth interventions for home-based palliative care through searches of 9 electronic databases (ie, MEDLINE, Embase, PsycINFO, Global Health, CINAHL, Web of Science, Cochrane Database of Systematic Reviews, Scopus, and Global Index Medicus), and forward citation tracking of included articles conducted in March 2026. The review followed the 5 key stages of a realist review: scoping the literature to assess what is important about the context of mHealth intervention in home-based palliative care and what mechanisms might be important in how such interventions result in their intended outcomes; articulating the underlying program theory and refining the review scope through consultation with international palliative care experts; conducting iterative searches for and appraisal of relevant evidence; extracting data; and narratively synthesizing the data, prioritized by relevance and rigor to generate conclusions and recommendations. The Framework of Complexity in Palliative Care Context, adapted from Bronfenbrenner’s Ecological Systems Theory, was applied to examine the contextual influences and interactions among factors within the multilayered system. Context-mechanism-outcome configurations were developed and iteratively tested to refine the program theory for mHealth in home-based palliative care.

**Results:**

A total of 4134 records were identified, of which 422 (10.21%) articles were retained for full-text screening, and 126 (3.05%) studies were included in the final synthesis. The contextual factors and mechanisms that positively influence the intended outcomes include (1) alleviating concerns and mitigating perceived threats about mHealth’s suitability in palliative care through proper orientation for patients and carers, along with clear guidelines for health care professionals; (2) minimizing infrastructural and technological barriers through user-friendly designs and investment in sustainable models of mHealth for home-based palliative care; (3) engaging diverse stakeholders to ensure mHealth aligns with priority health care needs, user preferences, and the operations of implementing organizations and the wider health care system; (4) streamlining health management information systems through interoperable mHealth systems implemented at different levels of care; (5) enabling access to and regular communication with relevant health care teams; and (6) facilitating access to adequate information to empower users in palliative care services.

**Conclusions:**

mHealth interventions can enhance home-based palliative care but must align with local contexts. It is recommended that mHealth interventions ensure safety and comfort, technology competence, tailored communication, empowerment of end users, family involvement, health care worker motivation, and system integration.

## Introduction

The World Health Organization (WHO) advocates for digitally enabled care pathways to improve health care delivery through accurate data capture, enhanced communication, and care coordination [1]. Digital health interventions (DHIs) encompass a wide range of information and communications technologies that support health and health-related activities [2]. Mobile health (mHealth) is a subset of DHIs that uses mobile devices, such as mobile phones, patient monitoring devices, personal digital assistants, and other wireless devices, in medical and public health practice [3,4]. In palliative care, this has been achieved through the use of patient-reported outcome measures and electronic health recordings [5,6]. During the COVID-19 pandemic, mHealth facilitated the remote monitoring and management of patients’ symptoms, ensuring continuity of palliative care delivery [7,8].

mHealth technologies have demonstrated a positive impact on patient education, information sharing, communication, and clinical decision-making, as well as saving costs [9]. However, significant heterogeneity has been reported in the results of prior systematic reviews of mHealth effectiveness [3,10]. Challenges in the implementation of mHealth interventions include poor access to mobile devices and networks, usability and interoperability issues, and user concerns about data security and privacy [11,12]. Moreover, mHealth interventions are complex due to their multiple interacting components, the interplay of contextual factors, and the range of systems within which they are implemented [13]. To date, there is a paucity of research developing theoretical models of how these complex interventions improve health, psychosocial, and organizational outcomes or how contextual factors may influence their effectiveness [4]. Such evidence is needed to inform the development and evaluation of mHealth interventions for home-based palliative care and to ensure they can be successfully integrated into existing services.

Palliative care is an essential health service under Universal Health Coverage. However, of 56.8 million people who require the service every year, only an estimated 12% of them have access to it. Currently, most provision is in high-income countries, while 80% of the need is in low- and middle-income countries (LMICs) [14,15]. Palliative care aims to “improve the quality of life for patients and their families facing problems associated with life-threatening illness [16,17], through the prevention and relief of suffering by means of early identification and impeccable assessment and treatment of pain and other problems, physical, psychosocial and spiritual” [18-21].

Among the main strategies identified to enhance access to and improve coverage of palliative care are its integration into primary health care and the use of mHealth technology. Integrating health services within primary care is recognized as the most inclusive, equitable, and cost-effective strategy to achieving comprehensive health care coverage [22]. This approach enhances equitable access to services, especially for patients in remote or underserved areas, by enabling them to receive care in the comfort of their own homes. Furthermore, it ensures continuity of care, providing consistent and coordinated support throughout the illness trajectory, which contributes to better patient outcomes.

Leveraging technological interventions, such as mHealth into primary health care, enables timely and effective support for those in need, particularly patients in underserved regions [13,23,24]. The aim of this review was to determine how mHealth interventions improve both access to and the quality of home-based palliative care and to identify the contextual factors and mechanisms of action underlying their intended outcomes.

## Methods

### Design

In this realist review, we undertook a theory-informed synthesis of evidence on how, why, and under what circumstances mHealth works in home-based models of palliative care. The rationale for the design was that realist reviews provide an explanatory focus to reveal the mechanisms by which complex interventions work in relation to various contexts, allowing the development of a model that demonstrates how and why interventions work [25,26]. Realist methodology generates program theories using context-mechanism-outcome configurations (CMOCs), which are the main output of the review. Contexts (C) describe the circumstances in which the intervention is implemented, mechanisms (M) describe how the intervention works within specific contexts, and the interaction between these will lead to a particular outcome (O) [27,28].

This review adhered to the 5 main stages of a realist review, comprising scoping the literature, articulating underlying program theory and refining the scope of the review with stakeholders, searching for and appraising relevant evidence, extracting data, and synthesizing data to draw conclusions and recommendations [29]. The Realist and Meta-Narrative Evidence Synthesis: Evolving Standards were used to guide reporting [27,28]. The protocol for this review was registered on PROSPERO (registration CRD42022369443).

### Procedure

#### Scoping the Literature to Develop Preliminary Hypotheses

First, a scoping search was undertaken to explore the context of palliative care needs at home, what is important about supporting such needs, how end users might engage with mHealth interventions, and the mechanisms underpinning home-based care. Nine electronic databases were systematically searched: MEDLINE, Embase, PsycINFO, and Global Health via Ovid Technologies, CINAHL via EBSCO Information Services, Web of Science via the Clarivate Web of Science platform, the Cochrane Database of Systematic Reviews via Cochrane Library, Scopus via the Elsevier Scopus, and Global Index Medicus via the WHO platform. The search applied a broad structure: [mHealth] AND [palliative care] AND [home-based care], combining subject headings and free-text searches of titles and abstracts. Search results were uploaded to an online data management software, Covidence [30]. The first author (NTG) screened all records for relevance to the topic of mHealth intervention in home-based palliative care, while the second and third reviewers (OA and NG) independently screened 50% of the identified articles, and any disagreement was resolved by team discussion. After title, abstract, and full-text screening, the authors familiarized themselves with the included studies and identified common themes. Preliminary *“if...then”* statements were developed to articulate how, why, and under what circumstances mHealth interventions influence home-based palliative care. These statements informed the initial context-mechanism-outcome hypotheses [29].

#### Articulating Underlying Program Theory and Refining the Scope of the Review With Stakeholders

The preliminary hypotheses generated from the scoping search were discussed with 14 global palliative care researchers and health care professionals through individual face-to-face and online meetings [31]. These discussions emphasized the importance of understanding the complex systems in which mHealth interventions are implemented, including their impact on technology engagement and subsequent outcomes.

**Figure 1.**
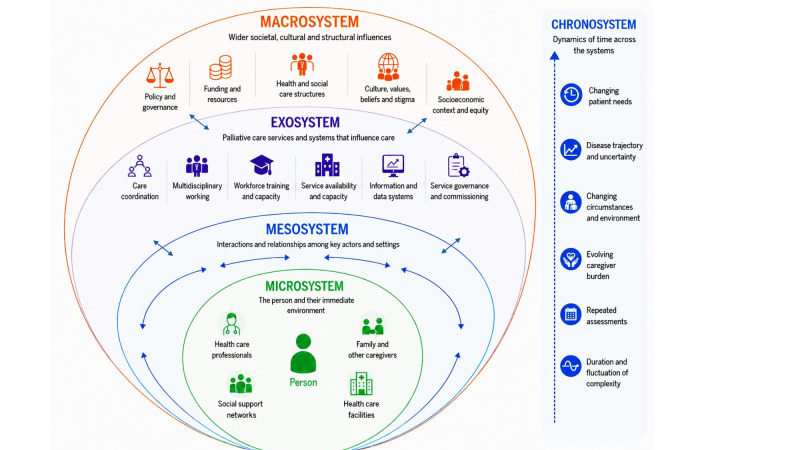
A framework of complexity in the palliative care context. Key: These levels are at the microsystem (person, needs, and characteristics), the chronosystem (dynamic influences of time), the mesosystem (interactions with family and health care professionals), the exosystem (palliative care services or systems), and the macrosystem (societal influences) (adapted from Pask et al [32], which is published under Creative Commons Attribution-NonCommercial 4.0 International License [33]).

To explore these contextual factors, we applied the Framework of Complexity in Palliative Care Contexts [32] adopted from Bronfenbrenner’s Ecological Systems Theory [25,26]. This framework highlights interconnected environmental levels and provides a systemic perspective to analyze variations in context, multisectoral actors, and environments across different levels of the health care system (Figure 1). Insights from stakeholder consultations guided the refinement of the review scope and informed the subsequent iterative search strategy.

#### Identify and Appraise Relevant Evidence

Following stakeholder consultation, an iterative evidence database search was conducted to identify studies relevant to the refined scope. The aim of this supplementary searching was to reach a point of theoretical saturation, where no new relevant evidence was generated for the particular theory building or testing within the synthesis [29].

The iterative search applied refined search terms informed by the scoping search and reassessment of the retained studies. In addition to the broader terms, specific functions of mHealth interventions commonly used in home settings, such as telehealth, telemedicine, and videoconferencing, were included. Searches were conducted in the same 9 electronic databases, with no restriction by year in March 2024 (full search strategy reproduced in Multimedia Appendix 1). The search results were uploaded and managed in Covidence. Only papers published in English were considered due to the practical constraints of engaging reviewers fluent in other languages.

In March 2026, the search was updated with forward citation searching of the final included studies using Google Scholar to identify more recent publications. Screening was conducted following predefined selection criteria; the first author (NTG) screened all titles and abstracts, while OA and NG independently reviewed 50% of records. Discrepancies were resolved through team discussions.

### Eligibility Criteria

The predefined selection criteria are described in Table 1.

**Table 1.** Summary of the eligibility criteria for all studies included in the review.

Eligibility domain	Inclusion criteria	Exclusion criteria
Participants or population	Patients, both adult and pediatric of all ages, with life-threatening conditions^a^ or life-limiting illnesses^b^, which are chronic conditions, which significantly impair quality of life and daily functioning, necessitating ongoing management to alleviate symptoms and provision of supportive care (eg, advanced dementia, progressive neurological diseases, and end organ failures [17]), or their informal caregivers (ie, *unpaid providers of physical, social, practical, and emotional support who may be blood or non–blood relatives* [34]), or formal (paid) health care professionals, community health workers, or health extension workers.	Patients solely treated within acute care or receiving only disease-modifying or curative treatment; and caregivers and health care providers with no palliative care roles
Intervention	mHealth^c^ is a subset of DHIs^d^ that uses mobile devices, such as mobile phones, patient monitoring devices, personal digital assistants, and other wireless devices in medical and public health practice [1,2,35]. Studies were included if they featured the practice of palliative care supported by text message, voice or video call, or application-based or web-based technology (eg, through purposely designed apps or social media platforms) using mobile phones or tablets.	Interventions that do not use mHealth or with a limited mHealth application as part of a multifaceted intervention
Context	Home-based palliative care as a service delivered to seriously ill patients in the setting that a patient calls “home” (ie, a private residence) [36,37]. Studies were included irrespective of country or region to ensure representation of diverse sociocultural contexts.	Facility-based services (eg, primary health care facilities, hospitals), nursing homes, long-term care homes, and hospices (eg, outpatient, day care, and in patient)
Outcome(s)	All outcomes associated with mHealth-supported home-based palliative care; relating to patients, informal caregivers, or health systems.	No restriction

a Life-threatening conditions refer to conditions associated with a high risk of mortality.

b Life-limiting illnesses refer to chronic or progressive conditions that substantially impair quality of life and daily functioning, requiring ongoing symptom management and supportive care.

c mHealth: mobile health.

d DHI: digital health intervention.

### Quality Assessment Based on Relevance and Rigor

#### Appraisal Process and Prioritization

The relevance of studies in this review was assessed by considering whether they contained sufficient detail and/or theoretical discussion to inform how and in what context mHealth interventions support home-based palliative care, that is, their contribution to generating program theory [29,38]. In accordance with previous research, the relevance of contributions was defined as high (well aligned to the review questions, with rich descriptions contributing to theory), medium (with some relevance to the review question and some insights to CMOCs), or low (meeting broad inclusion criteria and providing at least one insight) [29].

The Mixed Methods Appraisal Tool (MMAT) [39] was used to assess the methodological quality and rigor of the included studies, as it is useful to assess the quality of qualitative, quantitative, and mixed methods studies. The MMAT comprises 5 quality criteria, with 3 responses for each item (ie, positive [=1] and negative or unavailable information [both=0]). The 5 items are added with a score of 5 indicating high quality. The Joanna Briggs Institute quality assessment tool was used to assess the methodological rigor of the included systematic reviews, with scores allocated on a scale from 0 to 11. Using this tool, a score of 8 or more indicates high quality, a score of 6 to 8 indicates moderate quality, and a score less than 6 suggests low quality. Three authors (NTG, NG, and OA) independently assessed the methodological quality of the studies and met to discuss their appraisal. Differences were resolved through discussion with the entire research team [40].

As a realist review approach may use only relevant data from part of an article, no studies were excluded based on methodological quality. However, studies with high relevance and rigor were given priority during theory development [27-29,31].

#### Data Extraction

Bespoke data extraction forms were developed to organize information obtained from the included studies. A careful review of the introduction, methods, results, and discussion sections assisted in identifying relevant information. Descriptive characteristics, such as author, year, study aims, design, population, and country, were recorded. Details were also extracted on the intervention components, contextual factors, implied or actual mechanisms attributed to the outcome of interest, and the intervention outcomes. Data extraction was undertaken by 2 of the authors (NTG and NG) working independently. Areas of discrepancy were resolved through discussion to reach consensus.

#### Data Synthesis

Evidence sources were synthesized narratively, prioritizing included studies by relevance and rigor [41] and shown as a set of CMOCs. Taking an iterative approach, the first author read and reread different sources of data and coded the evidence to identify prominent recurrent concepts concerning contexts, mechanisms, and outcomes [29]. Coding was done deductively to the concepts in the Framework of Complexity in Palliative Care Context and using the priority “if...then” statements, which informed the preliminary context-mechanism-outcome hypotheses. In this process, we drew logical conclusions from the observed patterns, particularly the contextual factors influencing mHealth interventions [32] and inductively (proposing insights from recurring observations), mainly regarding mechanisms, and retroductive coding (exploring new patterns using theory to propose causal pathways) [42]. This process allowed the development of mHealth program theory in palliative care and conceptualization of the causal pathways using contexts, mechanisms, and outcomes [43,44].

## Results

### Search Yield and Characteristics

#### Overview

**Figure 2.**
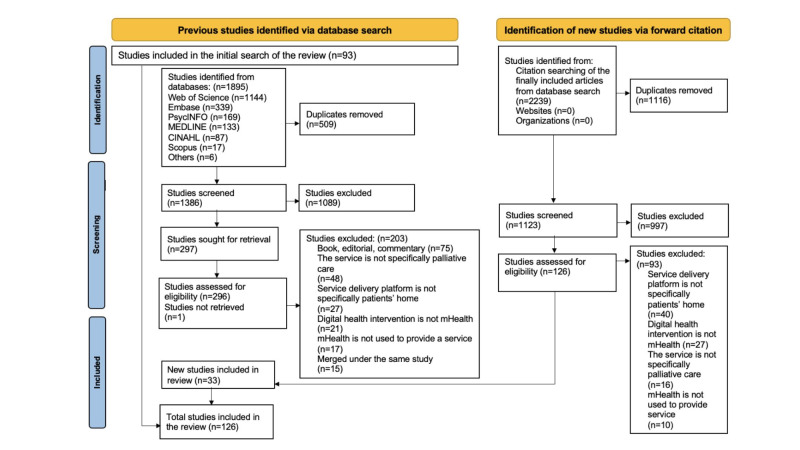
PRISMA (Preferred Reporting Items for Systematic Reviews and Meta-Analyses) flow diagram of articles included in the review. mHealth: mobile health.

In total, 4134 studies were identified via database searching (n=1895, 45.8%) and forward citation searching of the final included studies in March 2026 (n=2239, 54.2%). The selection process and included articles are illustrated in the PRISMA (Preferred Reporting Items for Systematic Reviews and Meta-Analyses) flow diagram (Figure 2). Following the removal of duplicates and initial screening, 422 (10.21%) articles were retained for full-text screening, and 126 (3.05%) studies were included in the final synthesis, and of these, 20 (0.48%) articles from the initial scoping search contributed to the initial preliminary hypothesis generation. Details of the studies included in the final synthesis are presented in Table 2.

**Table 2.** Studies included in the review.

Author, year, country	Aim	Study design	Intervention
Abahussin et al, 2023, United Kingdom [45]	To design and develop an mHealth^a^-based pain recording system to support effective pain management in cancer.	User-centered design and usability testing	A mobile app and a web-based app for adult patients with cancer in home settings to record pain details. The data were accessed by health care professionals and researchers.
Allsop et al, 2019, 21 countries in Africa (see Multimedia Appendix 1) [35]	To assess the current use of mHealth in palliative care service delivery in the African region, identify barriers to mHealth use, and determine provider priorities for research development.	Mixed methods descriptive design	Mobile phone.
Al-Mondhiry et al, 2022, United States [46]	To use community-partnered participatory research to co-design and pretest a mobile app that focuses on palliative care priorities of clinicians and patients with advanced cancer.	Co-design workshops	Mobile app, that is, wellness journal to track patient-reported symptoms, goals, and medication use; information on self-management of symptoms; community resources; and patient and caregiver testimonial videos.
Ansari et al, 2022, United States [47]	To identify technology-based communication strategies to improve health outcomes in individuals with advanced cancer.	Integrative review	Mixed technologies, for example, health information technology or electronic health reporting, data sharing systems, and telehealth programs.
Aoki et al, 2006, Japan [48]	To evaluate the implementation of telepalliative care in a rural community for patients with terminal cancer who prefer home-based palliative care.	Mixed methods study	Telepalliative care using a Polycom view station with ISDN^b^ line for communication, including telephone calls among health care providers and patients, and recording of vital signs.
Archer et al, 2021 [49]	To explore the impact of digital health interventions on the psychological outcomes of patients and families receiving pediatric palliative care.	Systematic review	Telehealth consultations via video link and telephone, telehealth intervention via Zoom videoconferencing or FaceTime, and the use of a bespoke website (My-Quality.net) to collect data on patient-reported outcomes.
Baldwin et al, 2012, United States [50]	To determine what organizational factors might account for differences in the use of telehospice.	Survey	Telehospice (ie, video conferencing) used by hospice agency staff and patients.
Balasubramanian et al, 2022, India [51]	To assess the level of satisfaction of patients receiving e-palliative homecare services and to evaluate the feasibility of e-palliative care in providing palliative care to patients with advanced cancer at their homes.	Prospective observational study	Implementation of e-palliative care through a technological interface, where a homecare team led by a palliative care–trained nurse visits patients with a camera-integrated laptop and e-palliative care software app installed, allowing remote communication with a palliative care physician in the hospital.
Bandini 2025, United States [52]	To examine current perspectives from interdisciplinary providers on the use of telehealth and in-person care for outpatient palliative care among underserved patients.	Qualitative study	Telehealth use among staff serving underserved population.
Basile et al, 2024 [53]	To map the literature on the use of technology by older adults with terminal illness receiving palliative care at home and to identify the technology systems in use, the impact of technology on communication between palliative care professionals and patients, and explore the strengths or weaknesses perceived by patients regarding the use of technology.	Scoping review	Tablets, smartphones, computers, videoconferencing devices, a pen with an embedded camera, and devices for remote symptom monitoring such as smartphones and wristbands.
Bauer et al, 2026, Denmark [54]	To develop program theory for a family-focused model of telepalliative care for adult patients and their families.	Qualitative study—workshops with key stakeholders, participant observations, and ethnographic interviews	Telepalliative consultation perceptions among patients with palliative care needs, family caregivers, SPCT^c^ members, allied health care personnel, community care nurses, IT personnel, and health care managers from both community and tertiary care settings.
Bensink et al, 2004, Australia [55]	To design and test an internet-based videophone for pediatric palliative care services, focusing on assessing the feasibility and benefits of using the technology for telemedicine in home settings.	Feasibility study with qualitative exploration	Videophone: use of low-bandwidth, internet-based videophone for supporting pediatric palliative care in the home, with clinical consultations conducted twice each week.
Bensink et al, 2009, Australia [56]	To investigate the acceptability of providing videotelephone-based support to families receiving pediatric oncology-related palliative care and to explore the integration of videotelephone support from the time of diagnosis through outpatient care and support.	Randomized controlled trial	Access to a videotelephone service in addition to the usual 24-h on-call telephone support service, provided by specialist nurses for patient assessment, monitoring, family education, communication, and counseling.
Bethel et al, 2021, United States [57]	To evaluate technology system usability and Comprehensive Patient Assessment for Using Telehealth at Home implementation effectiveness in seriously ill older adults and their caregivers.	Qualitative study	Comprehensive Patient Assessment for Using Telehealth at Home intervention to meet remotely and assess patients in their own home.
Bhargava et al, 2021, Canada [58]	To develop and evaluate a remote symptom self-reporting app (ie, RELIEF) for community patients with palliative care needs, assessing its feasibility and acceptability for remote monitoring of patients through regular symptom self-reporting, with a focus on using technology to improve symptom management and care delivery.	Co-design and feasibility study	Patients self-reported symptoms daily using RELIEF and received over-the-phone assessments and interventions based on alerts, and some received support for expected death in the home.
Bonsignore et al, 2018, United States [59]	To describe and evaluate a telehealth palliative care program using the TapCloud app and videoconferencing in rural areas, focusing on feasibility, usability, symptom burden, and hospice transitions.	Mixed methods study	TapCloud app for remote patient monitoring, including symptom and medication management, messaging, and photo upload, as well as videoconferencing for real-time interactions.
Bradford et al, 2014, Australia [60]	To compare the costs of pediatric palliative care medical consultations conducted via the Home Telehealth Program with in-person consultations.	A cost minimization analysis	Palliative care consultations through the Home Telehealth Program at the Royal Children’s Hospital in Brisbane, involving video consultations in the home.
Bradford et al, 2014, Australia [61]	To investigate the potential of telemedicine for the delivery of specialist support in the home or local community for children with life-limiting conditions requiring palliative care.	Qualitative study	Real-time audiovisual communication links between the specialist pediatric palliative care team and families caring for a child receiving palliative care at home, facilitated by personal computers, web cameras, and the internet.
Brännström et al, 2024, Sweden [62]	To describe health care professionals’ experiences of video consultations in palliative care in community homecare and nursing homes in rural areas.	Qualitative study	Video consultations to access specialist palliative care service.
Burner-Fritsch et al, 2023, Germany [63]	To identify and explore challenges for the development of electronic patient-reported outcome measures as standardized assessment in specialized palliative home care, focusing on patient, health care professional, and organizational or structural levels.	Qualitative study	Patients used the electronic version of the Integrated Palliative Care Outcome Scale on their own web-enabled devices, completing it 1 to 2 times a week for at least 2 weeks.
Caetano et al, 2024, Portugal [64]	To explore whether caregivers feel prepared to provide informal palliative home care, their experiences, and the usefulness of telehealth in managing daily activities.	Qualitative study	Telehealth to support informal caregivers.
Calton et al, 2020, United States [65]	To characterize the experience of patients with serious illness and their caregivers receiving palliative care by telemedicine.	Mixed-methods telephone survey	Telemedicine acceptability and comfort among patients and caregivers.
Cameron 2021, United States [66]	To examine the comfort and emotional support that caregivers of home hospice patients derive from using electronic tablets for telehospice.	Mixed methods study	Caregivers received electronic tablets with the AVA^d^ app for telehospice participation, were given a presurvey, shown how to use the tablets, completed surveys after setting up AVA in the home, and received a postsurvey 2 wk after the death of the loved one.
Carey et al, 2023, Ethiopia [67]	To co-design, develop, and evaluate a mobile phone–based remote monitoring system for use by palliative care patients in Ethiopia.	Co-design and qualitative study	Remote monitoring system where patients and families record their symptoms and access information to self-support their needs.
Christiansen et al, 2020, Sweden [68]	To describe perceptions of mHealth and its impact on HRQoL^e^ among older adults with cognitive impairment.	Qualitative study applying phenomenography	mHealth intervention (SMART4MD) to improve the HRQoL of older adults with mild cognitive impairment and their informal caregivers.
Chua et al, 2022, United States [69]	To describe trends in in-person and video visit palliative care delivery challenges before and during the COVID-19 pandemic in the United States.	Quantitative nonrandomized	Early integrated telehealth palliative care (via video visit) or in-person palliative care, with at least monthly visits; initial visit in person within 4 wk of enrollment but could be conducted via video during the COVID-19 pandemic; subsequent follow-up visits every 4 wk, either via video or in person.
Collier et al, 2016, Australia [70]	To explore clinicians’ perspectives and experiences of telehealth, perspectives about benefits and challenges for patients, families, and clinicians in using telehealth applications, and identified enablers and barriers for the use of telehealth in community palliative care.	Qualitative study	The intervention included self-report assessment tools, remote activity monitoring, video-based conferences, and virtual case conferences.
Collier et al, 2025, Australia and Aotearoa, New Zealand [71]	To explore the complexities of telehealth and palliative care with a focus on access and equity.	Qualitative study	Telehealth for extending reach of palliative care in underserved groups.
Cook et al, 2001, United States [72]	To analyze the successes and challenges of implementing telehospice care focusing on the role of senior executives’ support, quality of care, and provider acceptance.	Observational study	Implementation of telehospice services using telemedicine technology, including training of potential telehospice users and providing care remotely via videophones over ordinary telephone lines to patients in rural and urban communities in Kansas and Michigan for at least a year.
Coyle et al 2002, United States [73]	To explore the effectiveness and benefits of using audiovisual communication as a complementary tool in caring for patients with complex palliative care needs who are isolated at home due to their medical conditions, aiming to enhance communication and support for both the patients and their caregivers.	Case report of a 3-mo trial	Trial of a commercially available audiovisual system, specifically a videophone (TeleEye 324 TM; LeadTek Research, Inc), placed in the patient’s home using standard telephone wires for a 3-mo trial period.
Cruz et al 2023 [74]	To map the available knowledge regarding the use of apps for mobile devices to support adult patients in palliative care at home, specifically focusing on mHealth apps under study or development in the home care transition process for palliative care patients.	Systematic review	Participants received the mobile app intervention for monitoring symptoms, communication with health care professionals, and potentially receiving alerts and educational materials related to palliative care. The intervention was compared with controls in some studies, and outcomes were assessed in terms of changes in daily functioning, self-efficacy, and quality of life.
Davis et al, 2015, United States [75]	To describe the telecaring intervention and its outcomes, focusing on proactively identifying and addressing the needs of patients receiving hospice care at home and caregivers through a systematic phone-based care service.	A quality improvement initiative	TeleCaring consisted of daily proactive phone calls to patients and caregivers by specialists and nurses, made up to twice daily based on the patient or caregiver’s preferred frequency and timing, providing up to 14 additional staff interactions each week. The calls aimed to address questions or concerns related to care and ensure supply needs were met.
Dickman Portz et al 2020, United States [11]	To explore provider perspectives regarding the utility of mHealth in palliative care.	Qualitative study	mHealth perception of use among providers from multiple disciplines working in palliative care settings.
Disalvo et al, 2021 [76]	To identify digital health technologies evaluated for supporting timely assessment and management of people with palliative care needs at home and their carers.	Meta-review	Remote patient monitoring interventions included monitoring of biometric data or symptoms, with alerts triggered to clinicians when patient scores were outside acceptable thresholds.
Dismore et al, 2025, United Kingdom [77]	To understand the experiences of patients, carers, and specialist palliative care professionals to receive or deliver video consultations in a rural setting.	Qualitative study	Video consultation perception of use to access specialist palliative care.
Dolan et al, 2021 [78]	To assess the safety and effectiveness of virtual care in end of life and palliative care.	Review	Virtual care, that is, video consultation, mobile apps, videos, websites, telephone support, email, and alert messages.
Domínguez et al, 2026, Ecuador [79]	To evaluate the technology usability and implementation feasibility of the nonintrusive devices for telemedicine system, a low-cost, smartwatch-based pain monitoring solution for palliative cancer care co-designed with health care staff from a cancer hospital in Ecuador.	Usability and feasibility study	Wearable-based pain monitoring system.
Doolittle et al, 2019, United States [80]	To explore the feasibility and effectiveness of using a TeleHospice approach in rural hospice care, focusing on enhancing patient- and family-centered, timely care.	Product design—community-engaged model	Use of low-cost, secure mobile tablets (iPads) for videoconferencing encounters in rural hospice care settings, involving an interprofessional team.
Evering et al, 2022, The Netherlands [81]	To explore the intention to use video communication by health care providers in interprofessional terminal care settings.	Cross-sectional survey	A video communication in terminal care.
Gonzalez, 2023, United States [82]	To evaluate the perception of usability of a tablet device among palliative care patients and informal caregivers.	Cross-sectional pilot study	A home health tablet device.
Gordon et al, 2022 [83]	To explore strategies for increasing access to palliative care among individuals living in remote or rural communities through the use of telehealth applications.	Rapid review	The interventions included videoconferencing, telephone-based interventions, web-based platforms, after-hours phone support service, remote patient monitoring and management, web-based videoconference platforms, and 24/7 phone consult services.
Guo et al, 2023, China [84]	To examine telehealth readiness and its related factors among Chinese palliative care specialist nurses.	Cross-sectional study	Telehealth (readiness assessed using the Chinese version of Telehealth Readiness Assessment Tools and the Innovative Self-Efficacy Scale).
Hancock et al, 2019 [85]	To describe the current use of telehealth in palliative care in the United Kingdom, evaluate telehealth initiatives against a digital service standard, and explore whether telehealth results in a reduction in emergency care access.	Systematic review	The intervention(s) was home telemonitoring using telephone or computer software to record clinical symptoms or signs from the patient’s home. Patients were required to input specific data regarding symptoms and physical parameters, with some studies including telephone support.
Harding et al, 2021, India, Uganda, and Zimbabwe [9]	To design and evaluate a mobile phone app to improve communication between family caregivers, community caregivers, and palliative care teams, assess its acceptability and mechanisms of action, and propose refinements based on stakeholder feedback.	Mixed methods study	The intervention involved the use of a mobile phone app for family or community caregivers to report nonurgent patient and family outcomes on a dashboard accessible by the clinical palliative care team in 3 LMICs. The app aimed to facilitate patient assessment, improve staff awareness of patient concerns, enhance communication, and potentially improve patient outcomes.
Haroen et al, 2025 [86]	To investigate the benefits of integrating telehealth into community-based palliative care for people living with terminal illness and their caregivers.	Review	Telehealth, that is, telephone or video consultations and phone calls.
Haydon et al, 2021, Australia [87]	To explore the costs, service activity, and staff experiences resulting from the introduction of telehealth in a community palliative care service in Queensland, Australia.	Mixed methods study	In-person and telephone-based home visits and telehealth (video consultations).
Hebert et al, 2007, Canada [88]	To determine the potential for using videophones in palliative home care and to explore factors that could inform the eligibility criteria for video visits.	Retrospective chart review	Palliative home care visits that could have been carried out using videophones, telehospice care as a support for family caregivers, testing the usability of a videophone in palliative home care, and determining the potential for home telehealth in palliative care.
Hennemann-Krause et al, 2015, Brazil [89]	To examine telemedicine as a form of home and additional support for traditional outpatient care as a way to remotely monitor and manage the symptoms of patients with advanced cancer.	Prospective, longitudinal, qualitative, descriptive, and case series study	Web conferences to remotely monitor and manage the symptoms.
Hoek et al, 2017, The Netherlands [90]	To determine whether weekly teleconsultations from a hospital-based SPCT improved patient-experienced symptom burden compared to “care as usual” in home-dwelling patients with advanced cancer.	Randomized clinical trial	Weekly teleconsultations for a period of 13 wk in addition to their usual care. First, a teleconsultation device was installed at the patient’s home. Patients who had not visited the SPCT before were evaluated at the outpatient clinic or during a home visit by one of the SPCT members.
Holland et al, 2014, United States [91]	To determine the resources needed to use the system and the quality of the audio and visual components to conduct virtual visits between a clinician at an academic center and community-dwelling adults living in rural locations.	Mixed-methods field study design	Virtual visits using a 3G-enabled Apple iPad, cellular phone data service, and a web-based video conference service.
Holmen et al, 2020 [92]	To identify and review the use of eHealth to communicate and support home-based pediatric palliative care.	Convergent, systematic mixed methods review	Interventions received by the study participants included videoconferences, the use of an online tool for monitoring quality of life, and the use of iPads for videoconferencing, among others.
Hutchinson et al, 2022, United States [93]	To explore the acceptability, feasibility, and emotional responsiveness of telepalliative care.	A mixed-methods formative study	Telemedicine perception of use, that is, acceptability, feasibility, and emotional responsiveness of telepalliative care.
Jess et al, 2019 [94]	To review and synthesize current evidence regarding the use of video consultations in both general and specialized palliative care to various patient groups.	Systematic integrative review	Use of video consultations in specialized palliative care settings
Jiang et al, 2023, Australia [95]	To assess the feasibility of a TH-SPC^f^ model for an Australian rural setting, focusing on integrating telehealth technology to provide specialist palliative care to rural communities.	Prospective mixed methods pilot study	Video consultations with metropolitan-located specialist palliative care physicians alongside standard care.
Johnston, 2011 [96]	To explore the use of telehealth in relation to palliative care in the United Kingdom.	Review	Telehealth use for older people.
Johnston et al, 2012, United Kingdom [97]	To evaluate the current use of telehealth applications within palliative care across Scotland and assess how these applications are perceived by patients, carers, and professionals.	Qualitative evaluation design	Telehealth activity among the key stakeholders centered on the use of videoconferencing equipment for networking, consultations, and multidisciplinary meetings, and for education and training.
Johnston 2014 [98]	To scope information available from published and unpublished research on the current state of palliative home-based technology, practitioner-focused perspectives, patient-focused perspectives, quality of life, and the implications for clinical practice.	Review	Home-based technology perspectives of use among clinicians and patients and implications on practice.
Funderskov et al, 2019, Denmark [99]	To explore the advantages and disadvantages of using video consultations in SPC^g^, focusing on the experiences of health care professionals and their perspectives on video consultations.	Qualitative study	Patients referred to specialized palliative care received an initial home visit by an SPC team head physician and an SPC team nurse. They were primarily in contact with the SPC team nurse by phone and had approximately one weekly video consultation using a tablet provided by the SPC team.
Kawashima et al, 2026 [100]	To evaluate the effectiveness of and implementation requirements for telehealth in palliative care patients with advanced cancer.	Review	Telehealth ie, videoconferencing, telephone, or text-based communication.
Keenan et al, 2021, United Kingdom [101]	To explore potential divergence and convergence in relation to health care professionals’ and patients’ acceptability of the use of telehealth within palliative care provision through the lens of Self-Determination Theory.	Qualitative study	Telehealth perceptions among health care professionals and patients.
Kidd et al, 2010 [102]	To review telehealth applications used in palliative care settings in the United Kingdom, identify who is using telehealth, for what purpose it is being used, and to explore if telehealth use is increasing within the clinical setting.	Systematic review	The interventions for health professionals, patients, carers, nurses, and GPs^h^ include telehealth services such as out-of-hours telephone support, advice services, videoconferencing, and training and education for health care staff.
Kluger et al, 2024, United States [103]	To determine whether palliative care training for neurologists and remote access to a palliative care team can improve outcomes in patients with Parkinson disease and related disorders in community settings, focusing on patient quality of life, caregiver burden, and other patient-centered outcomes.	A pragmatic, stepped-wedge comparative effectiveness trial	Palliative care education for community neurologists and provision of team-based palliative care resources via telehealth, including consultations from an academic center’s palliative care team.
Kwon et al, 2026, Republic of Korea [104]	To (1) develop the Hospice@Home system, a digital in-home hospice care solution; (2) explore preliminary indications of usability and feasibility among patients with terminal cancer and their caregivers; and (3) identify challenges for future implementation.	Development and usability pilot study	Digital in-home hospice care solution for patients with terminal cancer and their caregivers.
Middleton-Green et al, 2019, United Kingdom [105]	To evaluate the Gold Line telehealth initiative in providing support to individuals at home in the last year of life, focusing on patient and carer experiences and the extension of palliative care services to those not known to specialist palliative care.	Mixed methods evaluation	Intervention components: Gold Line service, training for health care teams, use of the Gold Standards Framework, access to electronic health records, recording call outcomes, and participation in interviews.
Lee et al, 2024, Ireland [106]	To understand patient experience.	Mixed quantitative and qualitative study	Video consultation—perception of use among patients.
Lind et al, 2008, Sweden [107]	To explore and describe palliative home care patients’ experiences of assessing their pain using a pain diary together with digital pen and mobile Internet technology.	A case study with cross-case content analysis	A pain diary together with digital pen and mobile Internet technology. Included introduction of pain management clinical guidelines, pain treatment for patients with at least moderate pain, and follow-up of pain treatment using the IT system developed.
Lundereng et al, 2023 [108]	To systematically map published studies on HCPs’^i^ experiences and perspectives on the use of telehealth in HBPC^j^.	Scoping review	Video-based technology for teleconsultations among patients, families, and health care professionals, delivered by hospital-based HCPs, hospice workers, and home care professionals either independently or in collaboration with hospital-based staff.
Ma et al, 2025 [109]	To provide a comprehensive understanding of the roles of nurses and the multilevel facilitators and barriers to implementing nurse-delivered telehealth in home-based palliative care, which could inform future policy development, research, and clinical practice.	Review	Telehealth for home-based palliative care delivered by nurses.
Mathews et al, 2023 [110]	To describe the components of telehealth palliative care interventions for patients with advanced cancer before the COVID-19 pandemic, identify intervention components associated with improvements in outcomes, and evaluate the reporting of interventions.	Scoping review	Hybrid models, telephone-based interventions, videoconference-based interventions, asynchronous web-based intervention, mainly consisting of psychosocial content and delivered by nurses in the home setting. Participants included patients, family caregivers, and patient-caregiver dyads.
Maguire et al, 2025, United Kingdom [111]	To explore the usability, user experiences, and impact of the digital dyadic remote monitoring Care and Support System for Patients and Carers for patients in the last year of life, their informal carers, and health professionals involved in their care.	Mixed methods feasibility study	Digital dyadic remote monitoring Care and Support System for Patients and Carers for patients in the last year of life.
Maudlin et al, 2006, United States [112]	To determine if a telehealth model of care would benefit veteran patients with life-limiting illness by managing their physical, emotional, functional, and spiritual care needs during the last 2 y of their lives and fostering earlier enrollment into hospice through provider education on palliative care.	Pilot testing of the Advanced Illness/Palliative Care program	Technologies used included both video (telemonitors and videophones) and nonvideo (disease management and messaging) devices.
Mccall et al, 2008, United Kingdom [113]	To test the acceptability and usability of the ASyMSp^k^ in monitoring and managing symptoms of patients receiving palliative care at home.	Explorative descriptive study	A mobile phone–based technology (ASyMSp) to monitor and manage symptoms reported by patients receiving palliative care at home, involving daily completion of an electronic questionnaire over a 30-d period.
Menon et al, 2015, United States [114]	To investigate the feasibility and describe patient outcomes of palliative care consultations via telemedicine in critically ill patients, assess the impact on decreasing the burden on family members, and provide care in alignment with patient wishes.	A retrospective review	Telemedicine palliative care conferences.
Miller et al, 2021 [115]	To explore the existing literature on pediatric TM-HBPC^l^ for children with serious illness to identify research, determine the effectiveness, and highlight the need for more robust information on implementation, adaptation, and maintenance of TM-HBPC models.	Systematic scoping review	The intervention involved the delivery of home-based palliative care for children with serious illness via telemedicine, typically conducted by a hospital-based pediatric palliative care multidisciplinary team, with varying session durations and scheduling, and the use of different platforms for telemedicine consultations.
Morse et al, 2021, Tanzania [116]	To design and develop mPCL^m^, a web and mobile app to support outpatient symptom assessment and care coordination and control, with a focus on pain.	Human-centered iterative design framework	Participants received the mPCL app for symptom assessment and care coordination, with some being provided loaned Android devices with the app preinstalled and personal smartphones installed the app. The intervention lasted for 4 mo.
Mukamal et al, 2025 [117]	To explore the experience of nononcologic older adult patients in palliative care with telemedicine	Review	Telemedicine to enhance palliative care for older adults.
Namasivayam et al, 2022 [118]	To review and map the available evidence on the use of telehealth in providing after-hours palliative care services in rural and remote Australia.	A scoping review	The intervention was the use of telehealth services provided by RNs^n^, nurse specialists, GPs, specialist physicians, after-hours coordinators, and managers to enable patients to continue receiving after-hours palliative care at home in rural and remote Australia.
Narvaez et al, 2022 [119]	To map and evaluate existing research on the use of telemedicine and telehealth in palliative care patients during the COVID-19 outbreak.	Integrative review	Telehealth interventions including video conferencing, remote monitoring, and novel ways to improve care at home.
Nguyen et al, 2020, United States [120]	To compare a tech-supported model with video consultations to a standard model of home-based palliative care to determine non-inferiority in terms of interprofessional team coordination and patient outcomes.	Pragmatic, cluster-randomized non-inferiority trial	The intervention included nurse-led home visits coupled with remote physician consultation supervision via video for patients and caregivers in the tech-supported arm. Training on video visit procedures was provided to clinicians.
Oakley-Girvan et al, 2021, United States [121]	To develop a mobile app (TOGETHERCare) that enables cancer survivors’ care partners to monitor the survivors’ health and provide resources for the care partners.	Product design study with user involvement	Use of the TOGETHERCare mobile app by cancer care partners for a 28-day feasibility field test to remotely monitor cancer survivors’ health and their own health, as well as to provide care partner resources.
Oelschlagel et al, 2021, Norway [122]	To explore municipal health care professionals’ experiences with implementing an app for remote care in palliative home care for patients with cancer.	Qualitative, descriptive, exploratory design	Implementation of a technological solution named “remote home care” in palliative home care for patients with cancer, including providing a tablet device, installing digital medical devices.
Parker Oliver and Demiris, 2004, United States [123]	To assess the readiness of hospice organizations to accept technological innovation, specifically focusing on the current use of information and communication technology by hospice employees and their receptiveness to telemedicine or other advanced technology in patients’ homes.	Cross-sectional survey study	Broad ICT^o^ use—no specific intervention.
Oliver et al, 2012, United States [124]	To assess the state of evidence related to telehospice services and to highlight the need for strengthening the evidence base through various research methodologies.	Mixed methods study	Telephone advice lines, videophones, personal digital assistants, pen tablets, computers, comprehensive training for providers in the implementation of telehospice projects.
Olver et al, 2005, Australia [125]	To understand nurses’ experience.	Qualitative study	Videophone communication among rural palliative care nurses and their more remote general nursing colleagues.
Osuji et al, 2020, United States [126]	To assess the usefulness and appropriateness of video visits in home-based palliative care from the perspective of physicians and nurses, anchored within the NASSS^p^ framework.	Comparative effectiveness trial	Video visits were used primarily for follow-up visits as opposed to admission/start-of-care; intervention: video visits for follow-up care in home-based palliative care, facilitated by a nurse connecting patients with remote physicians.
Paré et al, 2009, Canada [127]	To evaluate the effects of a telehomecare intervention implemented in an oncology and palliative care unit in Quebec, Canada, focusing on nurses’ satisfaction with the software app, perceived quality of care, and individual and group productivity.	Pre-post evaluation design	Implementation of the SyMO software with modules for care planning, developing treatment plan, nurse assignment, visit planning, route guidance, and documentation.
Parker et al, 2021, Australia [128]	To evaluate the usefulness of CarerHelp from the perspective of health professionals caring for individuals with life-limiting illnesses.	Quantitative study	A web-based online toolkit for carers.
Paul et al, 2019, Canada [129]	To gain a preliminary understanding of the experience of using mobile web-based videoconferencing for conducting in-home palliative care consults with older rural patients with life-limiting illness, assessing the clinical effectiveness, acceptability, service impact, and technical feasibility.	A descriptive, exploratory, proof-of-concept study	Palliative care consultations via mobile web-based videoconferencing with a palliative care physician consultant from their homes, with the presence of a palliative care clinical nurse specialist, and a home care nurse to manage the technology and support the patient. Participants also received training in the use of the technology.
Peñarrubia‐San‐Florencio et al, 2025, Spain [130]	To explore family perspectives and experiences with telehealth in PPC^q^, focusing on their needs, perspectives, concerns, and hopes to refine digital care models.	Qualitative study	Telehealth to support families caring for children with palliative needs.
Phillips et al, 2008, Australia [131]	To evaluate the effectiveness of a generic after-hours telephone support service for palliative care patients and their caregivers in a rural setting in Australia, as well as to explore the application of a computer-based Palliative Care Clinical Information System to assist with the transfer of patient information.	Mixed methods study	After-Hours Telephone Support Service provided to palliative care patients and their caregivers.
Phongtankuel et al, 2018, United States [12]	To examine informal caregivers’ receptivity and interest in using mHealth apps, along with identifying the app features caregivers perceived to be most useful in-home hospice care.	Qualitative study	mHealth apps with features such as video chat, medication/symptom tracking, and educational materials to improve communication and information exchange in home hospice care.
Pinto et al, 2017 [132]	To analyze the use of eHealth technologies and mobile apps in palliative care, focusing on the strengths, weaknesses, opportunities, and threats of these technologies.	Integrative literature review	eHealth technologies in palliative care used by adults, patients, nurses, physicians, family, carers, and older people.
Pinto et al, 2017, Portugal [133]	To introduce a web-based app for monitoring comfort in patients receiving palliative care.	Multiphase electronic app development process with pilot design	Monitoring comfort remotely using a web-based app, providing self-reported data on 11 comfort-related items on a Likert scale; using the app for 15 consecutive days, inputting data at least once a day; contacting the palliative care team in case of significant symptomatic distress; accessing the app through Android or Windows operating systems on smartphones, tablets, or computers; using the app to report symptoms and distress, with researchers monitoring the data.
Putranto and Rochmawati, 2020 [134]	To explore and synthesize evidence on cancer symptom management mobile apps for patients and their families, highlighting the role of nurses and the potential extension of mobile apps for managing other life-limiting illnesses.	Scoping review	Mobile apps for managing cancer symptoms at home. Nurses are assigned to observe and assess patient symptoms through the mobile app. The main function of the mobile app system is to record an assessment of patient symptoms, provide education and skills training, and offer video media adjusted to the reported symptoms.
Rana et al, 2025, United States [135]	To further explore adult patient and caregiver perspectives on telehealth, specifically the benefits of telehealth, trade-offs with in-person appointments, and the impact on patient care and family end-of-life preparations.	Qualitative study—single-arm prospective pilot study	Telehealth to support palliative care services for children and young adults with cancer.
Royackers et al, 2016, Canada [136]	To examine the experiences of informal caregivers who cared for a family member receiving palliative care as part of the eShift model of home care, focusing on their satisfaction with care delivery and ability to support their family members to die, at home.	Interpretive description methodology with semistructured interviews	eShift model of home-based palliative care using RNs, PSWs^r^, and point-of-care technology with education provided to PSWs in palliative care.
Sadang et al, 2023, India [137]	To investigate barriers and facilitators to implementing Early Integrated Palliative Care via telehealth by surveying palliative care clinicians participating in a large-scale multisite palliative care trial comparing in-person and video visit-based services.	Multisite randomized trial	Early Integrated Palliative Care via secure video and/or in-person visits by advanced practice providers or board-certified physicians specializing in palliative care and/or oncology, at least monthly, beginning within 12 wk of diagnosis of advanced lung cancer.
Salem et al, 2020, Lebanon [138]	To assess caregiver and provider perceptions of the safety and efficacy of the Distance Support Program of a home-based palliative care provider.	Qualitative study	Distance Support Program including linking patients to a primary provider, face-to-face encounter, 24/7 telephone access, provider-initiated contact, and communication via telephone, messaging, or videophone.
Salimian et al 2019, Iran [139]	To develop the initial version of a mobile app for cancer palliative care for the purpose of improving the quality of life of cancer patients.	Mixed methods study	The mobile app “Ghasedak” for cancer palliative care, which includes functionalities such as user training on cancer and palliative care, clinician appointment reminders, personal health tracking, user guide, app settings, and patient notes.
Saysell and Routley, 2003, United Kingdom [140]	To address the national shortage of consultants in palliative medicine in the United Kingdom by implementing a telemedicine project to provide access to consultant advice and support for clinical nurse specialists in palliative care.	Mixed methods study	Weekly 2-h teleconference sessions between CNSs^s^ and a consultant in palliative medicine to discuss patients with unresolved symptoms or significant palliative care issues.
Septian et al, 2025 [141]	To systematically synthesize the existing evidence from systematic reviews regarding: (1) the benefits of telehealth use on the quality of care and (2) the impact of telehealth on the quality of life of terminally ill patients.	Review	Telehealth to support terminally ill patients.
Schmitt et al, 2022, United States [142]	To describe the demographics of pediatric patients receiving telepalliative care, assess patient and family satisfaction with telepalliative care, and explore motives for using telepalliative services.	Retrospective EMR^t^ chart review	Telepalliative visits.
Schoppee et al, 2020, United States [143]	To evaluate the acceptability of using the PAINReportIt completed on Wi-Fi-enabled tablets in the homes of patients with end-stage cancer and their caregivers for reporting pain related to end-stage cancer.	Pretest/posttest study	The PAINReportIt tool on a Wi-Fi-enabled tablet in patients’ homes for reporting pain in real time to hospice nurses, with patients and caregivers completing the tool daily for a week.
Schuessler and Glarcher, 2024 [144]	To describe formal and informal caregivers’ opportunities in telepalliative care and explore the associated ethical and safety challenges.	Integrative review of qualitative and quantitative studies.	The interventions included teleconsultation, supported by a step-by-step teleconsultation intervention protocol, telehealth options via telephone or videophone, web-based information, or smartphone apps for patient self-management, and the ENABLE CHF-PC^u^ intervention involving face-to-face outpatient palliative care consultation and monthly telephone care coach sessions.
Slavin-Stewart et al, 2020, Canada [145]	To assess the feasibility of using FaceTime over a mobile cellular data network to provide improved access to specialist palliative care for patients in rural Nova Scotia, focusing on using low-cost technology to enhance access to palliative care services in underserviced areas.	Observational study	Participants received regular home-based visits from a palliative care nurse who used the FaceTime app to connect with the palliative care physician in Halifax. The nurse used an Apple iPad with the FaceTime app and a cellular data package during these visits.
Steindal et al, 2023 [146]	To critically appraise and synthesize evidence on patients’ use of telehealth in home-based palliative care, focusing on the advantages and challenges experienced by patients.	Systematic mixed studies review with a convergent design.	Telehealth technology for home-based palliative care, including video consultations in synchronous mode for teleconsultations and remote monitoring in asynchronous mode for symptom management.
Stern et al, 2012, Canada [147]	To explore family caregivers and palliative cancer patients’ patterns of use, perceptions of, and experiences with home telehealth in the context of palliative cancer care.	Mixed methods case study with a focus on qualitative methods	The intervention included specialist nurses available 24 h per day who communicated with patients and families using videophones, with optional remote monitoring of vital signs such as blood pressure, blood oxygen levels, and heart, lung, and abdominal sounds. Communication was done via telephone or videophone as required.
Sutherland et al, 2020 [148]	To evaluate the use of video consultations as an alternative to face-to-face visits in palliative care during the COVID-19 pandemic.	Systematic review	Video consultations using various technologies, cost reduction for patients, telehealth interventions (electronic patient records, telephone advice consultations, and telehealth monitoring), and home visits.
Tasneem et al, 2019, United States [149]	To assess patient perceptions on the impact of telemedicine video visits on care and to evaluate the ease of use and impact on health management of videoconference telemedicine for palliative care oncology patients in an urban setting.	Qualitative study	Telemedicine video visits conducted through web-based face-to-face videoconferences.
Tarbi et al, 2025, United States [150]	To explore if and how recommended in-person best practices for establishing human connection are adapted to the telehealth palliative care setting to enable discovery and description of practice innovations in this new care environment.	Qualitative study	Telepalliative consultations that support clinician connection-building communication practices in rural telepalliative care encounters.
Tieman et al, 2016, Australia [151]	To explore how telehealth can provide more regular engagement, continuous monitoring, and immediate access to resources for patients and families ultimately aiming to improve the efficiency and effectiveness of palliative care delivery in the community.	Prospective cohort study	Video-based conferences, virtual case conferences, self-report assessment tools, and remote activity monitoring.
Toenne et al, 2025, Germany [152]	To evaluate the acceptance and challenges of implementing teleconsultations among specialized outpatient pediatric palliative care health care professionals.	Survey	Telehealth.
Tong et al, 2022, United States [153]	To evaluate the experiences of remote volunteer palliative care consultants during the initial COVID-19 surge, examining motivations, emotional toll, lessons learned, and providing insights for future efforts in extending remote palliative care services.	Qualitative study	A palliative care virtual consultation program.
van Gurp et al, 2013, The Netherlands [154]	To describe elements of both the physical workplace and the cultural-social context of the palliative care practice, which are imperative for the use of teleconsultation technologies.	Qualitative study	Synchronous, audiovisual teleconsultation via webcam and iPad for patients with palliative care needs at home.
van Gurp et al, 2016, The Netherlands [155]	To investigate how teleconsultation supports the integration of primary care, specialist palliative care, and patient perspectives and services, as well as how patients and informal caregivers experience collaboration through teleconsultation.	Qualitative study	Teleconsultation between home-based patients with palliative care needs and a hospital-based SPCT conducted synchronously and audiovisual, including tripartite consultations between the patient, primary care physician, and SPCT.
Salvador Verges et al, 2022 [156]	To describe the current use of telemedicine in palliative care and assess stakeholders’ views on the initiatives implemented worldwide regarding digital service standards.	Scoping review	Telemedicine for patient and caregiver support, professional education, and routine follow-up in palliative care.
Vincent et al, 2022, Canada [157]	To explore the experiences and perceptions of community palliative care providers, patients, and caregivers who delivered or received virtual palliative care as a component of home-based palliative care during the COVID-19 pandemic.	Qualitative study using phone and video-based semistructured interviews	Virtual palliative care as a component of home-based palliative care during the COVID-19 pandemic.
Washington et al, 2009, United States [158]	To measure telehospice acceptance in a national sample of hospice professionals from various disciplines and to explore the usefulness and ease of use of telehospice technology among different disciplinary backgrounds.	Cross-sectional study	The intervention was the use of videophone technology in the workplace for telehospice services, as demonstrated to participants through a web-based streaming video.
Weaver et al, 2021, United States [159]	To determine the acceptability of telehealth inclusion of a familiar pediatric palliative care provider during the first 2 home-based hospice visits to children, families, and adult-trained home hospice nurses in rural settings.	Case series	Virtual transition to home program with the inpatient pediatric palliative care physician joining the home hospice nurse’s in-person visit as a telehealth presence using a FaceTime screen on day 1 and day 14 at home.
Whitten et al 2004, United States [160]	To investigate the use of telehospice services from patients’ perspective, focusing on how telehospice is used, reasons for patient declination, and patient satisfaction and preferences regarding telehospice care.	Mixed methods	Telehospice services provided via videophones transmitting real-time video and audio images through the analog phone system, including nursing, social work, and spiritual care televisits.
Williams et al, 2024, United States [161]	To compare the provision of pediatric palliative care by delivery method.	A retrospective chart review	Telehealth for pediatric palliative care.
Wood et al, 2025 [162]	To synthesize how, why, and in which circumstances can videoconferencing be used to successfully meet the emotional support needs of adults receiving palliative care?	Realist review	Video consultations, ie, video calls among adult patients and the bereaved to support emotions by palliative care organizations.
Xu et al, 2023 [163]	To evaluate users’ satisfaction with telehealth palliative care during COVID-19 and to identify facilitators and barriers to telehealth implementation in palliative care during COVID-19.	Systematic mixed studies review	Telehealth services for palliative care using digital communication and information technologies, such as computers, tablets, and mobile phone devices.
Zelko et al, 2022, Austria [164]	To test the effectiveness of a new tool for exchanging information between professionals in palliative care on the primary health care level, with a focus on improving interdisciplinary cooperation and communication.	Mixed methods study	A smartphone tool for exchanging information.
Zheng et al, 2016 [165]	To evaluate caregiver outcomes related to palliative telehealth interventions, with a focus on assessing the effectiveness of telehealth interventions for caregivers of patients in home-based palliative care.	Systematic review	Regular phones for counseling, internet-based interventions, and unspecified telehealth devices were used in the study to provide support and communication to caregivers in the palliative care setting.
Zimmermann et al, 2023, Germany [166]	To identify the needs and concerns of stakeholders regarding the introduction of telehealth in PPHC^v^ and to define recommendations for telehealth in PPHC.	Mixed methods approach with a cross-sectional design	Telehealth interventions including video consultations, data transmission, access to patient records, symptom questionnaires, and communication support were provided to the study participants to improve the quality of care in pediatric palliative home care.

a mHealth: mobile health.

b ISDN: Integrated Service Digital Network.

c SPCT: specialist palliative care consultation team.

d AVA: Angela’s Virtual Assist.

e HRQoL: health-related quality of life.

f TH-SPC: telehealth-assisted home-based specialist palliative care.

g SPC: specialized palliative care.

h GP: general practitioner.

i HCP: health care professional.

j HBPC: home-based palliative care.

k ASyMSp: Advanced Symptom Management System in Palliative Care.

l TM-HBPC: telemedicine home-based palliative care.

m mPCL: mobile-Palliative Care Link.

n RN: registered nurse.

o ICT: information and communication technology.

p NASSS: Non-adoption or Abandonment of technology by individuals and difficulties achieving Scale-up, Spread and Sustainability.

q PPC: pediatric palliative care.

r PSW: personal support worker.

s CNS: clinical nurse specialist.

t EMR: electronic medical record.

u CHF-PC: Concurrent Palliative Care Model for Older Adults with Heart Failure.

v PPHC: Paediatric Palliative Home Care.

Publication dates ranged from 2001 to 2026. Studies from high-income countries were conducted in the Americas (n=45, 35.71%), Europe (n=26, 20.63%), Australasia (n=12, 9.52%), and Asia (n=2, 1.58%). The LMICs represented were from Africa (n=4, 3.17%, representing 22 countries), the Middle East (n=2, 1.58%; ie, Iran and Lebanon), and Asia (n=2, 1.58%; ie, China and India) [9,35,116]. Studies featured qualitative design, including participatory co-design studies and case studies (n=48, 38.09%), mixed method studies (n=25, 19.84%), reviews (n=33, 26.19%), and quantitative studies, which included descriptive (n=14, 11.11%), randomized (n=4, 3.17%), and nonrandomized (n=2, 1.58%) trials.

The majority of the studies met most of the quality criteria (Multimedia Appendix 1). Regarding MMAT scores, the studies scored between 0 and 5 criteria, with a median score of 4.5 (IQR 3-5). For systematic reviews and meta-analyses, the median score was 9 (IQR 8-10) on the Joanna Briggs Institute quality assessment tool.

#### Focus of Included Studies

##### Overview

mHealth emerged as a key strategy for managing symptoms and improving palliative care delivery [52,62,64,70,77]. A recurring theme across studies was the use of technology to facilitate better communication [11,46,50,100,104,125] and care coordination among patients, caregivers, and health care providers [9,59,134]. Several studies concentrated on the psychological impact of digital interventions for both patients and families, highlighting their potential to alleviate distress and improve well-being [45,68,82,116,122,139,162]. End user experiences were critically assessed, with studies delving into patient and caregiver acceptance [93,98,101,106,109], satisfaction, and usability challenges [74,80,99,105,107,121]. Barriers to and facilitators of the wider adoption and integration of telehealth (ie, the use of electronic information and telecommunication technologies to support long-distance health care) into palliative care were also explored, identifying both technical and systemic influencers [41,127,130,132,135,141,150,152,161,167]. The applicability of telehealth in rural [62,117,168] or resource-constrained settings was reported with feasibility and potential to bridge gaps in health care delivery were examined [133,136,143]. Several studies further analyzed the resource utilization and cost-effectiveness of telehealth [49,145,151]. The role of mHealth in pediatric palliative care was also highlighted, as well as the rapid implementation and effectiveness of telehealth interventions during the COVID-19 pandemic [48,51,73,95,126,159].

##### Type of Technology Used

The studies assessed a broad range of electronic devices and described the features for the software used. mHealth devices used were mobile phones or mobile apps (n=23, 18.25%) [9,12,35,45,59,68,74,80,82,99,105,107,116,121,122,127,132-134,136,139,143,145,151], web-based platforms with videoconferencing features (n=15, 11.9%) [48,49,51,56,73,88,95,103,112,114,126,129,140,159,169], direct calls or SMS text messaging (n=4, 3.17%) [9,134,170], and videophones (n=9, 7.14%) [55,72,125,147,160]. The remaining studies included a mix of technologies (n=36, 28.57%) [47,58,61,81,83-85,87,91,92,94,102,108,110,115,118-120,123,124,137,144,146,148,149,153-158,163,165,166,171,172]. The specialized technologies in the studies were an embedded camera pen, remote symptom monitoring wristbands, and e-PC software with integrated camera laptop [51,79].

##### Purpose of the Interventions

mHealth was used for recording pain or other symptoms (n=9, 7.14%) [9,12,61,74,81,138,159,165,166] or remote monitoring (n=15, 11.9%) [11,47,56,63,67,78,86,89,102,111,119,124,126,129,139,147,170], support with medication [11,12], accessing information or serving as an educational resource [10,94,105,123,124,138,159,160,166], and care coordination [45,87,133,139]. Some of the mHealth apps also had built-in alert features [58,74,76]. A few studies engaged diverse stakeholder groups to help develop the interventions [70,81,84,126,127,137,139,140,153,158].

### End Users of mHealth Interventions

The majority of the end users for mHealth interventions were health care professionals [35,72,76,81,84,108,122-124,127,140,164], adult patients [48,56,69,82,87,88,92,96,97,108,113,119-122,134,137,143,156,171], and informal caregivers [9,11,51,59,60,66,82,83,105,112,121-123,132,143,147,153,154,157,163,173]. Only 3 (2.38%) studies directly engaged children or adolescent patients [60,166,169], while other studies broadly explored the use of mHealth to support palliative care for the pediatric population [12,55,61,66,92,94,115,136,173].

### The Contextual Factors That Influence Mechanisms and Outcomes of mHealth

#### Overview

**Figure 3.**
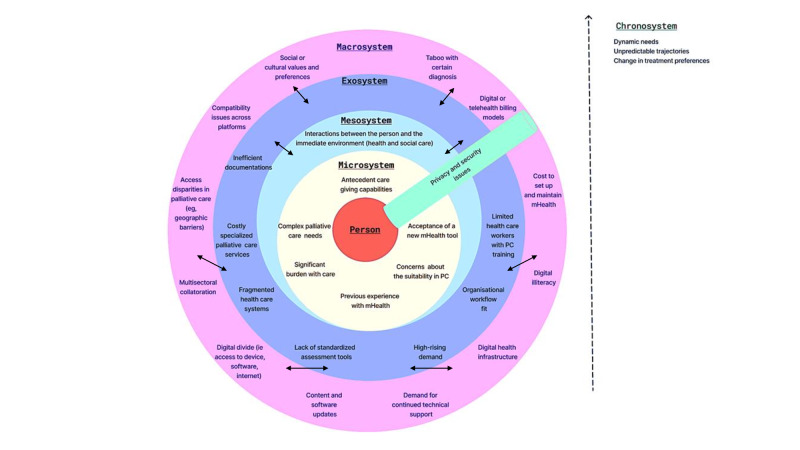
Contextual factors mapped to the domains of the framework of complexity in the palliative care (PC) context demonstrate the relationship between concepts mapped to the interconnected environmental layers of the ecological system model. Key: microsystem (person and immediate social environment and setting), chronosystem (influences of time), mesosystem (interactions between agents in the immediate environment), exosystem (factors within health services or systems), and macrosystem (factors beyond health care) [32]. mHealth: mobile health.

The contextual factors within which mHealth interventions were described using the Framework of Complexity in Palliative Care Context [32] (Figure 3). They encompassed a range of technological, organizational, economical, and social dimensions.

In the microsystem, important concepts were persons’ or mHealth end users’ needs (ie, complex health care and technology-related requirements) [45,63,142]. Individuals’ characteristics were also key, which included limited knowledge about and the skills to support complex palliative care needs [92,97,123,156], the burden experienced by caregivers [9,12,121,128,142], and information gaps [88,134]. Concerning the acceptance of new mHealth tools, contextual concerns reported by end users encompassed their suitability in a palliative care context [95] and the perceived burden of monitoring symptoms for patients and family carers [48]. Furthermore, health care professionals had worries regarding reimbursement with the transition to virtual models of care [49,144]. In addition, having previous experience with similar DHIs and users’ technical competency appeared to influence end users’ confidence in using mHealth [57,76,90,92,99]. As depicted in the mesosystem, existing interpersonal relationships significantly influenced the utility of mHealth interventions. In fragmented health care systems, communication and information exchange challenges were particularly prominent, signifying the relevance of mHealth in such contexts [7,9,11,45,48,51,56,58,59,66,68,69,72,74,85,90,92,95,96,99,102,103,108,116,119,120,122,123,129,137,139,140,142,144,147,148,151,154,157,164,170,171,173,174]. Contextual factors that were identified in the chronosystem comprised the dynamic needs and fluctuating symptoms, which influenced patients’ goals of care and treatment preferences [45,47,51,63,142] and thus impacted their willingness and ability to engage with mHealth technologies. Furthermore, end users, especially older adults, needed time to feel confident and become proficient with digital health tools [51,68].

In the exosystem, there were challenges in terms of a rising demand for palliative care [9,48,85,94,95,108,110,112,115,116,118-120,127,131,136], the lack of standardized assessment tools to appropriately identify patients who require palliative care [35,94,102,105,108,113,126,133,140,143], inefficient documentation and data sharing [87,127,147], and fragmented or siloed health care interventions [60,102,147]. Finally, contextual factors in the macrosystem encompassed disparities in health care access due to geographical barriers, lack of mHealth infrastructure, and inadequate technical support for end users [35,69,71,84,91,116,118,145,174]. Aligning technology with relevant social and cultural values [54,65,67] was reported as key to the successful adoption of mHealth [12,61,88,92,103,105,107,114,121,128,134]. The studies also highlighted the need for legal and organizational frameworks and regulations to ensure data privacy and security [51,94,110,115,119,120,124,126,132,148]. Multisectoral stakeholder engagement was important in the design and implementation stages as buy-in from each stakeholder influences intervention uptake and sustained use [49,99,103,134].

**Figure 4.**
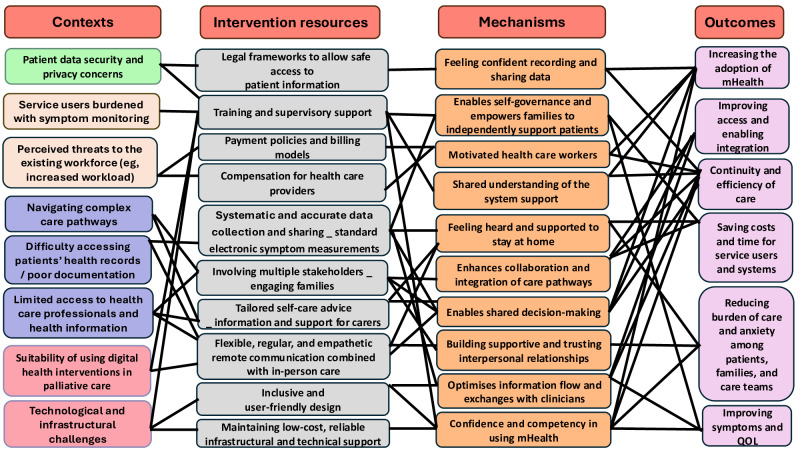
Conceptual model of context-mechanism-outcome configurations (CMOCs) illustrating how contextual factors within a multilevel system and mobile health (mHealth) intervention resources (eg, access to information or data) activate mechanisms (eg, improved capability, confidence, or strengthened interactions), leading to outcomes such as mHealth uptake, individual clinical outcomes, and health system–level impacts. The black lines indicate commonly reported pathways between context, intervention resources, mechanisms, and outcomes. Key: The first column (ie, context) is color coded according to the levels in the framework of complexity in palliative care context. Green: cross-cutting concept across levels in the system, beige: microsystem, blue: exosystem, pink: macrosystem. QOL: quality of life.

The CMOCs for the refined program theory are reported below and summarized in Table 3 and shown in Figure 4.

**Table 3.** List of context-mechanism-outcome configurations (CMOCs).

CMOCs	Description of CMOCs	Evidence summary (studies which contributed to CMOCs)
CMOC 1: Mitigating concerns and perceived threats	1.1 There are uncertainties about the suitability of mHealth^a^ interventions in palliative care contexts in which patients need regular and holistic assessment and support (C), and where monitoring of worsening symptoms could be unsettling (C). Therefore, designing such tools tailored to end users’ needs and capabilities, and providing appropriate orientation on the roles of end users, the frequency and timing of use, and what to expect from the interventions (IR) creates shared understanding (M) and comfort in using them (M) to potentially improve care delivery (O). 1.2 The concerns about patient data security and privacy (C) necessitate that standard legal and ethical frameworks are established and adhered to (ie, to protect patient information) (IR), which creates feelings of security when recording and sharing data (M), thereby increasing the uptake of mHealth (O). 1.3 The perceived threats to health care providers in terms of additional workload with less or no pay (C) demand the recognition of mHealth-supported home-based palliative care as standard payable services (eg, through the introduction of payment policies and billing models) (IR) and appropriate compensation for the health workforce (IR). This acknowledges the efforts and time health care workers spend, motivating them (M) to use mHealth and enabling better integration of mHealth with existing health care systems (O).	[45,47,49,63,102,107,128,149,164] [9,12,35,45,57,60,66,69,70,105,121,132,151] [12,56,61,69,74,97,102,103,120,134,136,147,156,157]
CMOC 2: Minimizing infrastructural and technical barriers for user-friendly and sustained mHealth use	The diverse infrastructural and technological experiences end users have (C) require training and education (IR) and maintaining low-cost, reliable infrastructure (IR) as well as inclusive and user-friendly design alongside offering technical support (IR). This improves confidence (M) and competency in using mHealth interventions (M), thus ensuring usability and wide-scale user penetration (O), which enables the standardization of health care delivery (O).	[35,45,51,55,57-60,63,66,68,69,73,74,84,102,103,108,112,113,115,116,131,136,140,145-147,156,165]
CMOC 3: Engaging multiple stakeholders for better integration	In contexts where patients, families, and care providers are required to navigate complex health care pathways (C), the development of mHealth interventions, which involves multiple stakeholders with diverse roles (IR), creates a shared understanding of the technical and system supports required (M), enhances collaborative relationships (M), and the integration of care pathways (M), which optimize resource utilization (O), potentially saving costs and time for both service users and health care systems (O).	[45,51,55,56,58-61,63,66,69,70,72,84,91,97,120,126,147,149,169,174]
CMOC 4: Streamlined health management information system	In home-based palliative care contexts where accessing patients’ health records and documenting or sharing data is challenging (C), electronic data recording (IR) and standardized digital symptom measurement tools (IR) enable systematic and more accurate data capturing (M) as well as remote monitoring of symptoms (IR). This gives patients and caregivers a sense of comfort (M) and confidence knowing a health care team could be remotely accessible when needed (M), avoiding the inconvenience of unnecessary visits to health care facilities (O), and enabling preemptive management of problems, thus increasing the timeliness of the required support (O).	[9,45,47,51,53,55-60,63,66,70,73,74,76,83-85,92,102,108,110,112,113,115,116,119,121,122,126,127,131,133,136,140,146-148,151,165,166]
CMOC 5: Regular communication and access to care	5.1 In the palliative care context where frequent communication with health care providers is necessary to support complex needs, often with unpredictable trajectories and limited access to health care professionals (C), mHealth provides a flexible and empathetic communication platform (IR), strengthening relationships between service users and service providers (M). This reduces the burden of care and anxiety among patients and families/informal caregivers (O), thus enhancing quality of life (O). 5.2 In contexts where family caregivers are routinely closely involved in care provision (C), combining both in-person and virtual care (IR) and allowing families to join the conversation on equal terms, even remotely, (IR), enables shared decision-making (M) for better management of symptoms (O).	[22,45,50,55,64,68,70,82,98,104,106,107,110,115,125,132-134,137,143,145,146,163,165,167,169,170] [9,10,12,35,45,47,48,51,53,55,56,58-61,63,66,68-70,73,74,83,84,87,88,90-92,94,95,97,99,102,103,105,108,113-116,119,120,122,124,131-133,138,140,142-144,146-149,151,153-156,158,160,164,165,170,174]
CMOC 6: Access adequate information to support symptom	In the palliative care context where adequate information about complex needs and options to support patients and caregivers is required (C), mHealth provides accessible self-care advice tailored to needs and the opportunity for knowledge exchange among health care workers (IR). This empowers end users (ie, patients, families, and junior health care professionals) to independently and effectively support symptoms (M), improving the efficiency of health care delivery (O) and quality of life (O).	[9,12,35,45,47,48,51,53,55,56,59,63,66,68,73,74,80,83,84,88,91,92,94,97,99,102,103,105,108,113,114,116,119,120,122,124,126,131-134,136,138,140,142,143,156,164,166,174]

a mHealth: mobile health.

#### CMOC 1: Mitigating Concerns and Perceived Threats of mHealth

mHealth implementation may be resisted by various end users due to the concerns they may have about the intervention. One of the concerns is the suitability of using DHIs in palliative care for vulnerable patients and inadequately prepared informal caregivers who are already burdened with illness and caretaking responsibilities. This is mitigated by providing appropriate orientation on what to expect from DHIs, the role of end users, and when to use such tools. This in turn creates a shared understanding and tailored use directed to goals of care. Regarding apprehensions about patient data security and privacy, mHealth interventions that are designed adhering to international and local standard legal and ethical protocols and guidelines ensure feelings of security when recording and sharing data and safeguard patient information, which will encourage uptake and sustained use. Health care workers perceived they would experience job security threats and increased workload, for example, with parallel documentation. Furthermore, conventional health care systems were anticipated to take time to adopt mHealth enhanced models, with the need to develop payment protocols or billing systems and lease with the health workforce in the implementation. The shift toward mHealth creates concerns that existing interpersonal interactions would be disrupted. Thus, both service users and providers demanded that health care visits should be a combination of in-person and mHealth. Responding to such concerns will increase the acceptance of digital health technologies for improved care delivery.

#### CMOC 2: Minimizing Technical and Infrastructural Barriers for Sustained mHealth Use

Despite increasing access to mobile phones and increased routine use of mHealth, universal access to mobile devices has not been achieved. Reliability issues and inadequate technical support persist as key challenges in home-based palliative care. Sustained use depends on reliable infrastructure and technology tailored to local capacities. For mHealth to effectively support health care delivery, it should accommodate users’ varying capabilities. National government and institutional support, through policies that promote mHealth adoption, is essential for successful implementation. Moreover, at the design phase, it is important to ensure that the technology is user-friendly and meets the needs of patients, families, and care providers. Health care workers who are confident in their technical skills are more likely to adopt these technologies. Yet, to be inclusive, all end users should receive orientation on the technical and functional aspects of such technologies for standardized use. This will improve self-efficacy and competency, thus ensuring usability with potential for wide-scale user penetration.

#### CMOC 3: Engaging Multiple Stakeholders for Better Integration

In contexts where both service users and care providers are required to navigate complex health care pathways, the development of clinically meaningful mHealth interventions must be designed with relevant multisectoral stakeholders from diverse disciplines. This approach will facilitate the consideration of unique perspectives and address the utility and usability needs and preferences of patients, family caregivers, and health care workers. It is also important to assess the experiences and expectations of various end users, their competencies, and systems constraints to achieve optimal usage for intended outcomes. Furthermore, mHealth interventions must fulfill the standard requirements of health management information systems to ensure effective implementation and integration with routine care. The integration of mHealth in home-based palliative care presents numerous opportunities to enhance care delivery. For example, when mHealth interventions are seamlessly fitting into the standard workflow, health care workers’ capacity to address patients’ needs will increase, which improves the care experience for patients, optimizes resource use, and potentially saves costs and time for both service users and providers.

#### CMOC 4: Streamlined Health Management Information System

mHealth interventions represent efficient care delivery approaches that are well accepted by patients, families, and health care providers who value collaborative care planning and enhanced care coordination to improve patient outcomes. mHealth interventions designed to allow structured collection and reporting of symptoms, such as pain, using digitalized symptom reporting tools by patients or their carers enhance appropriate and timely management. Electronic health recording allows health care providers to closely monitor patients’ conditions even remotely, triaging patients based on severity of symptoms for tailored response, which results in improved quality of care and health care workers’ productivity. Primary care clinicians and generalist palliative care providers could document visits and share data for remote consultations with palliative care specialists, promoting knowledge exchange among health care teams. In addition, mHealth provides the opportunity for palliative care specialists to directly connect with service users (patients and family carers) through real-time audiovisual support. mHealth facilitates home-based care, improving comfort and convenience for patients and families while reducing risks linked to facility-based treatment.

#### CMOC 5: Improved Access to Care

In the management of chronic life-limiting illnesses, direct access to health care providers and regular symptom support are important. mHealth interventions designed to augment home-based palliative care play a pivotal role in facilitating access by surmounting geographical constraints, particularly for underserved populations and communities residing in remote areas. In palliative care context, such tools assist patients with an advanced stage of illness who have difficulties visiting health care facilities or those facing physical barriers to conventional health care services under unique circumstances, as observed during the COVID-19 pandemic. Both health care providers and recipients benefit from reduced burdens associated with logistical challenges and financial expenses associated with traveling to distant health care facilities. Moreover, mHealth fostered close communication between patients and providers and strengthened relationships. Family carers felt more supported, and their capacity to care for their loved ones improved, which reduced the anxiety associated with providing care for patients with serious illnesses. Considering the increasing demand for palliative care and the limited number of palliative care specialists, this is highly relevant to ensuring continuity of care and access to more equitable services.

#### CMOC 6: Access to Adequate Information to Manage Symptoms

The implementation of mHealth interventions serves as a means of disseminating information, thereby augmenting patient engagement in their health care journey. Patients are empowered as their comprehension of illness and its management deepens through personalized self-care advice to manage common symptoms. Empowered patients are more likely to engage in proactive healthy behaviors and adhere to medical advice, which improves symptoms and quality of life. Informal caregivers also benefit from applying mHealth tools in their caregiving roles to enhance their understanding and capabilities to support their loved ones. Health care professionals also use such interventions to access educational resources to assist them in care delivery.

## Discussion

### Principal Findings

This study presents a novel program theory to explore how, why, and under what circumstances mHealth interventions are designed and implemented to support better access to quality home-based palliative care. A program theory (Figure 4) is proposed on the causal explanations and key implementation strategies for successfully integrating mHealth interventions to support home-based palliative care. The important underlying mechanisms highlighted for mHealth intervention to support home-based service are the comfort patients and caregivers had staying at home having access to health care teams when needed; strengthened relationships between service users and providers through regular communication; empowerment of patients, families, and health care providers to independently and effectively manage symptoms through improved knowledge and skills; shared decision-making involving families; having a shared understanding of the technical and system support required; feeling confident and safe with using such technologies; motivation of health care workers through recognition and compensation of their time and effort; and enhanced collaboration and integration with existing health care systems.

Several systematic reviews on mHealth interventions have explored perceptions of end users on the advantages and challenges of their use for home-based palliative care. Our findings are supported by these reviews [3,108], which identified similar individual, interpersonal, organizational, and health systems factors influencing engagement with and effectiveness of mHealth. In palliative care, timely access to services impacts patient comfort and quality of life. mHealth has been identified as a health system–strengthening intervention [3,175]. The impact of mHealth has also been recognized in enhancing operations in pharmaceutical supply chain management systems [176] and in improving care coordination across levels of health care [177]. In studies from sub-Saharan Africa, such benefits were observed in populations living with HIV or AIDS and cancer [178,179]. Collectively, these reviews affirm that mHealth not only enhances individual patient care but also strengthens the broader health care system, making it more accessible, efficient, and resilient. The role of informal caregivers in adapting DHIs in resource-limited settings has also been described previously [180]. Although continuous monitoring and immediate access to information and support can reduce anxiety in patients and their informal carers, this needs to be balanced with the burden of frequent assessment and reporting of symptoms by service users [181,182]. Furthermore, the combination of face-to-face alongside virtual contacts is encouraged [183]; building rapport in an initial in-person visit to develop better therapeutic relationships with subsequent contacts through technology. This prevents the care process from becoming depersonalized and mechanical, which contradicts the principles of person-centeredness in palliative care.

### Strengths and Limitations

The evidence generated in this review drew from a broad global literature to determine how mHealth interventions improve access to and quality of home-based palliative care. It also incorporated perspectives from global and local stakeholders to refine the review focus and enhance the synthesis. The application of the framework of complexity in the palliative care context strengthened the comprehensive exploration of contextual influences of mHealth for home-based palliative care demonstrating interactions of multiple factors in the system. To allow us to generate findings and to provide comprehensive recommendations for home-based palliative care, we did not restrict mHealth use by type of user, the use case, conditions or diagnosis, or type of study. The emphasis of the synthesis was on underpinning mechanisms of action that influence outcomes, which have not been explored in detail in existing literature, nor explicitly stated in the included studies. Therefore, the hypotheses suggested in this study require further testing and refinement.

Contextual variations require intervention strategies that are tailored to the needs or preferences of end users. Most of the included studies in this review were notably from high-income countries; in 7 primary and multisite studies, only 24 countries were represented from LMICs. Therefore, application of these findings from this study needs careful consideration. Despite robust search strategies, only papers published in the English language were considered.

### Recommendations

We have a number of recommendations from this review. To promote the effective integration of mHealth into home-based palliative care, it is essential to advocate for policies that support its adoption and seamless integration within health care systems. This can be achieved through the collaborative co-design of mHealth solutions with multisectoral stakeholders, ensuring that such tools address contextual barriers and align with existing health care infrastructure. The development of user-friendly mHealth interventions, tailored to the specific needs of end users, should be accompanied by comprehensive orientation and technical support to enhance usability and maximize benefits. Additionally, the involvement of informal caregivers, particularly in cultures that emphasize family engagement, is crucial for shared decision-making and the creation of personalized care plans that improve patient outcomes. Investing in reliable infrastructure and support systems is vital to minimize technical failures and ensure continuous care delivery. mHealth should also be leveraged to provide self-care guidance and equip informal caregivers with the necessary skills to support patients at home, thus alleviating pressure from the health care systems. In addition, health care providers must be adequately compensated for the additional time and effort required to operationalize mHealth interventions, which would encourage their active participation in enhancing patient care. Furthermore, the impact of mHealth interventions should be rigorously evaluated to provide evidence that informs policy decisions and promotes best practices that meet the needs of end users. Ongoing research is also necessary to explore the integration processes of mHealth interventions within health care systems, applying standardized frameworks to better understand factors influencing successful implementation.

### Conclusions

To enhance usability and impact of mHealth interventions, it is important to adopt strategies that focus on the important mechanisms identified in this study. These mechanisms include patient and caregiver comfort with home-based care, strengthened provider-user relationships through regular communication, empowerment through better knowledge and skills, family-inclusive decision-making, shared understanding of the technical and system supports, confidence in using technology, motivation of health care workers through recognition and reimbursement, and improved integration with existing health care systems. Further study is needed to validate and evaluate these mechanisms in tailored interventions in LMICs.

## Supplementary material

10.2196/80321 Multimedia Appendix 1 Supplementary materials for the realist review, including the full database search strategy, quality appraisal summaries, and additional details on included studies.

10.2196/80321 Checklist 1 PRISMA checklist.

## Abbreviations

CMOC: context-mechanism-outcome configuration
DHI: digital health intervention
LMIC: low- and middle-income country
mHealth: mobile health
MMAT: Mixed Methods Appraisal Tool
PRISMA: Preferred Reporting Items for Systematic Reviews and Meta-Analyses
WHO: World Health Organization
