# Supplementary material for: Mobile Health in Home-Based Palliative Care: Realist Review of Contextual Factors and Mechanisms Influencing Outcomes
Source: JMIR Mhealth Uhealth. 2026 Aug 14;14:e80321. doi: 10.2196/80321 (PMC13475790; doi:10.2196/80321)
Supplement: Supplementary file 1 [file mhealth-v14-e80321-s001.docx]

**Appendix. Supplementary materials**

**Appendix 1: Glossary of realist terms in the review**

| **Term** | **Explanation** |
| --- | --- |
| Context (C) | The circumstances in which the intervention is implemented, factors that interact, modify, trigger or hinder certain mechanisms and their effectiveness. The context includes participant characteristics, setting, infrastructural, economic, social, political, and cultural norms. |
| Mechanism (M) | How interventions work within specific contexts, a combination of intervention resources (components) offered by mHealth intervention and the end-users’ responses (e.g. reasoning) that are triggered to generate intended outcomes. |
| Outcome (O) | The impact of mechanisms being triggered in certain contexts. For this study, outcomes include clinical and patient-level outcomes (e.g., change in patients’ health), or caregiver level outcomes (e.g., reduced anxiety or satisfaction with care), or health service or systems level outcomes (e.g.) |
| Context–mechanism–outcome configurations (CMOCs) | A programme theory conceptualising the causal pathways between contexts, mechanisms, and outcomes |
| Programme theory | A conceptual framework that lays out what an mHealth programme comprises (intervention components), how and why it is expected to work (user reasoning or response) and under what circumstances (e.g., in terms of contextual factors), and outcomes – intended and un-intended; usually expressed as CMOCs. |

**Appendix 2: List of preliminary context-mechanism-outcome hypotheses (if...then statements after initial searching and data extraction)**

1. If mHealth interventions enhance regular communication, then patients and families had a sense of security and confidence which reduced level of anxiety
2. If mHealth interventions invite families to join conversations on equal terms, then relationships were enriched through shared decision making, which improved satisfaction
3. If mHealth interventions were seamlessly integrated into the clinical workflow, then healthcare providers were more engaged and attentive to patients’ needs due to their increased capacity (time) which improved care experience for patients
4. If mHealth allowed a simple collection of symptoms from patients & flagging of bothersome distresses for clinical review, then patients had appropriate treatment (minimising gaps in timely access to appropriate services)
5. If patients felt mHealth interventions aligned to their priorities or the perceived need for care, then they are more likely to engage in utilising the digital intervention
6. If patient felt burdened with setting up or using the mHealth interventions, then they were discouraged and less likely to sustain use (applies to technical and cost related considerations)
7. If there was a regular utilisation of the mHealth intervention, then the level of command using the technology improved, potentially improving its relevance in healthcare delivery
8. If mHealth intervention was reliable and secure, then patients were more likely to trust it to accessing remote services and virtual care-coordination; to stay home or to be discharged early from hospital-based care
9. If patients and families perceived mHealth interventions as impersonal or difficult to communicate emotions, they are less likely to use them [the need to be culturally relevant]
10. If the service utilising mHealth intervention integrates health and social care, then patients accessed a more responsive care / tailored to their need (needs contextual consideration)
11. If patients self-assess symptoms using mHealth, then their perceptions of agency or control increased (Note that this may also create anxiety so the need to prepare them ahead)
12. If carers had a previous frame of reference of use of digital technology, then they are more confident and ready to adapt the technology (helpful to apply features from familiar platforms such as WhatsApp)
13. If mHealth interventions are building on existing (and tested) functional systems and appropriate infrastructure, then there is a potential reduced need for an in-home physician /specialist’s visit/, which is potentially cost saving to the healthcare system & patients
14. If patients were too ‘unwell’, they are less likely to use the mHealth interventions (particularly if it required frequent measurement of symptoms) [implying the need to tailor frequency of use or to be assisted by families]
15. If care providers had added benefits from in person home visits (e.g., financial compensations only in the face-to-face encounters) then they were gatekeepers in the referral of consumers for tele-hospice interventions
16. If mHealth interventions embodied respectful communication, then patients felt they maintained close connection with their care providers and continued virtual engagement

**Stakeholders engaged in refining the review scope**

The results from the scoping search were discussed with global palliative care researchers and other health care professionals (n=14).

| **Expertise** | **Representing (Country)** |
| --- | --- |
| Family Physician, palliative care researcher | Ghana |
| Family Physician, palliative care instructor | Sudan |
| Internist, palliative care instructor | Uganda, India, UK |
| Family Physician, clinician and academician | South Africa |
| Oncologist, palliative care researcher | Sudan |
| General practitioner / Public health expert | Kenya |
| Family Physician, palliative care provider | Malawi, UK |
| Pediatrician, with palliative care training | Ethiopia |
| Internist / palliative care provider | Ethiopia |
| PhD researcher in Palliative care | Ethiopia |
| Palliative care specialist / General practitioner | Ethiopia |
| Nurse palliative care provider / researcher | Ethiopia |
| Public health expert, health service leader | Ethiopia |
| Public health expert in cancer care/ ethnographer | Ethiopia |

**After stakeholder consultation, the following CMOs were prioritised and re-organized to assist preliminary hypothesis generation, Example codes are listed below.**

| **Theme** | **Context** | **Mechanism** | **Outcome** |
| --- | --- | --- | --- |
| Tailored /flexible communication | If patients are on unstable trajectory of illness (too ‘unwell’ or having burdensome symptoms) | Then a person-centred communication, with tailored frequency and/or with a support from carers or nurses, will facilitate identifying patients’ / family’s needs | Effective management of symptoms (decrease anxiety) and decreased burden of monitoring symptoms, |
| Shared decision making | If patients and families are able to maintain regular communication (through mHealth), | Then there is a shared understanding of care process and clear expectations from treatment | Leading to satisfaction with care |
|  | If mHealth interventions invite families to join conversations on equal terms | Then relationships are enriched through shared decision making and active involvement in care | Leading to improved support for patients and satisfaction with care |
| Fitting in the clinical and health information management workflow /personalised | If Digital platforms enable a blend of in-person and telehealth visits | Then the human-level interaction is maintained | Leading to flexible and accessible care delivery |
| Patients’ agency | If patients can self-assess and manage symptoms having been informed and supported by mHealth, | Then they are empowered and feel in control | Leading to a more independent life |
| Streamlining Care Processes | If remote monitoring and real-time symptom reporting is facilitated | Then accurate data is captured from patients and readily accessed by healthcare providers | Leading to more efficient care delivery |
| Command of mHealth technology | If patients and families have limited ability to use mHealth | Then appropriate training and technical support will improve command of technology | Leading to increased mHealth utilization and acceptability |
| Monitoring of use | If patients, families or health care providers have difficulty in continuing to use mHealth | Then organizational and infrastructural support | Leading to increased engagement and sustainability |

**Appendix 3: Search strategy**

Search strategy and search terms

Search terms

1. **Intervention**

((Mobile App*) OR (Cell Phone) OR (Smartphone) OR (Videoconferencing) OR (Telemedicine) OR (mhealth) OR (m*health) OR (telehealth) OR (mobile phone*))

2. **Population/ Subject**

Patients with palliative care needs ((palliative care) OR (palliat* therapy) OR (end of life care) OR (EOL care) OR (Terminal Care) OR (Palliative Medicine) OR (palliat* treat*))

3. **Setting**

((home-based care) OR (Home Care Services) OR (Home Health Nursing) OR (Community Health Nursing) OR (Community health worker*) OR (health extension worker*) OR (Home care))

Search Combinations

1 AND 2 AND 3

**Electronic database search**

**Embase**

| 1 palliative care.mp. 73347 | 70174 | Advanced |
| --- | --- | --- |

| 2 | exp palliative therapy/ | 150930 |
| --- | --- | --- |
| 3 | palliat* therapy.mp. [mp=title, abstract, heading word, drug trade name, original title, device manufacturer, drug manufacturer, device trade name, keyword heading word, floating subheading word, candidate term word] | 149822 |
| 4 | exp terminal care/ | 88756 |
| 5 | terminal care.mp. [mp=title, abstract, heading word, drug trade name, original title, device manufacturer, drug manufacturer, device trade name, keyword heading word, floating subheading word, candidate term word] | 45357 |
| 6 | end of life care.mp. [mp=title, abstract, heading word, drug trade name, original title, device manufacturer, drug manufacturer, device trade name, keyword heading word, floating subheading word, candidate term word] | 22114 |
| 7 | EOL care.mp. | 2441 |
| 8 | Palliative Medicine.mp. | 4969 |
| 9 | palliat* treat*.mp. [mp=title, abstract, heading word, drug trade name, original title, device manufacturer, drug manufacturer, device trade name, keyword heading word, floating subheading word, candidate term word] | 14070 |
| 10 | 1 or 2 or 3 or 4 or 5 or 6 or 7 or 8 or 9 | 231437 |
| 11 | exp mobile application/ | 27427 |
| 12 | Mobile App*.mp. [mp=title, abstract, heading word, drug trade name, original title, device manufacturer, drug manufacturer, device trade name, keyword heading word, floating subheading word, candidate term word] | 26600 |
| 13 | Cell Phone.mp. | 5272 |
| 14 | exp smartphone/ | 28397 |
| 15 | Smartphone*.mp. [mp=title, abstract, heading word, drug trade name, original title, device manufacturer, drug manufacturer, device trade name, keyword heading word, floating subheading word, candidate term word] | 39262 |
| 16 | exp videoconferencing/ | 9615 |
| 17 | Videoconferen*.mp. | 11340 |
| 18 | exp telemedicine/ | 75306 |
| 19 | Telemedicin*.mp. [mp=title, abstract, heading word, drug trade name, original title, device manufacturer, drug manufacturer, device trade name, keyword heading word, floating subheading word, candidate term word] | 57038 |
| 20 | m*health.mp. | 10149 |
| 21 | exp telehealth/ | 92602 |
| 22 | telehealth.mp. [mp=title, abstract, heading word, drug trade name, original title, device manufacturer, drug manufacturer, device trade name, keyword heading word, floating subheading word, candidate term word] | 29178 |
| 23 | mobile phone*.mp. [mp=title, abstract, heading word, drug trade name, original title, device manufacturer, drug manufacturer, device trade name, keyword heading word, floating subheading word, candidate term word] | 30055 |
| 24 | exp mobile phone/ | 50267 |
| 25 | 11 or 12 or 13 or 14 or 15 or 16 or 17 or 18 or 19 or 20 or 21 or 22 or 23 or 24 | 181814 |
| 26 | exp home care/ | 93328 |
| 27 | home*based care.mp. [mp=title, abstract, heading word, drug trade name, original title, device manufacturer, drug manufacturer, device trade name, keyword heading word, floating subheading word, candidate term word] | 38 |
| 28 | home-based care.mp. [mp=title, abstract, heading word, drug trade name, original title, device manufacturer, drug manufacturer, device trade name, keyword heading word, floating subheading word, candidate term word] | 1541 |
| 29 | Home Care Services.mp. | 2901 |
| 30 | Home Health Nursing.mp. | 245 |
| 31 | Home Health Nursing.mp. | 245 |
| 32 | exp community health nursing/ | 27338 |
| 33 | Community Health Nursing.mp. [mp=title, abstract, heading word, drug trade name, original title, device manufacturer, drug manufacturer, device trade name, keyword heading word, floating subheading word, candidate term word] | 27679 |
| 34 | Community health worker*.mp. | 8309 |
| 35 | health extension worker*.mp. | 635 |
| 36 | exp home care/ | 93328 |
| 37 | Home care.mp. [mp=title, abstract, heading word, drug trade name, original title, device manufacturer, drug manufacturer, device trade name, keyword heading word, floating subheading word, candidate term word] | 82340 |
| 38 | 26 or 27 or 28 or 29 or 30 or 31 or 32 or 33 or 34 or 35 or 36 or 37 | 132525 |
| 39 | 10 and 25 and 38 | 339 |

**MEDLINE**

| 1 | palliative care.mp. [mp=title, book title, abstract, original title, name of substance word, subject heading word, floating sub-heading word, keyword heading word, organism supplementary concept word, protocol supplementary concept word, rare disease supplementary concept word, unique identifier, synonyms, population supplementary concept word, anatomy supplementary concept word] | 81035 |  |
| --- | --- | --- | --- |
| 2 | exp Palliative Care/ | 64341 |  |
| 3 | palliat* therapy.mp. [mp=title, book title, abstract, original title, name of substance word, subject heading word, floating sub-heading word, keyword heading word, organism supplementary concept word, protocol supplementary concept word, rare disease supplementary concept word, unique identifier, synonyms, population supplementary concept word, anatomy supplementary concept word] | 2254 |  |
| 4 | end of life care.mp. [mp=title, book title, abstract, original title, name of substance word, subject heading word, floating sub-heading word, keyword heading word, organism supplementary concept word, protocol supplementary concept word, rare disease supplementary concept word, unique identifier, synonyms, population supplementary concept word, anatomy supplementary concept word] | 14802 |  |
| 5 | EOL care.mp. | 1418 |  |
| 6 | exp Terminal Care/ | 57877 |  |
| 7 | Terminal Care.mp. [mp=title, book title, abstract, original title, name of substance word, subject heading word, floating sub-heading word, keyword heading word, organism supplementary concept word, protocol supplementary concept word, rare disease supplementary concept word, unique identifier, synonyms, population supplementary concept word, anatomy supplementary concept word] | 33581 |  |
| 8 | Palliative Medicine.mp. [mp=title, book title, abstract, original title, name of substance word, subject heading word, floating sub-heading word, keyword heading word, organism supplementary concept word, protocol supplementary concept word, rare disease supplementary concept word, unique identifier, synonyms, population supplementary concept word, anatomy supplementary concept word] | 2983 |  |
| 9 | exp Palliative Medicine/ | 559 |  |
| 10 | palliat* treat*.mp. | 8551 |  |
| 11 | 1 or 2 or 3 or 4 or 5 or 6 or 7 or 8 or 9 or 10 | 133005 |  |
| 12 | exp Mobile Applications/ | 12223 |  |
| 13 | mobile app*.mp. [mp=title, book title, abstract, original title, name of substance word, subject heading word, floating sub-heading word, keyword heading word, organism supplementary concept word, protocol supplementary concept word, rare disease supplementary concept word, unique identifier, synonyms, population supplementary concept word, anatomy supplementary concept word] | 19030 |  |
| 14 | exp Cell Phone/ | 23293 |  |
| 15 | Cell Phone.mp. [mp=title, book title, abstract, original title, name of substance word, subject heading word, floating sub-heading word, keyword heading word, organism supplementary concept word, protocol supplementary concept word, rare disease supplementary concept word, unique identifier, synonyms, population supplementary concept word, anatomy supplementary concept word] | 12027 |  |
| 16 | exp Smartphone/ | 9725 |  |
| 17 | Smartphone.mp. [mp=title, book title, abstract, original title, name of substance word, subject heading word, floating sub-heading word, keyword heading word, organism supplementary concept word, protocol supplementary concept word, rare disease supplementary concept word, unique identifier, synonyms, population supplementary concept word, anatomy supplementary concept word] | 23734 |  |
| 18 | exp Videoconferencing/ | 2777 |  |
| 19 | Videoconferencing.mp. [mp=title, book title, abstract, original title, name of substance word, subject heading word, floating sub-heading word, keyword heading word, organism supplementary concept word, protocol supplementary concept word, rare disease supplementary concept word, unique identifier, synonyms, population supplementary concept word, anatomy supplementary concept word] | 4497 |  |
| 20 | Videoconferenc*.mp. [mp=title, book title, abstract, original title, name of substance word, subject heading word, floating sub-heading word, keyword heading word, organism supplementary concept word, protocol supplementary concept word, rare disease supplementary concept word, unique identifier, synonyms, population supplementary concept word, anatomy supplementary concept word] | 5390 |  |
| 21 | exp Telemedicine/ | 46709 |  |
| 22 | telemedicine.mp. [mp=title, book title, abstract, original title, name of substance word, subject heading word, floating sub-heading word, keyword heading word, organism supplementary concept word, protocol supplementary concept word, rare disease supplementary concept word, unique identifier, synonyms, population supplementary concept word, anatomy supplementary concept word] | 51743 |  |
| 23 | mhealth.mp. [mp=title, book title, abstract, original title, name of substance word, subject heading word, floating sub-heading word, keyword heading word, organism supplementary concept word, protocol supplementary concept word, rare disease supplementary concept word, unique identifier, synonyms, population supplementary concept word, anatomy supplementary concept word] | 10217 |  |
| 24 | m*health.mp. | 10537 |  |
| 25 | telehealth.mp. [mp=title, book title, abstract, original title, name of substance word, subject heading word, floating sub-heading word, keyword heading word, organism supplementary concept word, protocol supplementary concept word, rare disease supplementary concept word, unique identifier, synonyms, population supplementary concept word, anatomy supplementary concept word] | 15430 |  |
| 26 | mobile phone*.mp. [mp=title, book title, abstract, original title, name of substance word, subject heading word, floating sub-heading word, keyword heading word, organism supplementary concept word, protocol supplementary concept word, rare disease supplementary concept word, unique identifier, synonyms, population supplementary concept word, anatomy supplementary concept word] | 15061 |  |
| 27 | 12 or 13 or 14 or 15 or 16 or 17 or 18 or 19 or 20 or 21 or 22 or 23 or 24 or 25 or 26 | 117558 |  |
| 28 | home-based care.mp. | 1270 |  |
| 29 | exp Home Care Services/ | 51276 |  |
| 30 | Home Health Nursing.mp. [mp=title, book title, abstract, original title, name of substance word, subject heading word, floating sub-heading word, keyword heading word, organism supplementary concept word, protocol supplementary concept word, rare disease supplementary concept word, unique identifier, synonyms, population supplementary concept word, anatomy supplementary concept word] | 569 |  |
| 31 | Community Health Nursing.mp. [mp=title, book title, abstract, original title, name of substance word, subject heading word, floating sub-heading word, keyword heading word, organism supplementary concept word, protocol supplementary concept word, rare disease supplementary concept word, unique identifier, synonyms, population supplementary concept word, anatomy supplementary concept word] | 20130 |  |
| 32 | Community Health Nursing/ | 19772 |  |
| 33 | Community health worker*.mp. [mp=title, book title, abstract, original title, name of substance word, subject heading word, floating sub-heading word, keyword heading word, organism supplementary concept word, protocol supplementary concept word, rare disease supplementary concept word, unique identifier, synonyms, population supplementary concept word, anatomy supplementary concept word] | 10543 |  |
| 34 | health extension worker*.mp. [mp=title, book title, abstract, original title, name of substance word, subject heading word, floating sub-heading word, keyword heading word, organism supplementary concept word, protocol supplementary concept word, rare disease supplementary concept word, unique identifier, synonyms, population supplementary concept word, anatomy supplementary concept word] | 533 |  |
| 35 | Home care.mp. | 56383 |  |
| 36 | Home Care Service*.mp. [mp=title, book title, abstract, original title, name of substance word, subject heading word, floating sub-heading word, keyword heading word, organism supplementary concept word, protocol supplementary concept word, rare disease supplementary concept word, unique identifier, synonyms, population supplementary concept word, anatomy supplementary concept word] | 39608 |  |
| 37 | 28 or 29 or 30 or 32 or 33 or 34 or 35 or 36 | 94173 |  |
| 38 | 11 and 27 and 37 | 133 |  |

**PsycInfo**

| 1 | | exp Palliative Care/ | | |
| --- | --- | --- | --- | --- |
| 2 | palliative care.mp. [mp=title, abstract, heading word, table of contents, key concepts, original title, tests & measures, mesh word] | |  | 18944 |
| 3 | palliat* therapy.mp. [mp=title, abstract, heading word, table of contents, key concepts, original title, tests & measures, mesh word] | |  | 36 |
| 4 | end of life care.mp. | |  | 6074 |
| 5 | EOL care.mp. | |  | 590 |
| 6 | Terminal Care.mp. | |  | 5056 |
| 7 | Palliative Medicine.mp. | |  | 1065 |
| 8 | palliat* treat*.mp. [mp=title, abstract, heading word, table of contents, key concepts, original title, tests & measures, mesh word] | |  | 251 |
| 9 | 1 or 2 or 3 or 4 or 5 or 6 or 7 or 8 | |  | 23455 |
| 10 | exp Mobile Applications/ | |  | 2873 |
| 11 | Mobile App*.mp. [mp=title, abstract, heading word, table of contents, key concepts, original title, tests & measures, mesh word] | |  | 5167 |
| 12 | exp Mobile Phones/ | |  | 8108 |
| 13 | Cell Phone*.mp. [mp=title, abstract, heading word, table of contents, key concepts, original title, tests & measures, mesh word] | |  | 3286 |
| 14 | exp Smartphones/ | |  | 3487 |
| 15 | Smartphone*.mp. [mp=title, abstract, heading word, table of contents, key concepts, original title, tests & measures, mesh word] | |  | 8285 |
| 16 | exp Videoconferencing/ | |  | 999 |
| 17 | Videoconferenc*.mp. [mp=title, abstract, heading word, table of contents, key concepts, original title, tests & measures, mesh word] | |  | 2642 |
| 18 | exp Telemedicine/ | |  | 14638 |
| 19 | Telemedicine*.mp. [mp=title, abstract, heading word, table of contents, key concepts, original title, tests & measures, mesh word] | |  | 11238 |
| 20 | mhealth.mp. | |  | 2171 |
| 21 | m*health.mp. [mp=title, abstract, heading word, table of contents, key concepts, original title, tests & measures, mesh word] | |  | 2237 |
| 22 | telehealth.mp. | |  | 4556 |
| 23 | mobile phone*.mp. [mp=title, abstract, heading word, table of contents, key concepts, original title, tests & measures, mesh word] | |  | 8396 |
| 24 | 10 or 11 or 12 or 13 or 14 or 15 or 16 or 17 or 18 or 19 or 20 or 21 or 22 or 23 | |  | 36664 |
| 25 | exp Home Care/ | |  | 7826 |
| 26 | home-based care.mp. [mp=title, abstract, heading word, table of contents, key concepts, original title, tests & measures, mesh word] | |  | 516 |
| 27 | exp Health Care Services/ | |  | 304808 |
| 28 | Home Care Service*.mp. [mp=title, abstract, heading word, table of contents, key concepts, original title, tests & measures, mesh word] | |  | 4299 |
| 29 | Home Health Nursing.mp. | |  | 74 |
| 30 | Community Health Nursing.mp. | |  | 1338 |
| 31 | Community health worker*.mp. [mp=title, abstract, heading word, table of contents, key concepts, original title, tests & measures, mesh word] | |  | 1849 |
| 32 | Home care.mp. [mp=title, abstract, heading word, table of contents, key concepts, original title, tests & measures, mesh word] | |  | 15648 |
| 33 | 25 or 26 or 27 or 28 or 29 or 30 or 31 or 32 | |  | 316423 |
| 34 | 9 and 24 and 33 | |  | 169 |

**Global health**

| 1 | | palliative care/ 1303 | |
| --- | --- | --- | --- |
| 2 | Palliative Care.mp. [mp=abstract, title, original title, heading words, cabicodes words] | | 2287 |
| 3 | palliat* therapy.mp. [mp=abstract, title, original title, heading words, cabicodes words] | | 71 |
| 4 | end of life care.mp. | | 554 |
| 5 | EOL care.mp. | | 57 |
| 6 | Terminal Care.mp. | | 122 |
| 7 | Palliative Medicine.mp. | | 80 |
| 8 | palliat* treat*.mp. [mp=abstract, title, original title, heading words, cabicodes words] | | 300 |
| 9 | 1 or 2 or 3 or 4 or 5 or 6 or 7 or 8 | | 2947 |
| 10 | mobile applications/ | | 729 |
| 11 | Mobile App*.mp. | | 1812 |
| 12 | mobile telephones/ | | 5234 |
| 13 | Cell Phone*.mp. [mp=abstract, title, original title, heading words, cabicodes words] | | 872 |
| 14 | Smartphone.mp. | | 2854 |
| 15 | Videoconferenc*.mp. [mp=abstract, title, original title, heading words, cabicodes words] | | 377 |
| 16 | telemedicine/ | | 3761 |
| 17 | Telemedicine*.mp. [mp=abstract, title, original title, heading words, cabicodes words] | | 4759 |
| 18 | mHealth/ | | 313 |
| 19 | mhealth.mp. [mp=abstract, title, original title, heading words, cabicodes words] | | 1339 |
| 20 | m*health.mp. [mp=abstract, title, original title, heading words, cabicodes words] | | 1401 |
| 21 | telehealth.mp. | | 1696 |
| 22 | mobile phone*.mp. | | 3272 |
| 23 | 10 or 11 or 12 or 13 or 14 or 15 or 16 or 17 or 18 or 19 or 20 or 21 or 22 | | 14788 |
| 24 | home care.sh. | | 2782 |
| 25 | home-based care.mp. [mp=abstract, title, original title, heading words, cabicodes words] | | 408 |
| 26 | Home Care Service*.mp. [mp=abstract, title, original title, heading words, cabicodes words] | | 293 |
| 27 | Home Health Nursing.mp. | | 2 |
| 28 | Community Health Nursing.mp. | | 56 |
| 29 | Community health worker*.mp. [mp=abstract, title, original title, heading words, cabicodes words] | | 4999 |
| 30 | community health workers/ | | 1956 |
| 31 | health extension workers/ | | 27 |
| 32 | health extension worker*.mp. [mp=abstract, title, original title, heading words, cabicodes words] | | 481 |
| 33 | 24 or 25 or 26 or 27 or 28 or 29 or 30 or 31 or 32 | | 8475 |
| 34 | 9 and 23 and 33 | | 4 |

**Web of science**

| 1 | (TS=(palliative care) OR TS=(terminal care) OR TS=(palliat* therapy) OR TS=(end of life care) OR TS=(EOL care) OR TS=(Palliative Medicine) OR TS=(palliat* treat*) OR AK=(palliative care) OR AK=(terminal care) OR AK=(palliat* therapy) OR AK=(end of life care) OR AK=(EOL care) OR AK=(Palliative Medicine) OR AK=(palliat* treat*)) and Preprint Citation Index (Exclude – Database) | [244,673](https://www.webofscience.com/wos/alldb/summary/a7d3c237-5500-4d39-8336-94b9b8ee1d54-ce5f60ca/relevance/1) |
| --- | --- | --- |
| 2 | (TS=(Mobile Applications) OR TS=(mobile app) OR TS=(Cell Phone) OR TS=(Smartphone) OR TS=(Videoconferencing) OR TS=(Telemedicine) OR TS=(mhealth) OR TS=(m*health) OR TS=(telehealth) OR TS=(mobile phone) OR AK=(Mobile Applications) OR AK=(mobile app) OR AK=(Cell Phone) OR AK=(Smartphone) OR AK=(Videoconferencing) OR AK=(Telemedicine) OR AK=(mhealth) OR AK=(m*health) OR AK=(telehealth) OR AK=(mobile phone)) and Preprint Citation Index (Exclude – Database) | [510,493](https://www.webofscience.com/wos/alldb/summary/de9d6407-01d0-45b6-b9d7-bfca90158f64-ce5fca31/relevance/1) |
| 3 | TS=(home-based care) OR TS=(Home Care Services) OR TS=(Home Health Nursing) OR TS=(Community Health Nursing) OR TS=(Community health worker) OR TS=(health extension workers) OR TS=(Home care) OR TS=(Home Care Service) OR AK=(home-based care) OR AK=(Home Care Services) OR AK=(Home Health Nursing) OR AK=(Community Health Nursing) OR AK=(Community health worker) OR AK=(health extension workers) OR AK=(Home care) OR AK=(Home Care Service)) and Preprint Citation Index (Exclude – Database) | [432,183](https://www.webofscience.com/wos/alldb/summary/d5b7d83d-b528-4e8a-a716-6dcef203bb81-ce602f0e/relevance/1) |
| 4 | #1 AND #2 AND #3 and Preprint Citation Index (Exclude – Database) | [1,144](https://www.webofscience.com/wos/alldb/summary/8a18ab5c-0ca3-4935-ae62-967980d825da-ce603665/relevance/1) |

**CINAHL**

| **S6** | (MH "Home Health Care") OR (MH "Community Health Nursing") |  |
| --- | --- | --- |
| **S5** | TI ( "Home care" OR "Home Care Service*" OR "home-based care" OR "Home Health Nursing" OR "community health nursing") OR AB ( "Home care" OR "Home Care Service*" OR "home-based care" OR "Home Health Nursing" OR "community health nursing" ) |  |
| **S4** | (MH "Cellular Phone") OR (MH "Telehealth") OR (MH "Mobile Applications") OR (MH "Smartphone") OR "telemedicine" |  |
| **S3** | TI ( “cell phone” OR smartphone OR “mobile phone” OR m-Health OR "mobile app*" OR telehealth OR telemedicine ) OR AB ( “cell phone” OR smartphone OR “mobile phone” OR m-Health OR "mobile app*" OR telehealth OR telemedicine ) |  |
| **S2** | (MH "Palliative Care") OR (MH "Palliative Medicine") OR (MH "Hospice and Palliative Nursing") |  |
| **S1** | TI ( ( "palliat* care" OR "palliat* treat*" OR "palliat* medicine" OR "palliat* therap*" OR "Terminal Care" OR "End of life" OR "EOL" OR ) ) OR AB ( ( "palliat* care" OR "palliat* treat*" OR "palliat* medicine" OR "palliat* therap*" OR "Terminal Care" OR "End of life" OR "EOL" OR ) ) | **87** |

**SCOPUS**

| **1** | (TITLE-ABS-KEY("palliat* care") OR TITLE-ABS-KEY("palliat* therap*") OR TITLE-ABS-KEY("end of life care") OR TITLE-ABS-KEY("EOL care") OR TITLE-ABS-KEY("Terminal Care") OR TITLE-ABS-KEY("Palliat* medicine") |  |
| --- | --- | --- |
| **2** | TITLE-ABS-KEY("Mobile App*") OR TITLE-ABS-KEY("Cell Phone") OR TITLE-ABS-KEY("telemedicine") OR TITLE-ABS-KEY(mhealth) OR TITLE-ABS-KEY(smartphone) |  |
| **3** | TITLE-ABS-KEY("Home Care Services") OR TITLE-ABS-KEY("Home care") OR TITLE-ABS-KEY("home-based care") OR TITLE-ABS-KEY("Home Health Nursing") OR TITLE-ABS-KEY("Community Health Nursing")) |  |
| **4** | 1 AND #2 AND #3 | **17** |

**Cochrane Database of Systematic Reviews**

| **1** | palliative care OR terminal care OR palliat* therapy OR end of life care OR EOL care OR Palliative Medicine OR palliat* treat* |  |
| --- | --- | --- |
| **2** | Mobile Applications OR mobile app OR Cell Phone OR Smartphone OR Videoconferencing OR Telemedicine OR mhealth OR m*health OR telehealth OR mobile phone |  |
| **3** | home-based care OR Home Care Services OR Home Health Nursing OR Community Health Nursing OR Community health worker OR health extension workers OR Home care OR Home Care Service |  |
| **4** | #1 AND #2 AND #3 | **2** |

**Global Index Medicus (GIM)**

| **1** | (tw:(“palliative care” OR “terminal care” OR “palliative therapy” OR “end of life care” OR “EOL care” OR “Palliative Medicine” OR “palliative treatment”)) | 1592 |
| --- | --- | --- |
| **2** | (tw:(“Mobile Applications” OR “mobile app” OR “Cell Phone” OR Smartphone OR Videoconferencing OR Telemedicine OR mhealth OR telehealth OR “mobile phone” )) | 1265 |
| **3** | (tw:(“home-based care” OR “Home Care Services” OR “Home Health Nursing” OR “Community Health Nursing” OR “Community health worker” OR “health extension workers” OR “Home care” OR “Home Care Service” )) | 16 |
| **4** | (tw:(“palliative care” OR “terminal care” OR “palliative therapy” OR “end of life care” OR “EOL care” OR “Palliative Medicine” OR “palliative treatment”)) OR (tw:(“Mobile Applications” OR “mobile app” OR “Cell Phone” OR Smartphone OR Videoconferencing OR Telemedicine OR mhealth OR telehealth OR “mobile phone” )) AND (tw:(“home-based care” OR “Home Care Services” OR “Home Health Nursing” OR “Community Health Nursing” OR “Community health worker” OR “health extension workers” OR “Home care” OR “Home Care Service” )) | **0** |

**Appendix 4. Eligibility criteria**

| **Inclusion criteria** | **Exclusion criteria** |
| --- | --- |
| **Types of study**  Peer reviewed publications of any study design that evaluates mHealth interventions (medical and public health practice supported by mobile and wireless devices, such as voice and short message services and applications [1] in the context of home-based (a model of care that provides comprehensive medical and supportive services within the comfort of patients’ homes) [2,3] palliative care (which supports patients with life-limiting illness and their families, through ‘the prevention and relief of suffering through early identification, impeccable assessment, and treatment of pain and other problems; physical, psychosocial, and spiritual’ [4]. Studies that describe the programme theories, i.e. hypothesized explanations of how the mHealth interventions support palliative care work were considered. | Unpublished such as conference abstracts, and master’s theses |
| **Participants/population**  Patients, both adult and paediatric, with life-threatening and life-limiting illnesses (i.e. medical conditions that have a high probability of causing death which often require immediate and intensive medical intervention to prevent mortality e.g., advanced cancer, severe heart disease, and serious infections. Life-limiting illnesses are chronic conditions for which there is no reasonable hope of cure and that will ultimately result in the patient's death. These illnesses significantly impair quality of life and daily functioning, necessitating ongoing management to alleviate symptoms and provide supportive care e.g., advanced dementia, progressive neurological diseases, and certain chronic respiratory conditions [5], with no age limitations, their formal and informal caregivers (*who are unpaid, informal providers of one or more physical, social, practical, and emotional tasks. In terms of their relationship to the patient, they may be a friend, partner, ex-partner, sibling, parent, child, or other blood or non-blood relative* [6], health care providers, community health workers, and health extension workers. | Patients solely on acute care, or curative treatment; caregivers and health care providers with no palliative care role. |
| **Intervention**  mHealth defined as medical and public health practice supported by mobile and wireless devices, such as voice and short message services and applications. For this review, included the practice of palliative care supported by text message, voice or video call, or application-based or web-based technology (using social media or purposely designed apps) using mobile phones or tablets. [1,7,8]. | Interventions that do not primarily utilize mHealth. |
| **Context**  Home-based palliative as a service delivered to seriously ill patients in the setting that a patient calls “home,” i.e. a private residence [9]. | Facility-based services (primary health care facilities, hospitals), nursing homes, long-term care homes, and hospices (outpatient, day-care and in-patient) |

**Appendix 5. Evidence from papers supporting context–mechanism–outcome configurations**

CMOC 1: Mitigating concerns and perceived threats

The participants expressed concern about where the data were stored and who had access to them. They requested clarification of the policy of data use in the app[…] It is not feasible however to provide a valid data policy during the developmental stage. The data collection for the development stage is in-line with the GDPR.

The participants questioned the use of an external forum because the app provides a link to the Cancer Research UK online forum. They suggested the provision of explanations about the benefits that they might obtain from using it. […] This recommendation was addressed by adding an induction screen before linking to the external forum to highlight the potential benefit from sharing thoughts with others in the same situations and to provide hints for a better user experience.

[1]

Of equal importance is the need to explore issues related to the privacy, safety and security of data and its transfer. [2]

The remaining two studies took different approaches using a multimedia Internet-based intervention to support pain management and a patient portal to promote individual’s end of life wishes. [3].

Other themes included ways to improve, benefits and de-tractors, privacy and confidentiality. [10]

There are also privacy issues with the remote possibility of a web based video call being intercepted. For these reasons a Polycom teleconferencing phone is used for the sharing of audio. By using standard telephone lines for audio we are able to ensure the confidentiality of the call for both the clinicians and the family. [14 Bradford A telemedicine]

One potential participant refused the use after reading the questions of eIPOS in the introductory visit, pointing out that he was not willing to be ‘rudely’ directed regularly to his situation by the questionnaire.

Health care professionals’ concerns that patients’ condition might not allow independent ePROM use were mirrored in the difficult recruitment process for the patient-interviews. In the focus groups, a repeatedly proposed solution to this challenge was to involve relatives in the use of eIPOS. [15]

Other patients tended only to use eIPOS on ‘good days’, everything else would mean too much burden in situations where they are confronted with, for example, intense pain, severe vomiting, or psychological problems. Patients in a more stable condition and/or with non-oncological diseases preferred a lower frequency of eIPOS use, because they felt that noteworthy changes were not as frequent. [15]

Studies have also shown that older adults with cognitive impairment seem to prefer standard and less complicated health technology due to different levels of complexity affected by several habit aspects. [17]

Privacy issues. Patient did not want family to overhear conversation so he had to leave house and conduct phone call. [18]

In India, there was concern that home visits would be replaced by the app, and also concerns regarding data privacy. [31]

The presence of a caregiver was also related to appropriateness of video-visits. Visits with clients who had caregivers present were less likely to be suitable for videophone visits, which probably reflect situations where clients were in the more advanced stages of decline or needed additional care. […] Disease trajectory may be an important factor in determining if and when the videophone may be useful. Clients diagnosed with cancer were more likely to be suitable for video-visits. However, clients with chronic illness may be expected to see a gradual decline in health status, with an undefined change from routine to palliative care. [33]

One of the participants (telephone line only) said that he would consider the iPad to be an invasion of privacy and stated a preference for the telephone line. [43]

Cancer survivors indicated no concerns about the app, and one care partner mentioned a concern about keeping medical information private, [55]

Of the 80 participants, 31 (39%) expressed concerns about using an app, with security and usability being commonly mentioned. Despite caregivers expressing security concerns, most individuals expressing security concerns stated they would use an app regardless. [64]

Threat: Ethical issues related with data protection [65].

Systems-level factors: such as insurance reimbursement and out-of- state coverage restrictions [69].

While privacy and security are important considerations for technology enabled solutions they may also limit flexibility in delivery of community services if the patient’s personal IT resources are not able to be networked to the health service. [81]

CMOC 2: Minimizing infrastructural and technical barriers for sustained and user-friendly mHealth use

All participants found the app and the portal easy to use and understand. There were minimal problems navigating between screens and finding a way to accomplish an aim. [1]

(i) patients not having access to phones […] (ii) mobile network access, with unreliable networks and limited internet in some countries. [2]

technology literacy, which can be seen as a prerequisite for the utility of mHealth. [17]

Support must also exist on a day-to-day level, with adequate resources to ensure ongoing success. [20]

This study aimed to design a mobile phone application (app) to enable or improve communication between family caregivers, community caregivers, and palliative care teams; to evaluate its acceptability, processes, and mechanisms of action; and to propose refinements. […] training in use of the app should be expanded with longer-term support and mentorship for end users. [31]

Demographic variables such as age and gender were not associated with the appropriateness of video-visits. [33]

TH is considered one of the most important alternative approaches to balancing conficts between demand for and the supply of home-based palliative care. [29]

the technical implementation regarding, for example, the design of the patients’ questionnaire or the display of the ePROMs in the teams documentation system, plays a crucial role for acceptance. As the participating teams did not use standardised PROM assessment in daily care routine before, it might be possible that identified challenges relate generally to the routine use of standardised measurement of patient reported outcomes and not explicitly to its electronic realisation.

Clinicians noted that the elderly, those in rural areas, and those with less-resourced backgrounds have greater difficulty using telehealth. Perceived barriers were largely patient-based factors, including technological literacy, internet and device availability, and patient preferences. Clinicians agreed that several organizational factors facilitated telehealth EIPC delivery, including technological infrastructure, training, and support from study coordinators [69].

CMOC 3: Engaging multiple stakeholders for better integration

This study underpins the importance of including stakeholders as experts of their life-worlds in the development of new health technologies.[15]

The success of the telehospice service was correlated with the support offered by senior executives in the hospice organization. Without this support, the service failed; with it, the service was successful. [20]

Careful codesign and appraisal of the app and intervention from multiple stakeholder viewpoints are crucial to maximize acceptance and clinical benefit. […] engagement and codesign with IT professionals, caregivers, and palliative care teams should continue to drive the refinement and delivery of this app. [31]

“using telehealth to ‘come to [patients]’ adds a layer of convenience that makes it easier for them to get on board with visits” [69]

Patient and family expectations must be clarified at enrollment. Families enrolled in the DSP often seek this care because they have very challenging circumstances and are struggling to manage. The care that can be provided using telemedicine is limited and this can lead to disappointment if expectations are not made clear [70].

CMOC 4: Streamlined health management information system

After patients have submitted data via the app, HCPs and researchers can access and view the patient’s data via the system portal. [1]

Major themes addressed in these articles included communication of patient reported outcomes (PROs), increased patient and/or caregiver usage with technology, and the inclusion of caregivers in the information exchange process between patients and clinicians. [3].

Schoppee et al. used the PAINReportIt® on Wi-Fi enabled tablets to report pain in real time to health care providers, indicating pain location, intensity, quality, pattern, and other pain-related information measured by the McGill Pain Questionnaire (MPQ). [7]

Most of them would prefer to receive new eIPOS information in the morning when daily planning is organised. Nevertheless, health care professionals emphasised that time resources are scarce then and reading out a comprehensive list of patient-reported symptom burden and problems in eIPOS might be challenging. They suggested a distinction between relevant symptoms and others that do not require immediate reaction from the team. [15]

Participants in the second FG considered the telehealth self-monitoring tools as a significant means of providing them with meaningful and timely, patient-reported, clinical data that were not currently available to them. [19]

Characteristic of all these applications is their connectivity to a webpage that makes all the information collected via the patients’ mobile devices available to health professionals. [22]

Specific questions are how and when the data should be received and interpreted by health-care professionals; which health-care professionals should receive and interpret the data and decide on the appropriate response; which health-care professionals should communicate a response to which type of caregiver which outcomes are targeted for improvement; and which people-centered outcomes should drive further refinement of the app. [31]

CMOC 5: Regular communication and access to care

These suggestions confirmed the importance of providing users with feedback regarding their interaction with the app. This had already been documented in the requirements list of the app, but it was not feasible to integrate it into the prototype’s design. [1]

Kenya: The patient or relative can call to consult, to inform that they are coming for services, to get clarification. For follow-up especially if a patient is very sick to offer support to the family, just to be there but via the phone. [2]

Participants in these articles reported high levels of satisfaction, were comfortable with using technology, felt they could have meaningful conversations of sensitive topics through technology platforms, and that the technology-enhanced their quality of life with no detriment to the patient-clinician relationship. [3]

Patient A: “I am very much looking forward to using the system. It is my honor to be one of the first patients for the system. It is good to have access every day since patients want to see medical staff in cases when they have a problem.” [4]

Data recorded via the website by patients and families could be shared with the clinical team to identify, describe, prioritise and monitor the issues that most impact on quality of life. [5]

The e-palliative system is a means to overcome the lack of communication between the doctor and the patient when the home care team does not have a doctor. [6]

Three studies claim that the use of technological tools in home palliative care has a positive impact on communication between clinicians and patients, promoting a stronger connection between the 2 parties than in-person visits. [7]

The video-phone allows more personalized communication between family members and members of the paediatric palliative care team. Conference-style communication is facilitated by the video-phones, which improves the effectiveness and efficiency of communication. [8]

Videotelephony has provided a promising and economically attractive option for supporting adult palliative care in the US and as a useful means of extending specialist palliative care to remote areas in Australia. [9]

“I could definitely see if I was having a problem and I felt like I needed to call [the APRN] or wanted to see the doctor, this would definitely raise my comfort level to be able to show them something as opposed to just tell them what's going on ... [10]

RELIEF will provide real-time communication of patient symptoms to clinical staff for proactive prevention, monitoring, and intervention, while reducing patient stress in knowing their healthcare provider is monitoring their symptoms. [11]

The ability to bridge in a family member who lives far away for a videoconference or allow them to follow their loved one’s symptom assessments and communication with providers via TapCloud gave family members an increased knowledge about what is occurring and provided a heightened level of involvement and a sense of comfort. [12]

The clinicians and family members are able to use the Internet for video communication while maintaining the security, privacy and quality of audio through a standard telephone call. [13]

Patients and caregivers report overwhelmingly positive experiences with the telehealth program as demonstrated by the qualitative survey results. Patients and caregivers like that TapCloud is specifically tailored to their conditions, symptoms, and medications and allows their voice to be captured in a meaningful way. [12]

Participating health care professionals discussed challenges how to react on eIPOS-reported values. One crucial aspect was how to deal with the potential expectation of patients to receive a prompt reaction[…]Some focus group participants were unsure whether the team would be able to respond appropriately to all aspects returned in the eIPOS because of limited team resources, for example, psychological or spiritual support. [15]

(Benefits) To address this, in 2009 a PPCS was established in Queensland, Australia, which routinely uses telehealth to communicate with families and primary care clinicians who are dispersed across the state.[14]

The questionnaire asks for the last three days. Which symptoms in the last three days. If he has severe pain on day one, how can I tell if day one with severe pain has already been treated. [15]

Telehospice questionnaire asks -based hospice care to communicate with hospice staff and family members, while remaining compliant with privacy laws. [16]

 However, what distinguishes mHealth from other digital health technologies is its ability to provide mobile self-care, which enables people to monitor their health data without a clinician’s assistance and to communicate and interact remotely. [17]

All participating PC clinicians received training on “Webside Manner”(i.e., how to communicate effectively and maintain rap-port and human connection during virtual video visits). [18]

This FG (focus group) participant articulates telehealth monitoring tools as a mechanism to place responsibility on patients and families not only for identifying but also for acting on their symptoms. [19]

The meetings were augmented by weekly email messages sent to coordinators of the project. The content and frequency of the routine email messages and meetings symbolized the commitment from project coordinators and produced a supportive telehospice culture. [20]

Following his discharge home, the community nurses reinforced the family training and supported them in his care. The family preferred not to have a home health aide and soon asked that the nursing visits be decreased in frequency. However, they remained dependent on daily communication, and reinforcement and support from the palliative care team at MSKCC. [21]

Although the apps facilitate communication with health professionals and generate alerts for health services, due precautions are necessary to ensure that patients realize that the application is not a substitute for the normal care health professionals provide but complements it. [22]

It is anticipated that these videoconference engagements will strengthen the communication and overall relationships between staff, patient, and patient family and have been noted to increase patients’ and their family members’ overall satisfaction in relation to the quality of care they are receiving. [25]

The studies demonstrated that videoconferencing and other technologies proved effective for clinician-to-clinician communications, as well as interprofessional patient and family meetings. [28]

TH provides an opportunity for efficient communication, continuity of care, and meets the growing demands of care services across remote areas with limited resources. [29]

The app enabled patient-reported outcome measurement in routine practice in three LMICs that can facilitate patient assessment, staff awareness of patient concerns, communication, and possibly also improved patient outcomes. [31]

They highlighted multiple aspects of the service that were patient centred including: more patients seen, more responsive (quicker to be seen), patients not needing to travel, inclusion of family/carer. [32]

Both studies found that the technology enhanced communication between home care clients and healthcare providers, and concluded that the videophones were user-friendly and generally well accepted. [33]

using telehealth … adds a layer of convenience that makes it easier for them to get on board with visits … the ability to maintain continuity of care … opportunity for enhanced rapport early in the disease course and over time … the possibility for joint visits with other specialists and family members, increased patient engagement in palliative care, and improved continuity and longitudinal care. Clinicians reported that patients with travel considerations, such as longer commute times and/or limited transportation options, benefitted the most from using telehealth to increase access to telehealth … telehealth delivery has increased access for patients who refuse to come to clinic for additional appointments or who may be hesitant about meeting with palliative care. Patients with health-related considerations (e.g., difficulty with mobility or frailty), limited support, and comfort with technology were also felt to have improved access due to telehealth … [69].

They felt it was safest when delivered by an experienced provider, they had access to a reliable caregiver [70]

Efficient palliative care requires good communication between all experts who care for the patient, which makes patients and their relatives feel safe in their home environment and allows patients to stay at home despite the progression of their disease… improving interdisciplinary collaboration between individual providers for patients receiving palliative care [91].

CMO 6: Access adequate information to support symptom

as a stand-alone application to support pain self-management assisting patients to monitor pain and reflect on what could help to ease pain or to avoid triggering pain.

The participants suggested sending a notification to patients when their pain level shows a consistent or increasing layout or when they fail to log pain details for a few days. [1]

Cameroon: To encourage, give information for a particular problem, give or repeat directives.

Mauritania: Online learning in the office and downloading of articles for offline educational use.

[2]

communication research is in exchanging information, enabling patient self-management, and responding to emotions…Such communication topics are crucial to cancer patients with advanced disease and should not be overlooked. Technology-based interventions maybe able to address more than 1 area of Epstein and Street’s communication framework categories — importantly managing patient care more holistically. [3].

In contrast, our results suggest that the use of ePROMs could also serve as a facilitator as it allows patients to better understand and express the perception of their illness. [15]

Using mobile technology to seek knowledge about health information on the Internet and apply the knowledge gained to address or solve a health problem. […] can be a resource to facilitate independent living. [17]

To be more interesting and accessible to patients and caregivers, information is delivered through different modalities, including modules, videos, avatars, and cultural integration resources, and also includes “emergency call” functions for severe symptoms. [22]

Appreciation of accessibility of information “…they valued the information, guidance, and emotional support” [70]

**Appendix 6. Studies included in the review**

| Author Year, Country | **Aim** | **Study design** | **Sample** | **Intervention** | **Relevance** | **Rigour** |
| --- | --- | --- | --- | --- | --- | --- |
| Abahussin 2019, UK[10] | To design and develop a mobile health-based pain recording system for cancer patients in a home setting to support effective pain management. | User-centred design (UCD) (multiple staged including usability testing | Cancer patients and palliative care professionals | A mHealth-based pain recording system for adult cancer patients in home settings, a mobile app for patients to log pain details, a web-based application for healthcare professionals' (and researchers' use) | M | H |
| Allsop 2019, 21 countries in Africa[11] | To assess the current use of mHealth in palliative care service delivery in the African region, identify barriers to mHealth use, and determine provider priorities for research development. | Mixed methods descriptive design using an online survey | nurses, physicians, administrators, clinical officers, volunteers | Mobile phone use | H | H |
| Al-Mondhiry 2022, USA[12] | To use community-partnered participatory research to co-design and pretest a mobile app that focuses on palliative care priorities of clinicians and patients with advanced cancer. | Co-design workshops | clinicians and patients | Mobile app i.e., wellness journal to track patient-reported symptoms, goals, and medication use; information on self-management of symptoms; community resources; and patient and caregiver testimonial videos. | H | M |
| Ansari 2022, USA[13] | To identify technology-based communication strategies to improve health outcomes in individuals with advanced cancer, apply Epstein and Street's framework to assess the impact of technology-based communication, and identify gaps in research and care for cancer patients with advanced disease. | Integrative review | 39 documents | Assessment of mixed technologies e.g., Health information technology, electronic health reporting, data sharing between care teams, and telehealth programs | H | H |
| Aoki 2006, Japan[14] | To evaluate the implementation of tele-palliative care in a rural community for patients with terminal cancer who prefer home-based palliative care | Mixed-methods study | patients and their families | Tele-palliative care using a Polycom View station with ISDN line for communication, including telephone calls, conversations among healthcare providers and patients, and recording of vital signs. | L |  |
| Archer 2021[15] | To explore the impact of digital health interventions on the psychological outcomes of patients and families receiving paediatric palliative care. | Systematic Review | 3 documents | Telehealth consultations via video link and telephone, telehealth intervention via Zoom videoconferencing/face time, and the use of a bespoke website (My-Quality.net) to collect data on patient reported outcomes. | M | H |
| Baldwin 2012, USA[16] | To determine what organizational factors might account for differences in the utilization of telehospice. | Survey | investigation was based on the 2007 National Home and Hospice Care Survey (NHHCS), among sample of US home health and hospice agencies, and patients. | Telehospice (i.e., video conferencing) utilised by hospice agency staff and patients. | M | L |
| Balasubramanian 2022, India[17] | To assess the level of satisfaction of patients receiving e-palliative homecare services and to evaluate the feasibility of e-palliative care in providing palliative care to advanced cancer patients at their homes, particularly focusing on a context of limited resource | Prospective observational study - Quantitative descriptive | patients, caregivers, nurses, doctors, trainee social workers | Implementation of e-palliative care through a technological interface, where a homecare team led by a PC trained nurse visits patients with a camera integrated laptop and e-PC software application installed, allowing remote communication with a PC physician in the hospital. | M | M |
| Bandini 2025, USA[18] | To examine current perspectives from interdisciplinary providers on the use of telehealth and inperson care for outpatient palliative care among underserved patients. | Qualitative study | outpatient palliative care providers and clinic staff (n = 17) from one health system | Telehealth use among staff serving underserved poulation | H | H |
| Basile 2023 [19] | To map the literature on the use of technology by terminally ill older adults receiving palliative care at home, identify the technology systems in use, understand the impact of technology on communication between palliative care professionals and patients, and explore the strengths or weaknesses perceived by patients regarding the use of technology. | Scoping review | 14 studies | Tablets, smartphones, computers, videoconferencing devices, a pen with an embedded camera, and devices for remote symptom monitoring such as smartphones and wristbands. | H | M |
| Bauer 2026 Denmark[20] | To develop programme theory for a family-focused model of telepalliative care for adult patients and their families. | Qualitative study - workshops with key stakeholders, participant observations and ethnographic interviews | patients with palliative care needs, family caregivers, SPCT members, allied healthcare personnel, community care nurses, IT personnel and healthcare managers from both community and tertiary care settings. | Telepalliative consultation perceptions among patients with palliative care needs, family caregivers, SPCT members, allied healthcare personnel, community care nurses, IT personnel and healthcare managers from both community and tertiary care settings. | H | H |
| Bensink 2004, Australia[21] | To design and test an Internet-based videophone for paediatric palliative care services, focusing on assessing the feasibility and benefits of using a low-bandwidth Internet-based videophone for telemedicine in home settings. | Feasibility or pilot study - qualitative exploration | families, parents, palliative care clinical nurse consultant (CNC) | Video-phone- use of low-bandwidth, internet-based videophone for supporting paediatric palliative care in the home, with clinical consultations conducted twice each week | L | H |
| Bensink 2009, Australia[22] | To investigate the acceptability of providing videotelephone-based support to families receiving paediatric oncology-related palliative care and to explore the integration of videotelephone support from the time of diagnosis through outpatient care and support. | Randomized controlled trial (RCT) - an acceptability trial | patients | Access to a videotelephone service in addition to the usual 24-hour on-call telephone support service, provided by specialist nurses for patient assessment, monitoring, family education, communication, and counselling. | M | L |
| Bethel 2021, USA[23] | To evaluate technology system usability and CPATH implementation effectiveness in seriously ill older adults and their caregivers. | Qualitative study | seriously ill older adults and their caregivers | Comprehensive Patient Assessment for Using Telehealth at Home intervention to meet remotely and assess patients in their own home | H | H |
| Bhargava 2021, Canada[24] | To develop and evaluate a remote symptom self-reporting app called RELIEF for community patients with palliative care needs, assessing its feasibility and acceptability for remote monitoring of patients through regular symptom self-reporting, with a focus on utilizing technology to improve symptom management and care delivery. | Co-design- pilot feasibility study - quantitative | patients | Patients self-reported symptoms daily using RELIEF, received over-the-phone assessments and interventions based on alerts, and some received support for expected death in the home. | L | M |
| Bonsignore 2018, USA[25] | To describe and evaluate a telehealth palliative care program using the TapCloud application and videoconferencing in rural areas, focusing on feasibility, usability, symptom burden, and hospice transitions. | mixed-methods study | patients, caregivers, and providers | TapCloud application for remote patient monitoring, including symptom and medication management, messaging, and photo upload, as well as videoconferencing for real-time interactions. | M | H |
| Bradford 2014, Australia[26] | To compare the costs of paediatric palliative care medical consultations conducted via the Home Telehealth Program (HTP) with in-person consultations, specifically focusing on cost minimization. | A cost minimisation analysis | families, caregivers | Palliative care consultations through the Home Telehealth Program (HTP) at the Royal Children's Hospital in Brisbane, involving video consultations in the home | H | H |
| Bradford 2014, B, Australia[27] | To investigate the potential of telemedicine for the delivery of specialist support in the home or local community for children with life-limiting conditions requiring palliative care. | Qualitative study | children, families, specialist health professionals | Real-time audio-visual communication links between the specialist paediatric palliative care team and families caring for a child receiving palliative care at home, facilitated by personal computers, web cameras, and the internet. Some families were provided with loan computers and prepaid wireless mobile internet connections. | H | H |
| Brännström 2024, Sweden[28] | To describe healthcare professionals’ experiences of video consultations in palliative care in community homecare and nursing homes in rural areas. | Qualitative study | healthcare professionals’ | Video consultations to access specialist palliative care service. | M | M |
| Burner-Fritsch 2023, Germany[29] | To identify and explore challenges for the development of electronic patient-reported outcome measures as standardised assessment in specialised palliative home care, focusing on patient, health care professional, and organizational/structural levels. | Qualitative study | Patients and professionals from five German palliative home care teams. | Patients used the electronic version of the Integrated Palliative Care Outcome Scale (eIPOS) on their own web-enabled devices, completing it 1-2 times a week for at least 2 weeks, with support from the researcher if necessary. | M | H |
| Caetano 2024, Portugal[30] | To explore whether caregivers feel prepared to provide informal palliative home care, their experiences, and the usefulness of telehealth in managing daily activities. | Qualitative study | caregivers | Telehealth to support informal caregivers. | M | M |
| Calton 2020, USA[31] | To characterize the experience of patients with serious illness and their caregivers receiving palliative care (PC) by telemedicine. | Mixed-methods telephone survey | Patients and caregivers | Telemedicine acceptability and comfort amongst patients and caregivers. | M | H |
| Cameron 2021, USA[32] | To examine the comfort and emotional support that caregivers of home hospice patients derive from using electronic tablets for tele hospice. | Mixed methods study | A total of 667 caregivers of home hospice patients participated | Caregivers received electronic tablets with the AVA application for tele hospice participation, were given a presurvey, shown how to use the tablets, completed surveys after setting up AVA in the home, and received a postsurvey two weeks after the death of the loved one. | H | H |
| Carey 2023, Ethiopia[33] | To co-design, develop and evaluate a mobile phone based remote monitoring system for use by palliative care patients in Ethiopia. | Co-design and qualitative study | healthcare workers, palliative care patients, family carers & software-developers. | Remote monitoring system where patients and families record their symptoms and access information to self-support their needs. | H | H |
| Christiansen 2020, Sweden [34] | To describe perceptions of mHealth and its impact on health-related quality of life (HRQoL) among older adults with cognitive impairment. | qualitative study with phenomenographic approach | older adults with cognitive impairment | SMART4MD, an mHealth intervention to improve the HRQoL of older adults with mild cognitive impairment and their informal caregivers. | H | H |
| Chua 2022, USA[35] | To describe trends in in-person and video visit PC delivery challenges before and during the COVID-19 pandemic in the U.S. | Quantitative non-randomized | patients | Early integrated telehealth palliative care (via video visit) or in-person palliative care, with at least monthly visits. Initial visit in person within four weeks of enrolment but could be conducted via video during the COVID-19 pandemic. Subsequent follow-up visits every four weeks, either via video or in person. | M | H |
| Collier 2016, Australia[36] | To explore clinicians' perspectives and experiences of telehealth, perspectives about benefits and challenges for patients, families, and clinicians in using telehealth applications, and identified enablers and barriers for the utilization of telehealth in community palliative care. | Qualitative study | clinicians, nurses, medical officers | The intervention included self-report assessment tools, remote activity monitoring, video-based conferences, and virtual case conferences. | H | H |
| Collier 2025, Australia and Aotearoa, New Zealand (NZ)[37] | To explore the complexities of telehealth and palliative care with a focus on access and equity. | Qualitative study | clinicians | Telehealth for extending reach of palliative care in Underserved groups. | H | H |
| Cook 2001, USA[38] | To analyse the successes and challenges of implementing tele hospice care focusing on the role of senior executives' support, quality of care, and provider acceptance. | observational study - interviews | patients | Implementation of tele hospice services using telemedicine technology, including training of potential tele hospice users and providing care remotely via videophones over ordinary telephone lines to patients in rural and urban communities in Kansas and Michigan for at least a year. | L | H |
| Coyle 2002, USA[39] | To explore the effectiveness and benefits of using audio-visual communication as a complementary tool in caring for complex palliative care patients who are isolated at home due to their medical conditions, aiming to enhance communication and support for both the patients and their caregivers. | Case Report - a three-month trial | a complex palliative care patient | Trial of a commercially available audiovisual system, specifically a videophone (TeleEye 324 TM, LeadTek Research, Inc., Fremont, CA), placed in the patient's home using standard telephone wires for a three-month trial period. | M | H |
| Cruz 2023 [40] | To map the available knowledge regarding the use of applications for mobile devices to support adult patients in palliative care at home, specifically focusing on mHealth applications under study/development in the home care transition process for palliative care patients. | Review | 24 documents : (patients, caregivers, professionals) | Participants received the mobile application intervention for monitoring symptoms, communication with healthcare professionals, and potentially receiving alerts and educational materials related to palliative care. The intervention was compared with controls in some studies, and outcomes were assessed in terms of changes in daily functioning, self-efficacy, and quality of life. | H | H |
| Davis 2015, USA[41] | To describe the Tele-Caring intervention and its outcomes, focusing on proactively identifying and addressing the needs of home hospice patients and caregivers through a systematic phone-based care service. | a quality improvement (QI) initiative | home hospice patients, TeleCaring Specialists, TeleCaring Nurses | TeleCaring consisted of daily proactive phone calls to patients and caregivers by specialists and nurses, made up to twice daily based on the patient or caregiver's preferred frequency and timing, providing up to 14 additional staff interactions each week. The calls aimed to address questions or concerns related to care and ensure supply needs were met. | H | H |
| Dickman 2020, USA[42] | To explore provider perspectives regarding the utility of mHealth in palliative care. | Qualitative study | Providers from multiple disciplines working in palliative care settings at an academic medical center | mHealth perception of use amongs providers from multiple disciplines working in palliative care settings | H | H |
| Disalvo 2021[43] | To identify digital health technologies evaluated for supporting timely assessment and management of people with palliative care needs at home and their carers, and to summarize the evidence base through a meta-review. | Meta-review | 16 documents | Remote Patient Monitoring (RPM) interventions included monitoring of biometric data or symptoms, with alerts triggered to clinicians when patient scores were outside acceptable thresholds. One trial involved patients with chronic obstructive pulmonary disease, showing no difference in outcomes, while another trial with patients with advanced lung cancer or lung metastases showed a reduction in postoperative symptoms. | H | H |
| Dismore 2025, UK[44] | To understand the experiences of patients, carers and specialist palliative care professionals to receive/ deliver video consultations in a rural setting. | Qualitative study | Patients, their informal carers (family/friend) and members of the community specialist palliative care team | video consultation perception of use to access specialist palliative care. | M | H |
| Dolan 2021[45] | To assess the safety and effectiveness of virtual care in end of life and palliative care. | Review | Thirty-three peer reviewed articles | Virtual care i.e., video consultation, mobile apps, videos, websites, telephone support, email and alert messages. | M | L |
| Domínguez 2026, Ecuador[46] | To evaluate the technology usability and implementation feasibility of the NEST (Non-intrusive Devices for Telemedicine) system, a low-cost, smartwatch-based pain monitoring solution for palliative cancer care co-designed with health care staff from a cancer hospital in Ecuador. | Usability and Feasibility Study | patients | Wearable-Based Pain Monitoring System | H | H |
| Doolittle 2019, USA[47] | To explore the feasibility and effectiveness of utilizing a TeleHospice approach in rural hospice care, focusing on enhancing patient-and family-centered, timely care through the use of low-cost, secure mobile tablet technology like iPads, while also aiming to advance hospice care through innovative and cost-effective approaches. | Product design- Community-Engaged Model | Patients, hospice personnel (nurses, doctors), family members of patients, super user rural hospice nurses, bereavement coordinator from HSI, HSI staff members | Utilization of mobile tablets for videoconferencing encounters in rural and frontier hospice care settings, involvement of an interprofessional team, and planned deployment of iPads to patients and families during the clinical pilot phase. | H | M |
| Evering 2022, Netherlands[48] | To explore the intention to use video communication by healthcare providers in interprofessional terminal care and predictors therein. | Cross-sectional study - survey | healthcare professionals and volunteers working in first-line healthcare | The intervention was an online survey to explore the intention to use video communication in terminal care. | L | H |
| Gonzalez 2023, USA [49] | To evaluate the perception of usability of a tablet device among palliative care patients and informal caregivers, with the goal of improving tablet usability for home health management. | cross-sectional pilot study | ten patients and twelve caregivers from palliative care practice within an academic medical centre in the mid-Atlantic region | A home health tablet device | M | M |
| Gordon 2022[50] | To explore strategies for increasing access to palliative care among individuals living in remote/rural communities through the utilization of telehealth applications | Rapid review | 18 documents | The interventions included videoconferencing, telephone-based interventions, web-based platforms, after-hours phone support service, remote patient monitoring, and management, web-based videoconference platforms, and 24/7 phone consult services. | L | M |
| Guo 2023, China [51] | To examine telehealth readiness and its related factors among Chinese palliative care specialist nurses, with a focus on improving the implementation of telehealth services in palliative care. | Cross-sectional study | nurses | Not specific (telehealth readiness) - Assessment using the Chinese version of Telehealth Readiness Assessment Tools (TRAT-C) and the Innovative Self-Efficacy Scale (ISES-C). | L | H |
| Hancock 2019[52] | To describe the current use of telehealth in palliative care in the UK, evaluate telehealth initiatives against a digital service standard, and explore whether telehealth results in a reduction in emergency care access. | Systematic review | 30 documents | The intervention(s) was home telemonitoring using telephone or computer software to record clinical symptoms or signs from the patient's home. Patients were required to input specific data regarding symptoms and physical parameters, with some studies including telephone support. | L | H |
| Harding 2021, India, Uganda, and Zimbabwe [53] | To design and evaluate a mobile phone application to improve communication between family caregivers, community caregivers, and palliative care teams, assess its acceptability and mechanisms of action, and propose refinements based on stakeholder feedback. The study focuses on developing a mobile app for real-time patient outcome data reporting and monitoring in palliative care settings in low-and middle-income countries. | mixed-methods study | family caregivers, community caregivers, doctors, nurses, allied health professionals | The intervention involved the use of a mobile phone application for family or community caregivers to report nonurgent patient and family outcomes on a dashboard accessible by the clinical palliative care team in three LMICs. The app aimed to facilitate patient assessment, improve staff awareness of patient concerns, enhance communication, and potentially improve patient outcomes. | H | H |
| Haroen 2025[54] | to investigate the benefits of integrating telehealth into community-based palliative care for people living with terminal illness and their caregivers. Methods: A systematic search of studies was conducted using M | Review | Seven studies | Telehealth i.e., telephone or video consultations and phone calls. | H | H |
| Haydon 2021, Australia[55] | To explore the costs, service activity, and staff experiences resulting from the introduction of telehealth in a community palliative care service in Queensland, Australia. | Mixed-methods study | staff members involved with the community palliative care service, including medical, nursing, and administrative personnel | Service activity for in-person, home visits, telephone, and telehealth (video consultations)- Implementation of telehealth in the community palliative care service to provide palliation services through video consultations in patients' homes or local medical services. | L | H |
| Hebert 2007, Canada [56] | To determine the potential for using videophones in palliative home care and to explore factors that could inform the eligibility criteria for video-visits. | Retrospective chart review | Three hundred and fifty-four visits - palliative home care health records of deceased clients | Palliative home care visits that could have been carried out using videophones, tele hospice care as a support for family caregivers, testing the usability of a videophone in palliative home care, and determining the potential for home telehealth in palliative care. | L | H |
| HENNEMANN-KRAUSE 2015, Brazil[57] | to examine telemedicine as a form of home and additional support for traditional outpatient care as a way to remotely monitor and manage the symptoms of patients with advanced cancer. | prospective, longitudinal, qualitative, descriptive, and case series study | patients | Web conferences to remotely monitor and manage the symptoms | H | H |
| Hoek 2017, Netherlands[58] | To determine whether weekly teleconsultations from a hospital-based specialist palliative care consultation team (SPCT) improved patient-experienced symptom burden compared to "care as usual" in home-dwelling patients with advanced cancer. | Randomized clinical trial | patients | weekly teleconsultations for a period of 13 weeks in addition to their usual care. First, a teleconsultation device was installed at the pa tient’s home. Patients who had not visited the SPCT before were evaluated at the outpatient clinic or during a home visit by one of the SPCT members | M | H |
| Holland 2014, USA[59] | To determine the resources needed to use the system and the quality of the audio and visual components to conduct virtual visits between a clinician at an academic centre and community-dwelling adults living in rural locations. | Mixed-methods field study design | PC patients. However, healthy, community-dwelling older adults (aged 55+ years) were participants in this proof-of-concept study | Virtual visits using a 3G-enabled Apple iPad, cellular phone data service, and a web-based video conference service. | M | H |
| Holmen 2020[60] | To identify and review the use of eHealth to communicate and support home-based paediatric palliative care | Convergent, systematic mixed methods review | 7 documents. | Interventions received by the study participants included videoconferences, the use of an online tool for monitoring quality of life, and the use of iPads for videoconferencing, among others. | L | H |
| Hutchinson 2022, USA[61] | To explore the acceptability, feasibility, and emotional responsiveness of tele-PC. | A mixed-methods formative study | clinicians | Telemedicine perception of use i.e., acceptability, feasibility, and emotional responsiveness of tele-palliative care | M | M |
| Jess 2019[62] | To review and synthesize current evidence regarding the use of video consultations in both general and specialized palliative care to various patient groups, addressing the advantages, disadvantages, barriers, and facilitators of implementing video consultations in palliative care from multiple perspectives. | Systematic integrative review | 39 documents | Use of video consultations in specialized palliative care settings | L | M |
| Jiang_2023, Australia[63] | To assess the feasibility of a telehealth-assisted home-based specialist palliative care (TH-SPC) model for an Australian rural setting, focusing on integrating telehealth technology to provide specialist palliative care to rural communities efficiently and improve health-care metrics. | Prospective mixed-methods pilot study | patients, caregivers | Video consultations with metropolitan-located specialist palliative-care physicians alongside standard care. | L | H |
| Johnston 2011[64] | To explore the use of telehealth in relation to palliative care in the UK. | Review |  | Telehealth use for older people | M | M |
| Johnston 2012, UK[65] | To evaluate the current use of Telehealth applications within palliative care across Scotland, assess how these applications are perceived by patients, carers, and professionals, and improve the use and provision of Telehealth applications within palliative care in the future. | Qualitative Evaluation design | patients, carers | Telehealth activity amongst the key stakeholders centred on the use of videoconferencing equipment for networking, consultations, multidisciplinary meetings and for education and training | L | H |
| Johnston 2014[66] | To scope information available from published and unpublished research on the current state of palliative home-based technology, practitioner-focused perspectives, patient-focused perspectives, quality of life, and the implications for clinical practice. | Review |  | Home-based technology perspectives of use among clinicians and patients and implication on practice | H | H |
| Karen 2019, Denmark[67] | To explore the advantages and disadvantages of using video consultations in specialized palliative care, focusing on the experiences of healthcare professionals and their perspectives on video consultations. | Qualitative study | Healthcare professionals, patients, social worker | Patients referred to specialized palliative care received an initial home visit by an SPC team head physician and an SPC team nurse. They were primarily in contact with the SPC team nurse by phone and had approximately one weekly video consultation using a tablet provided by the SPC team. | H | H |
| Kawashima 2025[68] | To evaluate the effectiveness of and implementation requirements for telehealth in palliative care patients with advanced cancer. | Review |  | Telehealth i.e., videoconferencing, telephone, or text-based communication | H | H |
| Keenan 2021, UK[69] | to explore potential divergence and convergence in relation to health care professionals’ and patients’ acceptability of the use of telehealth within palliative care provision through the lens of Self-Determination Theory. | Qualitative study | health care professionals and patients | Telehealth perceptions among health care professionals and patients | H | H |
| Kidd 2010[70] | To review telehealth applications used in palliative care settings in the UK, identify who is using telehealth, for what purpose it is being used, and to explore if telehealth use is increasing within the clinical setting | Systematic review | 21 documents | The interventions - for health professionals, patients, carers, nurses, GPs- include telehealth services such as out-of-hours telephone support, advice services, videoconferencing, and training and education for healthcare staff. | M | M |
| Kluger 2023, USA[71] | To determine whether PC training for neurologists and remote access to a PC team can improve outcomes in patients with Parkinson disease and related disorders (PDRD) in community settings, focusing on patient quality of life, caregiver burden, and other patient-centered outcomes. | This pragmatic, stepped-wedge comparative effectiveness trial | patients, caregivers, community neurologists, clinicians | PC education for community neurologists and provision of team-based PC resources via telehealth, including consultations from an academic center's PC team. | M | M |
| Kwon 2026, Republic of Korea[72] | To (1) develop the Hospice@Home system, a digital in-home hospice care solution; (2) explore preliminary indications of usability and feasibility among patients with terminal cancer and their caregivers; and (3) identify challenges for future implementation. | Development and Usability Pilot Study | terminal cancer and their caregivers; | Digital in-home hospice care solution for patients with terminal cancer and their caregivers. | H | H |
| Laura 2019, UK[73] | To evaluate the Gold Line telehealth initiative in providing support to individuals at home in the last year of life, focusing on patient and carer experiences and the extension of palliative care services to those not known to specialist palliative care. | Mixed-methods evaluation | patients and carers. | Access to the Gold Line service, training for healthcare teams, use of the Gold Standards Framework, access to electronic health records, recording call outcomes, and participation in interviews. | H | H |
| Lee 2022, Ireland[74] | To understand patient experience | Mixed quantitative and qualitative study. | patients | Video consultation – perception of use among patients | M | M |
| Lind 2008, Sweden[75] | To explore and describe palliative home care patients' experiences of assessing their pain by using a pain diary together with digital pen and mobile Internet technology. | A case study with cross-case content analysis. | patients, spouses, caregivers, physicians, next-of-kin | Introduction of pain management clinical guidelines, pain treatment for patients with at least moderate pain, and follow-up of pain treatment using the IT system developed. | M | H |
| Lundereng 2023[76] | To systematically map published studies on HCPs’ experiences and perspectives on the use of telehealth in HBPC. | Scoping review | 29 documents from 28 studies | Video-based technology for teleconsultations among patients, families, and healthcare professionals, delivered by hospital-based HCPs, hospice workers, and home care professionals either independently or in collaboration with hospital-based staff. | L | M |
| Ma 2025[77] | To provide a comprehensive understanding of the roles of nurses and the multilevel facilitators and barriers to implementing nurse-delivered telehealth in home-based palliative care, which could inform future policy development, research, and clinical practice | Review |  | Telehealth for home-based palliative care delivered by nurses | H | H |
| Mathews 2023[78] | To describe the components of telehealth palliative care interventions for patients with advanced cancer before the COVID-19 pandemic, identify intervention components associated with improvements in outcomes, and evaluate the reporting of interventions. | Scoping review | 23 documents | Hybrid models, telephone-based interventions, videoconference-based interventions, asynchronous web-based intervention, mainly consisting of psychosocial content and delivered by nurses in the home setting. Participants included patients, family caregivers, and patient-caregiver dyads. | L | H |
| Maguire 2025, UK[79] | To explore the usability, user experiences, and impact of the digital dyadic remote monitoring Care and Support System for Patients and Carers (CARE-PAC) for patients in the last year of life, their informal carers, and health professionals involved in their care. | Mixed Methods Feasibility Study | Patients and informal carers | Digital dyadic remote monitoring Care and Support System for Patients and Carers (CARE-PAC) for patients in the last year of life. | H | H |
| Maudlin 2006, USA[80] | To determine if a telehealth model of care would benefit veteran patients with life-limiting illness by managing their physical, emotional, functional, and spiritual care needs during the last 2 years of their lives and fostering earlier enrolment into hospice through provider education on palliative care. | Pilot project collecting data on 100 veterans to evaluate the effectiveness of the Advanced Illness/Palliative Care (AIPC) program using home telehealth technology | veterans, patients, caregivers, family members | Technologies used included both video (telemonitors and videophones) and non-video (disease management and messaging) devices | L | H |
| Mccall 2013, UK[81] | To test the acceptability and usability of the Advanced Symptom Management System in Palliative Care (ASyMSp) in monitoring and managing symptoms of patients receiving palliative care at home. | Explorative descriptive study | patients, health professionals | Use of a mobile phone-based technology (ASyMSp) to monitor and manage symptoms reported by patients receiving palliative care at home, involving daily completion of an electronic questionnaire over a 30-day period. | M | H |
| Menon 2015. UDS[82] | To investigate the feasibility and describe patient outcomes of palliative care consultations via telemedicine in critically ill patients, assess the impact on decreasing the burden on family members, provide care in alignment with patient wishes, and conduct a prospective qualitative study in rural hospitals with a high risk of death. | A retrospective review of telemedicine family conferences among critically ill patients requested for transfer | patients, family members, doctors/clinicians | telemedicine palliative care conferences | L | L |
| Miller 2021[83] | To explore the existing literature on paediatric telemedicine home-based palliative care (TM HBPC) for children with serious illness to identify research, determine the effectiveness, and highlight the need for more robust information on implementation, adaptation, and maintenance of TM HBPC models. | Systematic scoping review | 17 documents | The intervention involved the delivery of home-based palliative care for children with serious illness via telemedicine, typically conducted by a hospital-based paediatric palliative care multidisciplinary team, with varying session durations and scheduling, and the use of different platforms for telemedicine consultations. | L | M |
| Morse 2021, Tanzania[84] | To design and develop mobile-Palliative Care Link (mPCL), a web and mobile app to support outpatient symptom assessment and care coordination and control, with a focus on pain. | human-centered iterative design framework | patients, caregivers, physicians, nurses, local health workers (LHWs) | Participants received the mPCL app for symptom assessment and care coordination, with some being provided loaned Android devices with the app preinstalled and others using their own smartphones to install the app. The intervention lasted for a 4-month study period. | H | H |
| Mukamal 2025[85] | To explore the experience of non-oncologic older adult patients in palliative care with telemedicine | Review | older adults | Telemedicine to enhance palliative care for older adults | M | M |
| Namasivayam 2022[86] | To review and map the available evidence on the use of telehealth in providing after-hours palliative care services in rural and remote Australia. | A scoping review | 12 documents | The intervention was the use of telehealth services provided by registered nurses, nurse specialists, GPs, specialist physicians, after-hours coordinators, and managers to enable patients to continue receiving after-hours palliative care at home in rural and remote Australia. | L | M |
| Narvaez 2022[87] | To map and evaluate existing research on the use of telemedicine and telehealth in palliative care patients during the COVID-19 outbreak. | Integrative review | 17 documents | Telehealth interventions including video conferencing, remote monitoring, and novel ways to improve care at home. | L | M |
| Nguyen 2020, USA[88] | To compare a tech-supported model with video consultations to a standard model of home-based palliative care to determine non-inferiority in terms of inter-professional team coordination and patient outcomes. | Pragmatic, cluster-randomized non-inferiority trial comparing tech-supported to standard home-based palliative care models | patients, nurses, doctors, family, caregivers | The intervention included nurse-led home visits coupled with remote physician consultation supervision via video for patients and caregivers in the tech-supported arm. Training on video visit procedures was provided to clinicians. | L | M |
| Oakley-Girvan 2021, USA[89] | To develop a mobile app (TOGETHERCare) that enables cancer survivors' care partners to monitor the survivors' health and provide resources for the care partners. | Product design study with user involvement | 138 stakeholders including 32 care partners, physicians, three cancer survivors | Use of the TOGETHERCare mobile app by cancer care partners for a 28-day feasibility field test to remotely monitor cancer survivors' health and their own health, as well as to provide care partner resources | H | M |
| Oelschlagel 2021, Norway[90] | To explore municipal healthcare professionals' experiences with implementing an application for remote care in palliative home care for patients with cancer. | qualitative, descriptive, exploratory design involving focus-group interviews and individual semi-structured interviews | Interdisciplinary HCP with experience of using RHC in home-based follow-up of patients with cancer who were in the palliative phase | Implementation of a technological solution named "remote home care" (RHC) in palliative home care for patients with cancer, including providing a tablet device, installing digital medical devices. | H | H |
| Oliver 2004, USA[91] | To assess the readiness of hospice organizations to accept technological innovation, specifically focusing on the current use of information and communication technology by hospice employees and their receptiveness to telemedicine or other advanced technology in patients' homes. | Cross-sectional survey study | Healthcare professionals working in hospice care: nurses, administrators, social workers, physicians, home health aides, chaplains. | No specific intervention | L | L |
| Oliver 2012, USA[92] | To assess the state of evidence related to tele hospice services and to highlight the need for strengthening the evidence base through various research methodologies. | Mixed methods study | patients, families, providers | Telephone advice lines, videophones, personal digital assistants, pen tablets, computers, comprehensive training for providers in the implementation of tele hospice projects | H | M |
| Olver 2005, Australia[93] | To understand nurses' experience | Qualitative study | Nurses - rural palliative care nurses and their more remote general nursing colleagues caring for palliative care patients in surrounding communities. | Videophone communication among rural palliative care nurses and their more remote general nursing colleagues | M | M |
| Osuji 2020, USA[94] | To assess the usefulness and appropriateness of video visits in home-based palliative care from the perspective of physicians and nurses, anchored within the NASSS framework. | Comparative effectiveness trial | physicians, nurses | Video visits were used primarily for follow-up visits as opposed to admission/start- of-care. Intervention: Video visits for follow-up care in home-based palliative care, facilitated by a nurse connecting patients with remote physicians | L | M |
| Paré 2009, Canada [95] | To evaluate the effects of a tele homecare intervention implemented in an oncology and palliative care unit in Quebec, Canada, focusing on nurses' satisfaction with the software application, perceived quality of care, and individual and group productivity. | Pre-post evaluation design | nurses | Implementation of the SyMO™ software with modules for care planning, developing treatment plan, nurse assignment, visit planning, route guidance, and documentation. | M | H |
| Parker 2021, Australia[96] | To evaluate the usefulness of CarerHelp from the perspective of health professionals caring for individuals with life-limiting illnesses. | Quantitative study | health professionals, specifically registered nurses working in specialist palliative care services | A web-based online toolkit for carers | M | M |
| Paul 2019, Canada[97] | To gain a preliminary understanding of the experience of using mobile web-based videoconferencing for conducting in-home palliative care consults with elderly rural patients with life-limiting illness, focusing on assessing the clinical effectiveness, acceptability, service impact, and technical feasibility of this innovative consultation format. | A descriptive, exploratory, proof-of-concept study | elderly patients at home | Palliative care consultations via mobile web-based videoconferencing (WBVC) with a palliative care physician consultant (PC-MD) from their homes, with the presence of a palliative care clinical nurse specialist (PC-CNS) and a home care nurse (HC-N) to manage the technology and support the patient. Participants also received training in the use of the technology. | L | H |
| Peñarrubia‐San‐Florencio 2025, Spain[98] | To explore family perspectives and experiences with telehealth in PPC, focusing on their needs, perspectives, concerns, and hopes to refine digital care models. | Qualitative study | primary caregivers (PCGs) of PPC patients. | Telehealth to support families caring for children with palliative needs. | H | H |
| Phillips 2008, Australia[99] | To evaluate the effectiveness of a generic after-hours telephone support service for palliative care patients and their caregivers in a rural setting in Australia, as well as to explore the application of a computer-based Palliative Care Clinical Information System to assist with the transfer of patient information. | Mixed-methods study | caregivers | After-Hours Telephone Support Service (AHTSS) provided to palliative care patients and their caregivers, operating 365 days per year from 1700 hours until 0830 hours, with an average call duration of 12.35 minutes and a total estimated time for an occasion of service being 30 minutes. | H | M |
| Phongtankuel 2018, USA[100] | To examine informal caregivers' receptivity and interest in using mHealth apps, along with identifying the app features caregivers perceived to be most useful in-home hospice care. | Qualitative study | Informal caregivers | Participants received the intervention of using mobile health applications (mHealth apps) with features such as video chat, medication/symptom tracking, and educational materials to improve communication and information exchange in home hospice care. | M | H |
| Pinto 2017 A[101] | To analyse the use of e-Health technologies and mobile apps in palliative care, focusing on the strengths, weaknesses, opportunities, and threats of these technologies. | Integrative literature review | a global review- 25 documents | e-Health technologies in palliative care used by adults, patients, nurses, doctors, family, carers, older people | H | M |
| Pinto 2017 B, Portugal[102] | To introduce a web-based application for monitoring comfort in patients receiving palliative care, particularly at home in Portugal. | multi-phase electronic application development process with pilot design | patients receiving palliative care | Monitoring comfort remotely using a web-based app, providing self-reported data on 11 comfort-related items on a Likert scale; using the app for 15 consecutive days, inputting data at least once a day; contacting the PC team in case of significant symptomatic distress; accessing the app through Android or Windows operating systems on smartphones, tablets, or computers; using the app to report symptoms and distress, with researchers monitoring the data. | H | H |
| Putranto 2020 [103] | To explore and synthesize scientific literature about cancer symptom management mobile apps for patients and their families, highlighting the role of nurses and the potential extension of mobile apps for managing other life-limiting illnesses. | Scoping review | 11 documents | The development and testing of mobile apps for managing cancer symptoms at home. Nurses are assigned to observe and assess patient symptoms through the mobile app. The main function of the mobile application system is to record an assessment of patient symptoms, provide education and skills training, and offer video media adjusted to the reported symptoms. | H | M |
| Rana 2025, USA[104] | To further explore adult patient and caregiver perspectives on telehealth, specifically the benefits of telehealth, trade-offs with in-person appointments, and the impact on patient care and family end-of-life preparations. | Qualitative study - single-arm prospective pilot study | adult patients | Telehealth to support palliative care services for Children and Young Adults With Cancer. | M | M |
| Royackers 2016, Canada[105] | To examine the experiences of informal caregivers who cared for a family member receiving palliative care as part of the eShift model of home care, focusing on their satisfaction with care delivery and ability to support their family members to die at home. | interpretive description methodology with semi-structured interviews | Informal caregivers | eShift model of home-based palliative care utilizing RNs, PSWs, and point-of-care technology with education provided to PSWs in palliative care. [registered nurses (RNs) and personal support workers (PSWs)] | M | H |
| Sadang 2023, India[106] | To investigate barriers and facilitators to implementing EIPC via telehealth by surveying palliative care clinicians participating in a large-scale multisite palliative care trial comparing in-person and video visit-based services. | Multisite randomized trial | clinicians specializing in palliative care and oncology | Early Integrated Palliative Care (EIPC) via secure video and/or in-person visits by advanced practice providers or board-certified physicians specializing in palliative care and/or oncology, at least monthly, beginning within 12 weeks of diagnosis of advanced lung cancer | L | H |
| Salem 2020, Lebanon[107] | To assess caregiver and provider perceptions of the safety and efficacy of the Distance Support Program (DSP) of a home-based palliative care provider in Beirut, Lebanon. | Qualitative study | patients, nurses, doctors, family caregivers | Distance Support Program (DSP) including linking patients to a primary provider, face-to-face encounter, 24/7 telephone access, provider-initiated contact, and communication via telephone, messaging, or videophone. | H | H |
| Salimian 2019, Iran [108] | To develop the initial version of a Mobile Application for cancer Palliative care for the purpose of improving the quality-of-life cancer patients. | mixed-method study | health information technology experts and oncologists | The mobile application "Ghasedak" for cancer palliative care, which includes functionalities such as user training on cancer and palliative care, clinician appointment reminders, personal health tracking, user guide, application settings, and patient notes. | H | L |
| Saysell 2003, UK[109] | To address the national shortage of consultants in palliative medicine in the UK by implementing a telemedicine project to provide access to consultant advice and support for clinical nurse specialists in palliative care at St David's Foundation Hospice Care. | Mixed-methods study | clinical nurse specialists (CNSs), district nurses, and general practitioners (GPs) | Weekly 2-hour teleconference sessions between CNSs and a consultant in palliative medicine to discuss patients with unresolved symptoms or significant palliative care issues. | L | H |
| Septian 2025[110] | To systematically synthesize the existing evidence from systematic reviews regarding: (a) the benefits of telehealth use on the quality of care and (b) the impact of telehealth on the quality of life of terminally ill patients. | Review |  | Telehealth to support terminally ill patients. | L | M |
| Schmitt 2022, USA[111] | To describe the demographics of paediatric patients receiving tele palliative care, assess patient and family satisfaction with telepalliative care, explore motives for using tele palliative services, and analyse themes present in patient and family discussions with palliative care providers. | Retrospective EMR chart review study | Fifty-six patients were seen through 181 tele palliative visits. | tele palliative visits - electronic medical record [Chart review was conducted using the electronic medical records (EMR) system, Epic Hyperspace. In 2016, the RCH EMR team embedded within the palliative outpatient note a data collection tool, Smart Form] | L | M |
| Schoppee 2020, USA [112] | To evaluate the acceptability of using the PAINReportIt completed on Wi-Fi-enabled tablets in the homes of end-stage cancer patients and their caregivers for reporting pain related to end-stage cancer, focusing on computer use acceptability scores and comparing them based on age, gender, race, and computer use experience. | Pretest/post-test study | hospice patients, caregivers | The intervention was the use of the PAINReportIt® tool on a Wi-Fi-enabled tablet in patients' homes for reporting pain in real-time to hospice nurses, with patients and caregivers completing the tool daily for a week. | M | M |
| Schuessler 2024[113] | To describe formal and informal caregivers' opportunities in tele-palliative care and explore the associated ethical and safety challenges using an integrative review method. | Integrative review with qualitative and quantitative designs | Nine documents from experimental and nonexperimental research were included | The interventions included teleconsultation via a teleconsultation device, a step-by-step teleconsultation intervention protocol, telehealth options via telephone or videophone, web-based information, or smartphone applications for patient self-management, and the ENABLE CHF-PC intervention involving face-to-face outpatient palliative care consultation and monthly telephone care coach sessions. | L | H |
| Slavin-stewart 2020, Canada[114] | To assess the feasibility of using FaceTime over a mobile cellular data network to provide improved access to specialist palliative care for patients in rural Nova Scotia, focusing on utilizing low-cost technology to enhance access to palliative care services in underserviced areas. | Observational study - quantitative descriptive | patients | Participants received regular home-based visits from a palliative care nurse who used the facetime application to connect with the palliative care physician in Halifax. The nurse utilized an Apple iPad with the facetime application and a cellular data package during these visits. | H | L |
| Steindal 2023[115] | To critically appraise and synthesize the findings from studies that investigated patients' use of telehealth in home-based palliative care, focusing on the advantages and challenges experienced by patients. | Systematic mixed studies review with a convergent design. | 41 documents from 40 studies | Telehealth technology for home-based palliative care, including video consultations in synchronous mode for teleconsultations and remote monitoring in asynchronous mode for symptom management. | M | H |
| Stern 2012, Canada[116] | To explore family caregivers and palliative cancer patients' patterns of use, perceptions of, and experiences with home telehealth in the context of palliative cancer care. | Mixed-methods case study with a focus on qualitative methods | The participant category : patients, family caregivers, and multidisciplinary healthcare professionals. | The intervention included specialist nurses available 24 hours per day who communicated with patients and families using videophones, with optional remote monitoring of vital signs such as blood pressure, blood oxygen levels, and heart, lung, and abdominal sounds. Communication was done via telephone or videophone as required. | M | H |
| Sutherland 2020[117] | To evaluate the use of video consultations as an alternative to face-to-face visits in palliative care during the Covid-19 pandemic. | Systematic review | one international and one national guideline and two systematic reviews | Video consultations using various technologies, cost reduction for patients, telehealth interventions (electronic patient records, telephone advice consultations, telehealth monitoring), and home visits with appropriate PPE. | L | L |
| Tasneem 2019, USA[118] | To assess patient perceptions on the impact of telemedicine video visits on care and to evaluate the ease of use and impact on health management of videoconference telemedicine for palliative care oncology patients in an urban setting. | Qualitative study | patients | The intervention that the study participants received was telemedicine video visits conducted through web-based face-to-face videoconferences. | L | H |
| Tarbi 2025, USA[119] | To explore if and how recommended in-person best practices for establishing human connection are adapted to the telehealth palliative care (telePC) setting, to enable discovery and description of practice innovations in this new care environment. | Qualitative study | clinicians | Telepalliative consultations that support clinician connection-building communication practices in rural telepalliative care encounters. | L | M |
| Tieman 2016, Australia[120] | To investigate the feasibility of a telehealth intervention for community-based palliative care patients, carers, and clinicians, with a focus on enhancing service delivery, monitoring symptoms, and improving care management in the home setting. The study aims to explore how telehealth can provide more regular engagement, continuous monitoring, and immediate access to resources for patients and families, ultimately aiming to improve the efficiency and effectiveness of palliative care delivery in the community. | Prospective cohort study | patients, carers, nurses, doctors, adults, family | Video-based conferences, virtual case conferences, self-report assessment tools, and remote activity monitoring. | H | L |
| Toenne 2025, Germany[121] | To evaluate the acceptance and challenges of implementing teleconsultations among specialised outpatient paediatric palliative care healthcare professionals. | Survey | Specialised outpatient paediatric palliative care nursing staff and physicians | Telehealth | M | H |
| Tong 2022, USA[122] | To evaluate the experiences of remote volunteer palliative care consultants during the initial COVID-19 surge, examining motivations, emotional toll, lessons learned, and providing insights for future efforts in extending remote palliative care services. | qualitative study | doctors | A palliative care virtual consultation program for high-complexity COVID-19 cases, mean duration of 15 days, mean of five patients per participant | L | H |
| Van Gurp 2013, Netherlands[123] | To describe elements of both the physical workplace and the cultural-social context of the palliative care practice, which are imperative for the use of teleconsultation technologies. | qualitative study | professionals in the hospital-based Expertise Centre for Palliative Care | Synchronous, audiovisual teleconsultation via webcam and iPad for palliative care patients at home | L | H |
| Van Gurp 2016, Netherlands[124] | To investigate how teleconsultation supports the integration of primary care, specialist palliative care, and patient perspectives and services, as well as how patients and informal caregivers experience collaboration through teleconsultation. | Qualitative study | patients, informal caregivers, primary care physicians (PCPs), and specialist palliative care team (SPCT) clinicians | Teleconsultation between home-based palliative care patients and a hospital-based specialist palliative care team (SPCT) conducted synchronously and audio visually, including tripartite consultations between the patient, primary care physician (PCP), and SPCT. | M | H |
| Verges 2022[125] | To describe the current use of telemedicine in palliative care and assess stakeholders' views on the initiatives implemented worldwide regarding digital service standards. | Scoping Review | 16 documents are included in this review | Telemedicine for patient and caregiver support, professional education, and routine follow-up in palliative care. | L | L |
| Vincent 2022, Canada[126] | To explore the experiences and perceptions of community palliative care providers, patients and caregivers who delivered or received virtual palliative care as a component of home-based palliative care during the COVID-19 pandemic. | Qualitative study using phone and video-based semi-structured interviews | Patients, caregivers, healthcare providers | virtual palliative care as a component of home-based palliative care during the COVID-19 pan- demic | L | H |
| Washington 2009, USA[127] | To measure tele hospice acceptance in a national sample of hospice professionals from various disciplines and to explore the usefulness and ease of use of tele hospice technology among different disciplinary backgrounds. | Cross-sectional study | hospice professionals from various disciplines such as nurses, administrators, social workers, and chaplains | The intervention was the use of videophone technology in the workplace for tele hospice services, as demonstrated to participants through a web-based streaming video. | L | H |
| Weaver 2021, USA[128] | To determine the acceptability of telehealth inclusion of a familiar paediatric palliative care provider during the first two home-based hospice visits to children, families, and adult-trained home hospice nurses in rural settings. | Case series | Children, family caregivers, and home hospice nurses | Virtual transition to home program with the inpatient paediatric palliative care physician joining the home hospice nurse's in-person visit as a telehealth presence using a facetime screen on day 1 and day 14 at home. | M | H |
| Whitten 2004, USA[129] | To investigate the use of tele hospice services from a patient's perspective, focusing on how tele hospice is utilized, reasons for patient declination, and patient satisfaction and preferences regarding tele hospice care. | Mixed methods | patients | Tele hospice services provided via videophones transmitting real-time video and audio images through the analogue phone system, including nursing, social work, and spiritual care tele visits | L | H |
| Williams 2024, USA[130] | To compare the provision of pediatric palliative care by delivery method. | A Retrospective Chart Review |  | Telehealth for pediatric palliative care. | H | H |
| Wood 2025[131] | To synthesise how, why, and in which circumstances can videoconferencing be used to successfully meet the emotional support needs of adults receiving palliative care? | Realist review |  | Video consultations i.e., video calls among adult patients and the bereaved to support emotions by palliative care organisations. | H | H |
| Xu 2023[132] | To evaluate users' satisfaction with telehealth palliative care during COVID-19 and to identify facilitators and barriers to telehealth implementation in palliative care during COVID-19. | Systematic mixed studies review | 18 documents were included | Telehealth services for palliative care using digital communication and information technologies such as computers, tablets, and mobile phone devices. | L | H |
| Zelko 2022, Austria[133] | To test the effectiveness of a new tool for exchanging information between professionals in palliative care on the primary healthcare level, with a focus on improving interdisciplinary cooperation and communication. | Mixed-methods study | healthcare providers, specifically medical doctors, family physicians, and nurses. | A smartphone tool for exchanging information | H | H |
| Zheng 2016[134] | To evaluate caregiver outcomes related to palliative telehealth interventions, with a focus on assessing the effectiveness of telehealth interventions for caregivers of patients in home-based palliative care. | Systematic review | 9 documents | Regular phones for counselling, Internet-based interventions, and unspecified telehealth devices were used in the study to provide support and communication to caregivers in the palliative care setting. | L | H |
| Zimmermann 2023, Germany[135] | To identify the needs and concerns of stakeholders regarding the introduction of telehealth in Paediatric Palliative Home Care (PPHC) and to define recommendations for telehealth in PPHC. | Mixed methods approach with a cross-sectional design | Children, adolescents, young adults, physicians, nurses, social workers, psychologists, patients, families, paediatricians, and other healthcare providers. | Telehealth interventions including video consultations, data transmission, access to patient records, symptom questionnaires, and communication support were provided to the study participants to improve the quality of care in paediatric palliative home care. | M | M |

**KEY**

The **relevance** of studies, in terms of contributions to programme theory was high (H) (well-aligned to the review questions, with rich descriptions contributing to theory); medium (M) (with some relevance to the review question and some insights to context-mechanism-outcome configurations) or low (L) (meeting broad inclusion criteria and providing at least one insight)

Concerning the **rigor**, quality assessment according to the Mixed Methods Appraisal Tool (MMAT) was scored based on five questions or criteria, with three responses for each item (i.e., 1=positive, 0 = negative or unavailable information). Accordingly, a score of five indicating high (H); 3 or 4 indicating moderate (M) and 2 and less indicating low quality (L) quality. For the systematic reviews included in the review, the Joanna Briggs Institute (JBI) quality assessment tool was used to generate an overall score (between 0 and 11). Using this tool, a score of eight or more indicates high quality (H), a score of six to eight indicates moderate quality (M) and a score less than six suggests low quality (L).

**Bibliography**

1.	World Health Organization. New Horizons for Health through Mobile Technologies [Internet]. Geneva: World Health Organization; 2011. Report No. Available from: http://whqlibdoc.who.int/publications/2011/9789241564250_ eng.pdf

2.	Kahveci̇ K, Koç O, Aksakal H. Home-based Palliative Care. Bezmialem Science. 2020 Jan 28;8(1):73–80. doi:10.14235/bas.galenos.2019.3208

3.	Roberts B, Robertson M, Ojukwu EI, Wu DS. Home Based Palliative Care: Known Benefits and Future Directions. Curr Geri Rep. 2021 Dec;10(4):141–7. doi:10.1007/s13670-021-00372-8

4.	Ventafridda V. According to the 2002 WHO Definition of Palliative Care... Palliat Med. 2006 Apr;20(3):159–159. doi:10.1191/0269216306pm1152ed

5.	Sleeman KE, De Brito M, Etkind S, Nkhoma K, Guo P, Higginson IJ, et al. The escalating global burden of serious health-related suffering: projections to 2060 by world regions, age groups, and health conditions. The Lancet Global Health. 2019 Jul;7(7):e883–92. doi:10.1016/S2214-109X(19)30172-X

6.	Harding R. Informal caregivers in home palliative care. Progress in Palliative Care. 2013 Sep;21(4):229–31. doi:10.1179/1743291X13Y.0000000056

7.	Finucane AM, O’Donnell H, Lugton J, Gibson-Watt T, Swenson C, Pagliari C. Digital health interventions in palliative care: a systematic meta-review. npj Digit Med. 2021 Apr 6;4(1):64. doi:10.1038/s41746-021-00430-7

8.	Nwosu AC, McGlinchey T, Sanders J, Stanley S, Palfrey J, Lubbers P, et al. Identification of Digital Health Priorities for Palliative Care Research: Modified Delphi Study. JMIR AGING. 2022;5(1). Located at: WOS:000798389400010. doi:10.2196/32075

9.	Lustbader D, Mudra M, Romano C, Lukoski E, Chang A, Mittelberger J, et al. The Impact of a Home-Based Palliative Care Program in an Accountable Care Organization. Journal of Palliative Medicine. 2017 Jan;20(1):23–8. doi:10.1089/jpm.2016.0265

10.	Abahussin AAbdulkarimM. Development of a Mobile Application-Based System for Recording Pain and Supporting Pain Management in Patients with Cancer. 2021. Located at: PQDT:50816589

11.	Allsop MJ, Namisango E, Powell RA. A survey of mobile phone use in the provision of palliative care services in the African region and priorities for future development. Journal of Telemedicine and Telecare. 2019;25(4):230–40. doi:10.1177/1357633X18767187

12.	Al-Mondhiry J, D’Ambruoso S, Pietras C, Strouse T, Benzeevi D, Arevian AC, et al. Co-created Mobile Apps for Palliative Care Using Community-Partnered Participatory Research: Development and Usability Study. JMIR Formative Research. 2022 Jun 23;6(6):e33849. doi:10.2196/33849

13.	Ansari N, Wilson CM, Heneghan MB, Supiano K, Mooney K. How technology can improve communication and health outcomes in patients with advanced cancer: an integrative review. SUPPORTIVE CARE IN CANCER. 2022;30(8):6525–43. Located at: WOS:000781011300001. doi:10.1007/s00520-022-07037-y

14.	Aoki N, Ohta S, Yamamoto H, Kikuchi N, Dunn K. Triangulation Analysis of Tele-Palliative Care Implementation in a Rural Community Area in Japan. Telemedicine and e-Health. 2006 Dec;12(6):655–62. doi:10.1089/tmj.2006.12.655

15.	Archer S, Cheung NHY, Williams I, Darzi A. The impact of digital health interventions on the psychological outcomes of patients and families receiving paediatric palliative care: A systematic review and narrative synthesis. Palliative Medicine. 2021;35(10):2017–23. doi:10.1177/02692163211026523

16.	Baldwin PK, McAuley WJ, Van Nostrand J. Organizational factors associated with the use of telehospice. J Telemed Telecare. 2012 Mar;18(2):90–3. doi:10.1258/jtt.2011.110803

17.	Balasubramanian S, Biji MS, Ranjith MK, Abhina SS. Patient satisfaction in Home care services through e-Palliative Care - An experience of tertiary cancer centre from Kerala. Indian Journal of Palliative Care. 2022;28(3):250–5. doi:10.25259/IJPC_36_2021

18.	Bandini JI, Scherling A, Farmer C, Fratkin M, Mistler B, Ast K, et al. Experiences with telehealth for outpatient palliative care: Findings from a mixed-methods study of patients and providers across the United States. Journal of Palliative Medicine. 2022;25(7):1079–87. doi:10.1089/jpm.2021.0545

19.	Basile I, Consolo L, Colombo S, Rusconi D, Rampichini F, Lusignani M. Technology to Support Older Adults in Home Palliative Care: A Scoping Review. AMERICAN JOURNAL OF HOSPICE & PALLIATIVE MEDICINE. 2023. Located at: WOS:001034330900001. doi:10.1177/10499091231189502

20.	Bauer EH, Schultz ANØ, Smith AC, Brandt F, Bollig G, Dieperink KB. Using Complex Interventions to Develop Programme Theory for a Family-Focused Model of Telepalliative Care. Scandinavian Journal of Caring Sciences. 2026;40(1):e70183. doi:10.1111/scs.70183

21.	Bensink M, Armfield N, Russell TG, Irving H, Wootton R. Paediatric palliative home care with Internet-based video-phones: lessons learnt. Journal of telemedicine and telecare. 2004;10 Suppl 1(9506702, cpj):10–3.

22.	Bensink M, Armfield N, Pinkerton R, Irving H, Hallahan A, Theodoros D, et al. Using videotelephony to support paediatric oncology-related palliative care in the home: from abandoned RCT to acceptability study. Palliat Med. 2009 Apr;23(3):228–37. doi:10.1177/0269216308100251

23.	Bethel C, Towers V, Crist JD, Silva GE, Shea K. A Guide for Intentional Home Telehealth Assessment: Patient and Caregiver Perceptions. CIN: Computers, Informatics, Nursing. 2021;39(12):943–7. doi:10.1097/CIN.0000000000000779

24.	Bhargava R, Keating B, Isenberg SR, Subramaniam S, Wegier P, Chasen M. RELIEF: A Digital Health Tool for the Remote Self-Reporting of Symptoms in Patients with Cancer to Address Palliative Care Needs and Minimize Emergency Department Visits. Current Oncology. 2021 Oct 21;28(6):4273–80. doi:10.3390/curroncol28060363

25.	Bonsignore L, Bloom N, Steinhauser K, Nichols R, Allen T, Twaddle M, et al. Evaluating the feasibility and acceptability of a telehealth program in a rural palliative care population: TapCloud for palliative care. Journal of Pain and Symptom Management. 2018;56(1):7–14. doi:10.1016/j.jpainsymman.2018.03.013

26.	Bradford NK, Armfield NR, Young J, Smith AC. Paediatric palliative care by video consultation at home: a cost minimisation analysis. BMC Health Serv Res. 2014 Dec;14(1):328. doi:10.1186/1472-6963-14-328

27.	Bradford NK, Armfield NR, Young J, Herbert A, Mott C, Smith AC. Principles of a paediatric palliative care consultation can be achieved with home telemedicine. Journal of Telemedicine and Telecare. 2014;20(7):360–4. doi:10.1177/1357633X14552370

28.	Brännström M, Philipsson J, Andersson S. Healthcare professionals’ experiences of video consultations in palliative care in rural areas: an intervention study in community care. BMC Health Serv Res. 2024 Jun 17;24(1):740. doi:10.1186/s12913-024-11196-5

29.	Burner-Fritsch I, Bolzani A, Hriskova K, Kolmhuber S, Bausewein C, Hodiamont F. Challenges developing an electronic patient-reported outcome measurement for palliative home care: A qualitative interview and focus group study. Palliative Medicine. 2023;37(2):265–74. doi:10.1177/02692163221141487

30.	Caetano P, Querido A, Laranjeira C. Preparedness for Caregiving Role and Telehealth Use to Provide Informal Palliative Home Care in Portugal: A Qualitative Study. Healthcare. 2024 Jan;12(19):1915. doi:10.3390/healthcare12191915

31.	Calton BA. Communication Strategies for Providing Palliative Care via Telemedicine #406. Journal of Palliative Medicine. 2021;24(1):135–6. doi:10.1089/jpm.2020.0582

32.	Cameron P. Caregiver Comfort With Telehospice. Home Healthcare Now. 2021;39(1):32–8. doi:10.1097/NHH.0000000000000928

33.	Carey N, Abathun E, Maguire R, Wodaje Y, Royce C, Ayers N. Co-design and prototype development of the ‘Ayzot App’: A mobile phone based remote monitoring system for palliative care. Palliat Med. 2023 May 1;37(5):771–81. doi:10.1177/02692163231162408

34.	Christiansen L, Lindberg C, Sanmartin Berglund J, Anderberg P, Skär L. Using Mobile Health and the Impact on Health-Related Quality of Life: Perceptions of Older Adults with Cognitive Impairment. IJERPH. 2020 Apr 13;17(8):2650. doi:10.3390/ijerph17082650

35.	Chua IS, Olmsted M, Plotke R, Turk Y, Trotter C, Rinaldi S, et al. Video and in-person palliative care delivery challenges before and during the COVID-19 pandemic. Journal of Pain and Symptom Management. 2022;64(6):577–87. doi:10.1016/j.jpainsymman.2022.08.005

36.	Collier A, Morgan DD, Swetenham K, To THM, Currow DC, Tieman JJ. Implementation of a pilot telehealth programme in community palliative care: A qualitative study of clinicians’ perspectives. Palliative Medicine. 2016;30(4):409–17. doi:10.1177/0269216315600113

37.	Collier A, Appachoo S, Frey R, Jones V, Birtwistle J, Allsop M, et al. Extending the reach of palliative care–a double–edged sword: a qualitative study of clinicians’ experiences of delivering telehealth in Australia and Aotearoa New Zealand. BMC Palliat Care. 2025 Nov 13;24(1):288. doi:10.1186/s12904-025-01932-x

38.	Cook D, Doolittle G, Whitten P. Administrator and provider perceptions of the factors relating to programme effectiveness in implementing telemedicine to provide end-of-life care. JOURNAL OF TELEMEDICINE AND TELECARE. 2001;7:17–9. Located at: WOS:000172652000008. doi:10.1258/1357633011937335

39.	Coyle N, Khojainova N, Francavilla JM, Gonzales GR. Audio-visual communication and its use in palliative care. Journal of Pain and Symptom Management. 2002;23(2):171–5. doi:10.1016/S0885-3924%252801%252900402-X

40.	Cruz S, Fernandes C, Magalhães B. A scoping review of mobile apps for use with palliative patients in the context of home care. International Journal of Medical Informatics. 2023 Sep;177:105166. doi:10.1016/j.ijmedinf.2023.105166

41.	Davis MS, Harrison KL, Rice JF, Logan A, Hess B, Fine PG, et al. A Model for Effective and Efficient Hospice Care: Proactive Telephone-Based Enhancement of Life Through Excellent Caring, “TeleCaring” in Advanced Illness. Journal of pain and symptom management. 2015;50(3):414–8. doi:10.1016/j.jpainsymman.2015.03.012

42.	Dickman Portz J, Ford K, Bekelman DB, Boxer RS, Kutner JS, Czaja S, et al. “We’re Taking Something So Human and Trying to Digitize”: Provider Recommendations for mHealth in Palliative Care. Journal of Palliative Medicine. 2020 Feb 1;23(2):240–7. doi:10.1089/jpm.2019.0216

43.	Disalvo D, Agar M, Caplan G, Murtagh FEM, Luckett T, Heneka N, et al. Virtual models of care for people with palliative care needs living in their own home: A systematic meta-review and narrative synthesis. Palliative Medicine. 2021;35(8):1385–406. doi:10.1177/02692163211024451

44.	Dismore L, Frew K, Wakefield D, Bryan C, Swainston K. Remote and rural communities face inequalities in access to specialist palliative care, could telemedicine enhance care? A qualitative study of patient, carer and healthcare professionals’ experiences of video consultation. Palliat�Care. 2025 Nov 1;19:26323524251380632. doi:10.1177/26323524251380632

45.	Dolan H, Eggett C, Holliday L, Delves S, Parkes D, Sutherland K. Virtual care in end of life and palliative care: A rapid evidence check. J Telemed Telecare. 2021 Dec 1;27(10):631–7. doi:10.1177/1357633X211046118

46.	Domínguez F, Heras J, Benjumea J, Vallejo M, Parra E, Fiallos W, et al. Evaluating a Wearable-Based Pain Monitoring System in Palliative Cancer Care: Usability and Feasibility Study. JMIR Form Res. 2026 Feb 6;10:e78098. doi:10.2196/78098 PubMed PMID: 41650128; PubMed Central PMCID: PMC12880589.

47.	Doolittle GC, Nelson EL, Spaulding AO, Lomenick AF, Krebill HM, Adams NJ, et al. TeleHospice: A community-engaged model for utilizing mobile tablets to enhance rural hospice care. American Journal of Hospice & Palliative Medicine. 2019;36(9):795–800. doi:10.1177/1049909119829458

48.	Evering RMH, Postel MG, van Os-Medendorp H, Bults M, den Ouden MEM. Intention of healthcare providers to use video-communication in terminal care: a cross-sectional study. BMC PALLIATIVE CARE. 2022;21(1). Located at: WOS:000892990600001. doi:10.1186/s12904-022-01100-5

49.	Gonzalez AF. Evaluating Usability of a Home Health Tablet Device Among Adult Palliative Care Patients. 2023. Located at: PQDT:82914264

50.	Gordon B, Mason B, Smith SLH. Leveraging Telehealth for Delivery of Palliative Care to Remote Communities: A Rapid Review. JOURNAL OF PALLIATIVE CARE. 2022;37(2):213–25. Located at: WOS:000630577200001. doi:10.1177/08258597211001184

51.	Guo J, Dai Y, Gong Y, Xu X, Chen Y. Exploring the telehealth readiness and its related factors among palliative care specialist nurses: a cross-sectional study in China. BMC PALLIATIVE CARE. 2023;22(1). Located at: WOS:001018505300001. doi:10.1186/s12904-023-01209-1

52.	Hancock S, Preston N, Jones H, Gadoud A. Telehealth in palliative care is being described but not evaluated: a systematic review. BMC PALLIATIVE CARE. 2019;18(1). Located at: WOS:000511999200001. doi:10.1186/s12904-019-0495-5

53.	Harding R, Carrasco JM, Serrano-Pons J, Lemaire J, Namisango E, Luyirika E, et al. Design and Evaluation of a Novel Mobile Phone Application to Improve Palliative Home-Care in Resource-Limited Settings. Journal of Pain & Symptom Management. 2021;62(1):1–9. doi:10.1016/j.jpainsymman.2020.09.045

54.	Haroen H, Juniarti N, Sari CWM, Sari SP, Harun H, Cahyati P, et al. Integrating Telehealth into Community-Based Palliative Care: A Systematic Review. Journal of Multidisciplinary Healthcare. 2025 Nov 6;18:7339–54. doi:10.2147/JMDH.S555226

55.	Haydon HM, Snoswell CL, Thomas EE, Broadbent A, Caffery LJ, Brydon JA, et al. Enhancing a community palliative care service with telehealth leads to efficiency gains and improves job satisfaction. Journal of Telemedicine and Telecare. 2021;27(10):625–30. doi:10.1177/1357633X211048952

56.	Hebert MA, Paquin MJ, Whitten L, Cai P. Analysis of the suitability of “video-visits” for palliative home care: implications for practice. J Telemed Telecare. 2007 Mar 1;13(2):74–8. doi:10.1258/135763307780096203

57.	Hennemann-Krause L, Lopes AJ, Araújo JA, Petersen EM, Nunes RA. The assessment of telemedicine to support outpatient palliative care in advanced cancer. Palliative & supportive care. 2015;13(4):1025–30.

58.	Hoek PD, Schers HJ, Bronkhorst EM, Vissers KCP, Hasselaar JGJ. The effect of weekly specialist palliative care teleconsultations in patients with advanced cancer –a randomized clinical trial. BMC Med. 2017 Dec;15(1):119. doi:10.1186/s12916-017-0866-9

59.	Holland DE, Vanderboom CE, Ingram CJ, Dose AM, Borkenhagen LS, Skadahl P, et al. The Feasibility of Using Technology to Enhance the Transition of Palliative Care for Rural Patients. CIN-COMPUTERS INFORMATICS NURSING. 2014;32(6):257–66. Located at: WOS:000338987000002. doi:10.1097/CIN.0000000000000066

60.	Holmen H, Riiser K, Winger A. Home-Based Pediatric Palliative Care and Electronic Health: Systematic Mixed Methods Review. Journal of Medical Internet Research. 2020;22(2):N.PAG-N.PAG. doi:10.2196/16248

61.	Hutchinson RN, Anderson EC, Ruben MA, Manning N, John L, Daruvala A, et al. A formative mixed-methods study of emotional responsiveness in telepalliative care. Journal of Palliative Medicine. 2022;25(8):1258–67. doi:10.1089/jpm.2021.0589

62.	Jess M, Timm H, Dieperink KB. Video consultations in palliative care: A systematic integrative review. Palliative Medicine. 2019;33(8):942–58. doi:10.1177/0269216319854938

63.	Jiang B, Bills M, Poon P. Integrated telehealth-assisted home-based specialist palliative care in rural Australia: A feasibility study. JOURNAL OF TELEMEDICINE AND TELECARE. 2023;29(1):50–7. Located at: WOS:000583681600001. doi:10.1177/1357633X20966466

64.	Johnston B. UK Telehealth Initiatives in Palliative Care: a Review. Int J Palliat Nurs. 2011 Jun;17(6):301–8. doi:10.12968/ijpn.2011.17.6.301

65.	Johnston B, Kidd L, Wengstrom Y, Kearney N. An evaluation of the use of Telehealth within palliative care settings across Scotland. Palliative medicine. 2012;26(2):152–61. doi:10.1177/0269216311398698

66.	Johnston B. Palliative home-based technology from a practitioner&#39;s perspective: benefits and disadvantages. SHTT. 2014 Nov;121. doi:10.2147/SHTT.S42687

67.	Funderskov KF, Boe Danbjørg D, Jess M, Munk L, Olsen Zwisler A, Dieperink KB. Telemedicine in specialised palliative care: Healthcare professionals’ and their perspectives on video consultations—A qualitative study. Journal of Clinical Nursing (John Wiley & Sons, Inc). 2019;28(21/22):3966–76. doi:10.1111/jocn.15004

68.	Kawashima A, Noguchi T, Furukawa T, Unesoko A, Togashi S, Sato K. Effectiveness of and implementation requirements for telehealth in palliative care patients with advanced cancer: A systematic review and meta-analysis. Palliat Med. 2025 Dec 30;02692163251403395. doi:10.1177/02692163251403395

69.	Keenan J. The feasibility and acceptability of telehealth to support palliative care in Mid-Wales: Exploring professional perceptions and patient experiences [PhD Thesis] [Internet]. Aberystwyth University; 2016 [cited 2026 Mar 4]. Available from: https://www.researchgate.net/profile/Joseph-Keenan-3/publication/309772079_The_feasibility_and_acceptability_of_telehealth_to_support_palliative_care_in_Mid-Wales_Exploring_professional_perceptions_and_patient_experiences/links/5823095808ae7ea5be71bfca/The-feasibility-and-acceptability-of-telehealth-to-support-palliative-care-in-Mid-Wales-Exploring-professional-perceptions-and-patient-experiences.pdf

70.	Kidd L, Cayless S, Johnston B, Wengstrom Y. Telehealth in palliative care in the UK: A review of the evidence. Journal of Telemedicine and Telecare. 2010;16(7):394–402. doi:10.1258/jtt.2010.091108

71.	Kluger BM, Katz M, Galifianakis NB, Pantilat SZ, Hauser JM, Khan R, et al. Patient and family outcomes of community neurologist palliative education and telehealth support in Parkinson disease. JAMA Neurology. 2024;81(1):39–49. doi:10.1001/jamaneurol.2023.4260

72.	Kwon SH, Lee MA, Kim YJ, Park SH, Lee MJ, Han Y, et al. Evaluation of Hospice@Home for Home-Based Palliative Care: Development and Usability Pilot Study. JMIR Formative Research. 2026 Jan 21;10(1):e79334. doi:10.2196/79334

73.	Middleton-Green L., Gadoud A., Norris B., Sargeant A., Nair S., Wilson L., et al. “A Friend in the Corner”: supporting people at home in the last year of life via telephone and video consultation-an evaluation. BMJ supportive & palliative care. 2019;9(4):e26. Located at: 630057486. doi:10.1136/bmjspcare-2015-001016

74.	Lee S, Woo S, Song M, Lee M, Joo J, Kim D, et al. Effect of Early Management on Pain and Depression in Patients with Pancreatobiliary Cancer: a Randomized Clinical Trial. Vol. 11. 2017;11:39. Located at: CN-02663601

75.	Lind L, Karlsson D, Fridlund B. Patients’ use of digital pens for pain assessment in advanced palliative home healthcare. International Journal of Medical Informatics. 2008 Feb;77(2):129–36. doi:10.1016/j.ijmedinf.2007.01.013

76.	Lundereng E.D., Nes A.A.G., Holmen H., Winger A., Thygesen H., Joranson N., et al. Health Care Professionals’ Experiences and Perspectives on Using Telehealth for Home-based Palliative Care: Scoping Review. Journal of Medical Internet Research. 2023;25((Lundereng, Nes, Mariussen, Steindal) Lovisenberg Diaconal University College, Oslo, Norway(Lundereng, Dajani) European Palliative Care Research Centre (PRC), Department of Oncology, Oslo University Hospital, Oslo, Norway(Holmen, Winger) Faculty of Health):e43429. Located at: 2023667556. doi:10.2196/43429

77.	Ma C, Fang Y, Zhang H, Zheng Y, Zhang Y, Zhao W, et al. Nurse-Delivered Telehealth in Home-Based Palliative Care: Integrative Systematic Review. Journal of Medical Internet Research. 2025 May 5;27(1):e73024. doi:10.2196/73024

78.	Mathews JJ, Chow R, Wennberg E, Lau J, Hannon B, Zimmermann C. Telehealth palliative care interventions for patients with advanced cancer: a scoping review. SUPPORTIVE CARE IN CANCER. 2023;31(8). Located at: WOS:001025022200004. doi:10.1007/s00520-023-07907-z

79.	Maguire R, McCann L, Singleton C, Perkins P, Minton O, McCann N, et al. Exploring the Impact of a Remote Monitoring System for Palliative and End-of-Life Care (CARE-PAC): Mixed Methods Feasibility Study. JMIR Formative Research. 2025 Sep 23;9(1):e69394. doi:10.2196/69394

80.	Maudlin J, Keene J, Kobb R. A Road Map for the Last Journey: Home Telehealth for Holistic End-of-Life Care. Am J Hosp Palliat Care. 2006 Oct;23(5):399–403. doi:10.1177/1049909106290807

81.	McCall K, Keen J, Farrer K, Maguire R, McCann L, Johnston B, et al. Perceptions of the use of a remote monitoring system in patients receiving palliative care at home. Int J Palliat Nurs. 2008 Sep;14(9):426–31. doi:10.12968/ijpn.2008.14.9.31121

82.	Menon PR, Stapleton RD, McVeigh U, Rabinowitz T. Telemedicine as a tool to provide family conferences and palliative care consultations in critically ill patients at rural health care Institutions: A pilot study. American Journal of Hospice & Palliative Medicine. 2015;32(4):448–53. doi:10.1177/1049909114537110

83.	Miller KA, Baird J, Lira J, Herrera Eguizabal J, Fei S, Kysh L, et al. The Use of Telemedicine for Home-Based Palliative Care for Children With Serious Illness: A Scoping Review. Journal of Pain & Symptom Management. 2021;62(3):619–619. doi:10.1016/j.jpainsymman.2020.12.004

84.	Morse RS, Lambden K, Quinn E, Ngoma T, Mushi B, Ho YX, et al. A Mobile App to Improve Symptom Control and Information Exchange Among Specialists and Local Health Workers Treating Tanzanian Cancer Patients: Human-Centered Design Approach. JMIR Cancer. 2021 Mar 23;7(1):e24062. doi:10.2196/24062

85.	Mukamal RC, Dias LM, Sarti TD, Nunes R, Rego G. The role of telemedicine in enhancing palliative care for older adults: opportunities and challenges. BMC Geriatr. 2025 Jul 2;25(1):474. doi:10.1186/s12877-025-06041-1

86.	Namasivayam P, Bui DT, Low C, Barnett T, Bridgman H, Marsh P, et al. Use of telehealth in the provision of after-hours palliative care services in rural and remote Australia: A scoping review protocol. PLOS ONE. 2022;17(1). Located at: WOS:000796264200037. doi:10.1371/journal.pone.0261962

87.	Narvaez RA, Canaria A, Nifras SR, Listones N, Topacio R, Baua ME. The role of telehealth on quality of life of palliative care patients during the COVID-19 pandemic: an integrative review. Int J Palliat Nurs. 2022 Dec 2;28(12):583–9. doi:10.12968/ijpn.2022.28.12.583

88.	Nguyen HQ, McMullen C, Haupt EC, Wang SE, Werch H, Edwards PE, et al. Findings and lessons learnt from early termination of a pragmatic comparative effectiveness trial of video consultations in home-based palliative care. BMJ Support Palliat Care. 2022 Aug;12(e3):e432–40. doi:10.1136/bmjspcare-2020-002553

89.	Oakley-Girvan I, Davis SW, Kurian A, Rosas LG, Daniels J, Palesh OG, et al. Development of a Mobile Health App (TOGETHERCare) to Reduce Cancer Care Partner Burden: Product Design Study. JMIR Form Res. 2021 Aug 13;5(8):e22608. doi:10.2196/22608

90.	Oelschlägel L, Dihle A, Christensen VL, Heggdal K, Moen A, Österlind J, et al. Implementing welfare technology in palliative homecare for patients with cancer: a qualitative study of health-care professionals’ experiences. BMC Palliat Care. 2021 Dec;20(1):146. doi:10.1186/s12904-021-00844-w

91.	Oliver D, Demiris G. An assessment of the readiness of hospice organizations to accept technological innovation. JOURNAL OF TELEMEDICINE AND TELECARE. 2004;10(3):170–4. Located at: WOS:000222027100010

92.	Oliver DP, Demiris G, Wittenberg-Lyles E, Porock D. The use of videophones for patient and family participation in hospice interdisciplinary team meetings: a promising approach. EUROPEAN JOURNAL OF CANCER CARE. 2010;19(6):729–35. Located at: WOS:000282880200006. doi:10.1111/j.1365-2354.2009.01142.x

93.	Olver I, Brooksbank M, Champion N, Keeley J. The use of videophones to enhance palliative care outreach nursing in remote areas. Progress in Palliative Care. 2005 Oct;13(5):263–7. doi:10.1179/096992605X57679

94.	Pare G., Sicotte C., Chekli M., Jaana M., De Blois C., Bouchard M. A pre-post evaluation of a telehomecare program in oncology and palliative care. Telemedicine and e-Health. 2009;15(2):154–9. Located at: 354341285. doi:10.1089/tmj.2008.0091

95.	Pare G., Sicotte C., Chekli M., Jaana M., De Blois C., Bouchard M. A pre-post evaluation of a telehomecare program in oncology and palliative care. Telemedicine and e-Health. 2009;15(2):154–9. Located at: 354341285. doi:10.1089/tmj.2008.0091

96.	Parker D, Hudson P, Tieman J, Thomas K, Saward D, Ivynian S. Evaluation of an online toolkit for carers of people with a life-limiting illness at the end-of-life: health professionals’ perspectives. Australian Journal of Primary Health. 2021;27(6):473–8. doi:10.1071/PY21019

97.	Paul L.R., Walia R., Porquet-Seitz N., Loftus R., Webb M., Ho G., et al. Rural Home Care Palliative Videoconsultation Project - Phase 2. Journal of Pain and Symptom Management. 2018;56(6):e103–4. Located at: 2001334858. doi:10.1016/j.jpainsymman.2018.10.349

98.	Peñarrubia-San-Florencio L, Junior CG, Ricart S, Vilarrubí SN, Ruiz-Herguido C, Esteve AV, et al. Family perspectives and experiences on implementing telehealth in pediatric palliative care: a qualitative approach. Eur J Pediatr. 2025 Apr 8;184(5):287. doi:10.1007/s00431-025-06124-6

99.	Phillips JL, Davidson PM, Newton PJ, DiGiacomo M. Supporting patients and their caregivers after-hours at the end of life: The role of telephone support. Journal of Pain and Symptom Management. 2008;36(1):11–21. doi:10.1016/j.jpainsymman.2007.08.017

100.	Phongtankuel V, Shalev A, Adelman RD, Dewald R, Dignam R, Baughn R, et al. Mobile health technology is here-But are hospice informal caregivers receptive? American Journal of Hospice & Palliative Medicine. 2018;35(12):1547–52. doi:10.1177/1049909118779018

101.	Pinto S, Caldeira S, Martins JC. e-Health in palliative care: review of literature, Google Play and App Store. INTERNATIONAL JOURNAL OF PALLIATIVE NURSING. 2017;23(8):394–401. Located at: WOS:000408656300005. doi:10.12968/ijpn.2017.23.8.394

102.	Pinto S, Almeida F, Caldeira S, Martins JC. The Comfort app prototype: introducing a web-based application for monitoring comfort in palliative care. Int J Palliat Nurs. 2017 Sep 2;23(9):420–31. doi:10.12968/ijpn.2017.23.9.420

103.	Putranto D, Rochmawati E. Mobile applications for managing symptoms of patients with cancer at home: A scoping review. International Journal of Nursing Practice (John Wiley & Sons, Inc). 2020;26(4):1–14. doi:10.1111/ijn.12842

104.	Rana S, DeGroote NP, Harris E, Lange A, Wasilewski-Masker K, Klosky JL, et al. Interview Results From a Telehealth Hospice Intervention for Children and Young Adults With Cancer. Journal of Pain and Symptom Management. 2025 Apr 1;69(4):343-353.e4. doi:10.1016/j.jpainsymman.2024.12.018

105.	Royackers A, Regan S, Donelle L. The eShift model of care: informal caregivers’ experience of a new model of home-based palliative care. Progress in Palliative Care. 2016;24(2):84–92. doi:10.1179/1743291X15Y.0000000006

106.	Sadang KG, Centracchio JA, Turk Y, Park E, Feliciano JL, Chua IS, et al. Clinician Perceptions of Barriers and Facilitators for Delivering Early Integrated Palliative Care via Telehealth. CANCERS. 2023;15(22). Located at: WOS:001119928400001. doi:10.3390/cancers15225340

107.	Salem R, El Zakhem A, Gharamti A, Tfayli A, Osman H. Palliative Care via Telemedicine: A Qualitative Study of Caregiver and Provider Perceptions. Journal of Palliative Medicine. 2020 Dec 1;23(12):1594–8. doi:10.1089/jpm.2020.0002

108.	Salimian N, Ehteshami A, Ashouri-Talouki M. Developing Ghasedak: a Mobile Application to Improve the Quality of Cancer Palliative Care. Acta informatica medica : AIM : journal of the Society for Medical Informatics of Bosnia & Herzegovina : casopis Drustva za medicinsku informatiku BiH. 2019;27(1):19–22. Located at: MEDLINE:31213738. doi:10.5455/aim.2019.27.19-22

109.	Saysell E., Routley C. Telemedicine in community-based palliative care: evaluation of a videolink teleconference project. International journal of palliative nursing. 2003;9(11):489–95. Located at: 137617780

110.	Septian RB, Suryana R, Rukhama RA, Destyanto AA, Librianty R. The Use of Telehealth in Home-Based Palliative Care: An Umbrella Review of Systematic Reviews.

111.	Schmitt S, Ebby C, Doshi A, Bower K, Marc-Aurele K. Retrospective Pediatric Telepalliative Care Experience. Journal of Palliative Medicine. 2022 Feb 1;25(2):274–81. doi:10.1089/jpm.2021.0173

112.	Schoppee TM, Dyal BW, Scarton L, Ezenwa MO, Singh P, Yao Y, et al. Patients and caregivers rate the PAINReportIt wireless internet-enabled tablet as a method for reporting pain during end-of-life cancer care. Cancer Nursing. 2020;43(5):419–24. doi:10.1097/NCC.0000000000000743

113.	Schuessler N, Glarcher M. Caregivers’ Perspectives on Ethical Challenges and Patient Safety in Tele-Palliative Care. JOURNAL OF HOSPICE & PALLIATIVE NURSING. 2024;26(1):E1–12. Located at: WOS:001141983300003. doi:10.1097/NJH.0000000000000986

114.	Slavin-Stewart C, Phillips A, Horton R. A Feasibility Study of Home-Based Palliative Care Telemedicine in Rural Nova Scotia. Journal of Palliative Medicine. 2020;23(4):548–51. doi:10.1089/jpm.2019.0173

115.	Steindal SA, Nes AAG, Godskesen TE, Holmen H, Winger A, Osterlind J, et al. Advantages and Challenges of Using Telehealth for Home-Based Palliative Care: Systematic Mixed Studies Review. Journal of medical Internet research. 2023;25(100959882):e43684. doi:10.2196/43684

116.	Stern A, Valaitis R, Weir R, Jadad AR. Use of home telehealth in palliative cancer care: a case study. Journal of Telemedicine & Telecare. 2012;18(5):297–300. doi:10.1258/jtt.2012.111201

117.	Sutherland A.E., Stickland J., Wee B. Can video consultations replace face-to-face interviews? Palliative medicine and the Covid-19 pandemic: rapid review. BMJ supportive & palliative care. 2020;10(3):271–5. Located at: 631909371. doi:10.1136/bmjspcare-2020-002326

118.	Tasneem S, Kim A, Bagheri A, Lebret J. Telemedicine video visits for patients receiving palliative care: A qualitative study. American Journal of Hospice & Palliative Medicine. 2019;36(9):789–94. doi:10.1177/1049909119846843

119.	Tarbi EC, Durieux BN, Brain JM, Kwok A, Umeton R, Samineni S, et al. Measuring palliative care communication via telehealth: A pilot study. Journal of Pain and Symptom Management. 2023;66(1):e155–61. doi:10.1016/j.jpainsymman.2023.04.001

120.	Tieman JJ, Swetenham K, Morgan DD, To TH, Currow DC. Using telehealth to support end of life care in the community: a feasibility study. BMC Palliative Care. 2016;15:1–7. doi:10.1186/s12904-016-0167-7

121.	Toenne R, Neuhaus U, Höcker M, Koch A, Menzel A, Scharfe D, et al. Exploring the viability of telehealth integration into specialised paediatric palliative care. Int J Palliat Nurs. 2025 Feb 2;31(2):58–67. doi:10.12968/ijpn.2025.31.2.58

122.	Tong W, Kapur S, Fleet A, Russo S, Khedagi A, Blinderman CD, et al. Perspectives of remote volunteer palliative care consultants during COVID: A qualitative study. Journal of Pain and Symptom Management. 2022;63(2):321–9. doi:10.1016/j.jpainsymman.2021.07.028

123.	van Gurp J, Hasselaar J, van Leeuwen E, Hoek P, Vissers K, van Selm M. Connecting with patients and instilling realism in an era of emerging communication possibilities: A review on palliative care communication heading to telecare practice. Patient Education and Counseling. 2013;93(3):504–14. doi:10.1016/j.pec.2013.07.002

124.	Van Gurp J, Van Selm M, Van Leeuwen E, Vissers K, Hasselaar J. Teleconsultation for integrated palliative care at home: A qualitative study. Palliat Med. 2016 Mar;30(3):257–69. doi:10.1177/0269216315598068

125.	Salvador Verges A, Cusi Sanchez MV, Bossio Grigera P, Fabrega Agullo C, Gomes da Costa F, Serra Trullas A, et al. Determinants in stakeholder opinions about telemedicine in palliative care: A scoping review. Telemedicine and e-Health. 2022;28(7):932–41. doi:10.1089/tmj.2021.0441

126.	Vincent D, Peixoto C, Quinn KL, Kyeremanteng K, Lalumiere G, Kurahashi AM, et al. Virtual home-based palliative care during COVID-19: A qualitative exploration of the patient, caregiver, and healthcare provider experience. Palliative Medicine. 2022;36(9):1374–88. doi:10.1177/02692163221116251

127.	Washington KT, Demiris G, Oliver DP, Day M. Telehospice acceptance among providers: A multidisciplinary comparison. American Journal of Hospice & Palliative Medicine. 2009;25(6):452–7. doi:10.1177/1049909108322289

128.	Weaver MS, Rosenberg AR, Fry A, Shostrom V, Wiener L. Impact of the coronavirus pandemic on pediatric palliative care team structures, services, and care delivery. Journal of Palliative Medicine. 2021;24(8):1213–20. doi:10.1089/jpm.2020.0589

129.	Whitten P, Doolittle G, Mackert M. Telehospice in Michigan: Use and patient acceptance. American Journal of Hospice & Palliative Medicine. 2004;21(3):191–5. doi:10.1177/104990910402100307

130.	Williams VA. The Effects of Transformational Leadership on Virtual Onboarding Within the U.S. Insurance Industry [PhD Thesis]. Tiffin University; 2023.

131.	Wood MJM. Connecting and Belonging: Using Technology for Art Therapy in Palliative Care. In: The International Handbook of Art Therapy in Palliative and Bereavement Care. Routledge; 2019.

132.	Xu X, Ho MH, Lin CC. Telehealth in palliative care during the COVID-19 pandemic: A systematic mixed studies review. WORLDVIEWS ON EVIDENCE-BASED NURSING. 2023;20(5):476–91. Located at: WOS:000953801800001. doi:10.1111/wvn.12637

133.	Zelko E, Pajk JR, Skvarc NK. An Innovative Approach for Improving Information Exchange between Palliative Care Providers in Slovenian Primary Health-A Qualitative Analysis of Testing a New Tool. HEALTHCARE. 2022;10(2). Located at: WOS:000976164400001. doi:10.3390/healthcare10020216

134.	Zheng Y, Head BA, Schapmire TJ. A systematic review of telehealth in palliative care: Caregiver outcomes. Telemedicine and e-Health. 2016;22(4):288–94. doi:10.1089/tmj.2015.0090

135.	Zimmermann J, Heilmann ML, Fisch-Jessen M, Hauch H, Kruempelmann S, Moeller H, et al. Telehealth Needs and Concerns of Stakeholders in Pediatric Palliative Home Care. Children. 2023;10(8):1315. doi:10.3390/children10081315
